# Glucose Lowering through Weight management (GLoW): a randomised controlled trial of the clinical and cost effectiveness of a diabetes education and behavioural weight management programme vs a diabetes education programme in adults with a recent diagnosis of type 2 diabetes

**DOI:** 10.1007/s00125-024-06355-6

**Published:** 2025-01-23

**Authors:** Julia Mueller, Penny Breeze, Francesco Fusco, Stephen J. Sharp, Katharine Pidd, Alan Brennan, Andrew J. Hill, Stephen Morris, Carly A. Hughes, Sarah E. Bates, Daniel Pollard, Jenny Woolston, Emma Lachassseigne, Marie Stubbings, Fiona Whittle, Rebecca A. Jones, Clare E. Boothby, Robbie Duschinsky, Jennifer Bostock, Nazrul Islam, Simon J. Griffin, Amy L. Ahern

**Affiliations:** 1 MRC Epidemiology Unit, School of Clinical Medicine, University of Cambridge, Cambridge, UK; 2 Sheffield Centre for Health and Related Research (SCHARR), School of Medicine and Population Health, University of Sheffield, Sheffield, UK; 3 Department of Public Health and Primary Care, University of Cambridge, Cambridge, UK; 4 Broadstreet Health Economics & Outcomes Research, Vancouver, BC Canada; 5 School of Medicine, University of Leeds, Leeds, UK; 6 Public Involvement Lead, Quality Safety and Outcomes Policy Research Unit, University of Kent, Oxford and Leeds, Kent, UK; 7 School of Primary Care, Population Sciences and Medical Education, University of Southampton, Southampton, UK

**Keywords:** Behavioural weight management, Diabetes mellitus, type 2, Obesity, Overweight, Randomised controlled trial, Weight loss, Weight reduction programmes

## Abstract

**Aims/hypothesis:**

UK standard care for type 2 diabetes is structured diabetes education, with no effects on HbA_1c_, small, short-term effects on weight and low uptake. We evaluated whether remotely delivered tailored diabetes education combined with commercial behavioural weight management is cost-effective compared with current standard care in helping people with type 2 diabetes to lower their blood glucose, lose weight, achieve remission and improve cardiovascular risk factors.

**Methods:**

We conducted a pragmatic, randomised, parallel two-group trial. Participants were adults (≥18 years) with overweight or obesity (BMI≥25 kg/m^2^) and recently diagnosed with type 2 diabetes (≤3 years), recruited from 159 primary care practices in England. We randomised participants to a tailored diabetes education and behavioural weight management programme (DEW; delivered by Weight Watchers) or to current standard care diabetes education (DE; Diabetes Education and Self Management for Ongoing and Newly Diagnosed [DESMOND] programme), using a computer-generated randomisation sequence in a 1:1 allocation stratified by gender and diabetes duration, unknown to those collecting and analysing the data. Participants could not be blinded due to the nature of the interventions. Participants completed assessments at 0, 6 and 12 months. The primary outcome was 12 month change from baseline in HbA_1c_. We also assessed bodyweight, blood pressure, cholesterol (total, HDL, LDL), glucose-lowering medication, behavioural measures (physical activity, food intake), psychosocial measures (eating behaviour, diabetes-related quality of life, wellbeing) and within-trial and modelled lifetime cost effectiveness.

**Results:**

We randomised 577 participants (DEW: 289, DE: 288); 398 (69%) completed 12 month follow-up. We found no evidence for an intervention effect on change in HbA_1c_ from baseline to 12 months (difference: −0.84 [95% CI −2.99, 1.31] mmol/mol, *p*=0.44) or 6 months (−1.83 [−4.05, 0.40] mmol/mol). We found an intervention effect on weight at 6 (−1.77 [−2.86, −0.67] kg) and 12 months (−1.38 [−2.56, −0.19] kg). Participants in DEW had a higher likelihood of achieving diabetes remission than participants in DE (6 months: RR 2.10 [95% CI 1.03, 4.47]; 12 months: RR 2.53 [1.30, 5.16]). DEW was cost-effective compared with DE in within-trial and lifetime analyses, in the latter generating an incremental cost effectiveness ratio of £2290 per quality-adjusted life year gained.

**Conclusions/interpretation:**

A commercial behavioural weight management programme combined with remote dietary counselling after diagnosis of type 2 diabetes did not improve HbA_1c_ up to 12 months post intervention in this trial. The intervention could help people achieve weight loss and be cost-effective compared with current standard National Health Service care.

**Trial registration:**

ISRCTN 18399564

**Funding:**

National Institute for Health and Care Research (NIHR; RP-PG-0216-20010), Medical Research Council (MC_UU_00006/6), NIHR Cambridge Biomedical Research Centre (NIHR203312).

**Graphical Abstract:**

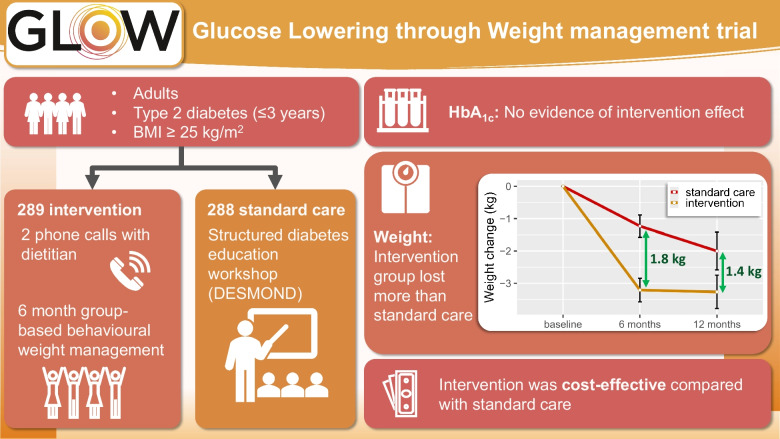

**Supplementary Information:**

The online version contains peer-reviewed but unedited supplementary material available at 10.1007/s00125-024-06355-6.


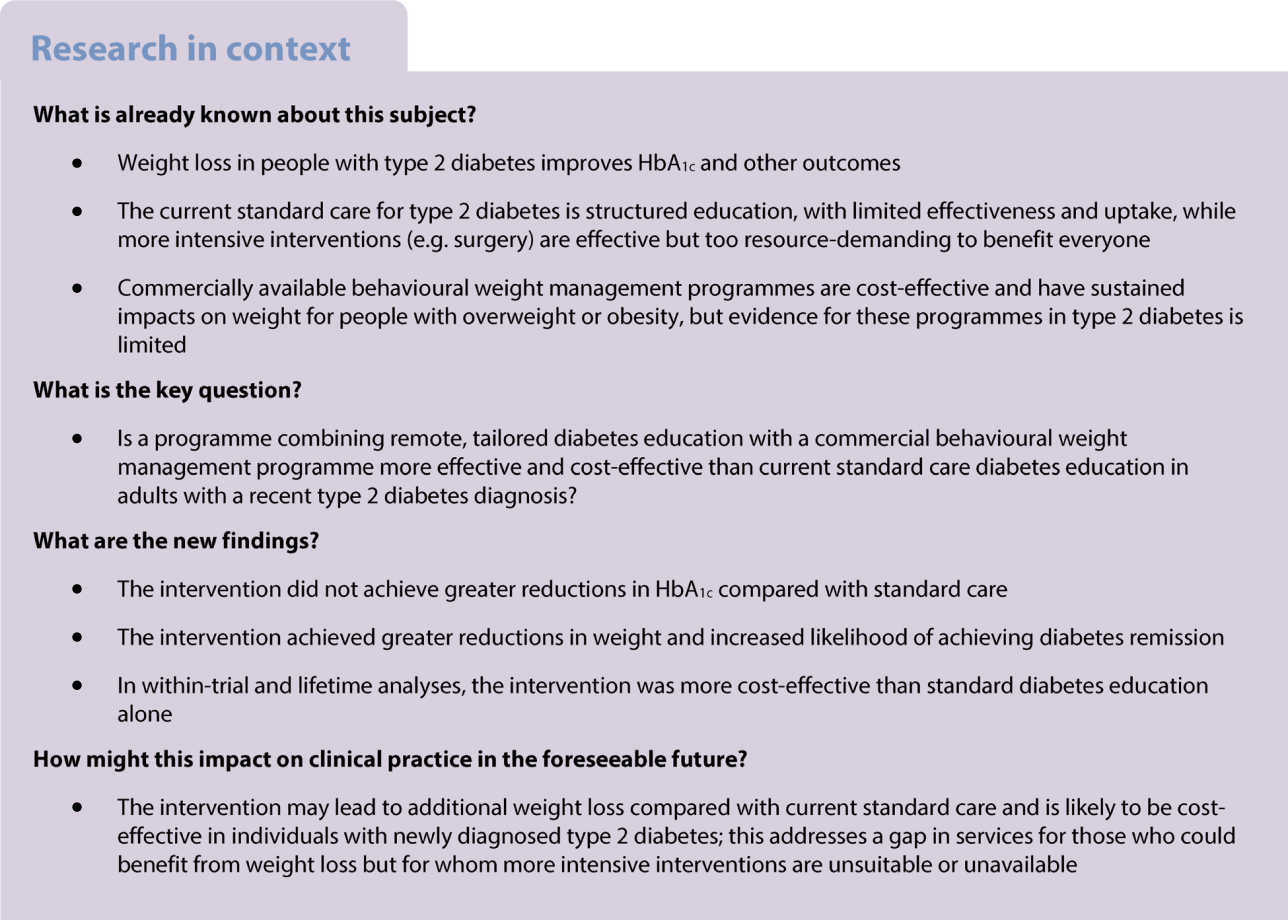


## Introduction

Type 2 diabetes is a risk factor for various health comorbidities, reduced quality of life and shorter life expectancy [1]. Weight loss achieved through total diet replacement (TDR) or intensive specialist-led behavioural interventions can improve glycaemic control and quality of life, reduce cardiovascular risks and lead to diabetes remission [2, 3]. However, these interventions are expensive and not available or suitable for all who might benefit from weight loss. Standard care for type 2 diabetes in the UK National Health Service (NHS) is structured diabetes education such as the Diabetes Education and Self Management for Ongoing and Newly Diagnosed (DESMOND) programme. While it is considered cost-effective, randomised controlled trials have found small, short-term weight losses with no reductions in HbA_1c_ and low uptake [4, 5]. Thus, intensive interventions may be available for a few individuals with type 2 diabetes, but most have access to short educational workshops with limited impact. This leaves a gap in services for individuals with newly diagnosed type 2 diabetes who could benefit from weight loss.

Commercial open-group behavioural weight management programmes, e.g. Weight Watchers (WW), are scalable and cost-effective in reducing weight and diabetes risk in people with overweight and obesity [6, 7], but have not been widely evaluated in adults with type 2 diabetes. A US trial showed that WW membership combined with remote dietary counselling led to greater weight loss and reductions in HbA_1c_ in adults with type 2 diabetes (including those with long-standing type 2 diabetes) at 12 months compared with usual care [8]. There is no evidence yet for the effect of this type of intervention earlier in the disease or for its cost effectiveness. We aimed to evaluate whether a programme combining remotely delivered tailored diabetes education with WW membership is more effective and cost-effective than structured diabetes education in supporting adults with a recent type 2 diabetes diagnosis to lower their HbA_1c_, lose weight and improve cardiovascular risk factors.

## Methods

### Study design

Glucose Lowering through Weight management (GLoW) was a pragmatic, randomised, single-blind, parallel-group, two-group, superiority trial. Participants identified from 159 primary care practices in England were randomised to a tailored diabetes education and behavioural weight management programme (DEW) or to standard care diabetes education (DE; i.e. the DESMOND programme). East of Scotland Research Ethics Service provided ethical approval (18/ES/0048). We prospectively registered the trial (ISRCTN registration no. 18399564) and published the protocol [9].

### Participants

Participants were adults (≥18 years) with overweight or obesity (BMI≥25 kg/m^2^) and a recent diagnosis of type 2 diabetes (≤3 years; confirmatory blood test not required). We recruited within 3 years of diagnosis to ensure participants were at a stage where national guidelines recommend referral to structured diabetes education. We included individuals who had received previous treatment for type 2 diabetes during these 3 years, excepting those listed in the exclusion criteria. Exclusion criteria were: using insulin; previous/planned bariatric surgery; current/planned pregnancy; current eating disorder diagnosis. Being in remission at baseline was not an exclusion criterion, as weight management could help maintain remission. We recruited from primary care practices identified through the National Institute for Health and Care Research (NIHR) Clinical Research Network that referred individuals with type 2 diabetes to DESMOND as standard care and had active WW groups in the local community. Participants were identified through electronic searches of primary care records and waiting lists for diabetes education. We also recruited via social media platforms.

### Randomisation and masking

We randomised participants to DEW or DE in a 1:1 allocation stratified by self-reported gender (male, female) and diabetes duration (<1 year, 1–3 years) with a block size of 6. The randomisation sequence was computer-generated by the trial statistician, programmed by the data manager and unknown to all other personnel, including those collecting data. Following allocation, it was not possible to blind participants or intervention providers. Investigators were blinded to intervention allocation until the database was locked and the primary analysis completed.

### Procedures

Following informed, written consent, participants were asked to attend measurement appointments at a participating primary care practice or research site at baseline, 6 months and 12 months. At each visit, trained staff took anthropometric measurements and blood samples according to the study protocol [9], and participants completed a self-report questionnaire in paper or online format. Participants unable or unwilling to attend a visit were asked to complete questionnaires and provide a self-measured weight. We measured physical activity using a wrist-worn triaxial accelerometer (Axivity AX3, Newcastle, UK), worn continuously for 7 consecutive days following each visit. Medical notes were reviewed to obtain last recorded weight, HbA_1c_, smoking status and diabetes status, prescribed medications and healthcare resource use, used to supplement missing data. Diabetes medication was independently managed by participants’ general practitioners and they were not given any instructions to change medications.

### Interventions

Details on the interventions are provided elsewhere [9]. Briefly, participants randomised to DEW (the ‘intervention’) received a structured diabetes education programme via two one-to-one telephone calls with a registered dietitian (provided by WW) and free membership of WW for 6 months, including access to community-based meetings and digital tools (e.g. the WW app).

Participants allocated to DE (‘control’) attended a 6 h diabetes education workshop (DESMOND) delivered by two trained healthcare professionals (usually a registered dietitian or diabetes nurse) in local healthcare or community venues in groups of up to ten participants. DE was provided as part of ‘usual care’. TIDieR checklists for the interventions are provided in electronic supplementary material (ESM) 1 (pp. 4–13).

### COVID-19 amendments

The GLoW study was paused on 16 March 2020 due to the COVID-19 pandemic and restarted on 4 January 2021 with protocol adaptations. Eligibility screening and consent forms were completed online. Participants received a kit of remote measures, which included a home-testing finger prick blood sample kit to measure HbA_1c_ (provided and analysed by The Doctors Laboratory, UK, accredited to the international standard for medical laboratories, ISO15189), self-report questionnaires and Axivity monitors. In DEW, in-person weekly meetings were replaced with virtual meetings. In DE, the in-person workshop was replaced with the MyDESMOND app with educational content, group dynamic and peer support, and interactive activities. Protocol amendments were reviewed and approved by the relevant research ethics committee.

### Outcomes

The primary outcome was 12 month change from baseline in HbA_1c_. Secondary anthropometric and biochemical outcomes were 6 month change from baseline in HbA_1c_ and 6 and 12 month changes from baseline in bodyweight, systolic and diastolic blood pressure, total cholesterol, HDL-cholesterol and LDL-cholesterol. We also assessed probability of achieving good glycaemic control (HbA_1c_<53 mmol/mol [7%] [10]), remission (HbA_1c_<48 mmol/mol [6.5%] and not prescribed glucose-lowering medication for the past 6 months) and losing ≥5% and ≥10% of initial body weight.

Secondary behavioural and psychosocial outcomes were 6 and 12 month changes from baseline in objective physical activity (using an accelerometer), self-reported physical activity (Recent Physical Activity Questionnaire [11]), self-reported dietary intake (European Prospective Investigation into Cancer [EPIC] Food Frequency Questionnaire [12]), dietary restraint (Three Factor Eating Questionnaire [13]), control over food cravings (Control of Eating Questionnaire [14]), binge eating (Binge Eating Scale [15]) and diabetes-related quality of life (Audit of Diabetes Dependent Quality of Life [16]). Data were unavailable to examine intervention effects on plasma carotenoids, body fat percentage and modelled cardiovascular risk. At baseline, participants completed a demographics questionnaire (self-reported gender, relationship status, ethnicity, religion, postcode for home and place of work). At 12 months, participants completed a programme evaluation questionnaire which included self-reported attendance and usage of programme features. We also obtained objective data on usage of the MyDESMOND app and the WW app, attendance at in-person WW meetings (data on virtual meetings were not available) and dietitian-reported completion of calls.

Health economic data were collected at baseline, 6 months and 12 months to include a Resource Use Questionnaire, self-reported out-of-pocket costs and the EuroQol–5 Dimension–5 Level instrument (EQ-5D-5L) [17]. We used participant medical notes and registry data to describe individual healthcare use.

### Statistical analysis

We required 576 participants to detect a difference between groups of 3 mmol/mol (2.4%) in HbA_1c_ with 90% power at a 5% significance level, assuming SD=16 mmol/mol of HbA_1c_ at follow-up, a 0.8 correlation between baseline and follow-up and 25% attrition [8].

We conducted clinical and microsimulation analyses using R (v4.2.1; https://cran.r-project.org/bin/windows/base/old/4.2.1/), and the within-trial cost effectiveness analysis using STATA14 (https://www.stata.com/stata14/). A detailed statistical analysis plan (SAP) and health economic analysis plan were finalised and uploaded to ISRCTN (https://www.isrctn.com/ISRCTN18399564) prior to commencing analyses.

Participants were included in the analysis in the group to which they were randomised, regardless of adherence to the programme. We estimated the intervention effect on HbA_1c_ at 12 months (and 95% CI) from a random intercepts linear regression model, using measures of change from baseline in HbA_1c_ at 6 months and 12 months as outcomes. The model included randomised group (intervention/control), timepoint, randomised group × timepoint interaction, the randomisation stratifiers (gender, diabetes duration) and baseline value of HbA_1c_ as fixed effects, and random intercepts to allow for the repeated measures on each individual. As pre-specified in the SAP, we repeated this analysis adjusting for duration of follow-up, and adjusting for glucose-lowering medication (categorised into increased/decreased/remained the same, see ESM 2.1, pp. 14–16). We conducted analyses with all observed data; random intercept models use all available data and assume missing data are missing at random (MAR). We performed a pre-specified sensitivity analysis using multiple imputation by chained equations (MICE) to impute missing values of HbA_1c_ at 12 months across the two groups. This assumes data are MAR. We investigated the impact of departures from this assumption using a pattern mixture model that allows data to be missing not at random (MNAR) by multiplying imputed values by a varying factor (0% [MAR], or increasing or decreasing the values by 10%, 20%, 30% [MNAR]) [18].

In a per-protocol analysis, we redid the primary outcome analysis including only those who took up their allocated programme (for definitions of uptake, see ESM 2.2, p. 17*).* We combined self-reported data with attendance data provided by WW and DESMOND.

We also conducted post hoc sensitivity analyses (pre-specified in the SAP) to assess potential effects of the COVID-19 pandemic, described in ESM 2.3 (p. 23).

We estimated the intervention effect on continuous secondary outcomes from random intercepts linear regression models, using the same approach as described for the primary analysis. For secondary binary outcomes, the SAP stated that we would use random intercept logistic regression models. However, we encountered issues with very large standard errors around parameters. It appeared that the model fitting algorithm did not converge to a satisfactory solution. We therefore ran separate logistic regression models for 6 and 12 months instead. The study was monitored by a Trial Steering Committee.

### Economic evaluation

We undertook a within-trial cost-utility analysis to compare DEW with DE from a UK NHS and Personal Social Services (PSS) perspective. Cost effectiveness was expressed as the incremental cost per quality-adjusted life year (QALY) gained, the incremental net monetary benefit (NMB), incremental cost per 1 mmol/mol decrease in HbA_1c_ and the incremental cost per 1 kg decrease in weight over a 12 month period. We estimated costs using intervention costs and individual-level data on healthcare use and medication costs. Health outcomes were described by health-related quality of life, HbA_1c_ and bodyweight collected in the trial (ESM 3, pp. 30–49). The intervention cost per participant of the DEW programme was £325 (£271 + value added tax [VAT]). The cost of the DE programme was £158, a weighted average of face-to-face (£265) and online (£12) delivery (ESM 4, pp. 50–52).

We evaluated the lifetime cost effectiveness of DEW compared with DE from an NHS and PSS perspective using an established microsimulation model [19]. Costs and QALYs were discounted at 3.5% in line with national guidelines using a microsimulation model. We generated a synthetic baseline population of 100,000 individuals from the characteristics of GLoW participants, supplemented with information from The Health Improvement Network [10, 20]. We generated long-term trajectories for metabolic risk factors and diabetes-related outcomes using the United Kingdom Prospective Diabetes Study (UKPDS) outcomes model risk equations [21]. These equations were modified to reduce the incidence of health outcomes that have been found to be overpredicted using the UKPDS risk equations [22]. An RR reduction for statin and anti-hypertensive use was added for risk of myocardial infarction (MI), stroke, congestive heart failure and mortality [23, 24]. MI and stroke were also modified using a calibration process as these are overpredicted using the UKPDS outcomes model and target data for these outcomes were identified from the ADDITION trial [25]. Diabetes complications were assigned healthcare costs and health-related quality of life decrements that contribute to the simulated lifetime NHS costs and QALYs. The model was tested through multiple validation methods, and full details of these are provided in ESM 5 (pp. 53–106). Modifications to the trajectories for BMI and HbA_1c_ alter simulated risk of diabetes-related complications, and subsequently impact on healthcare costs and QALYs. Simulated participants with HbA_1c_<48 mmol/mol (6.5%) at 12 months were assumed to have achieved diabetes remission, and the mean annual diabetes medication costs observed in the trial were removed from their diabetes-related costs. The risk of diabetes complications generated by the UKPDS equations was not modified by diabetes remission. The duration of intervention effect for BMI and HbA_1c_ was assumed to decline with time, with all effects removed by 10 years for BMI and 5 years for HbA_1c_ [22, 26]. Diabetes remission was simulated to end once simulated HbA_1c_ rose above HbA_1c_<48 mmol/mol (6.5%).

We used probabilistic sensitivity analysis to account for uncertainty in the model parameters. In our base case analysis, the eligible population receive DEW or DE at either face-to-face meetings or online with the proportions based on consultation with a service commissioner to reflect current care. We conducted sensitivity analyses in which the cost of DE is modified. We conducted additional subgroup analyses for diabetes duration (<1 year; 1–3 years), BMI categories (28–30 kg/m^2^; 30–35 kg/m^2^; 35–40 kg/m^2^; >40 kg/m^2^) and Index of Multiple Deprivation (IMD) quintiles [27]. We conducted additional sensitivity analyses to test modelling assumptions.

### Patient and Public Involvement

A diverse group of ten people with lived experience of type 2 diabetes and/or overweight/obesity attended regular meetings to review and advise on study design and participant-facing materials, interpret the findings and support dissemination. A Patient and Public Involvement (PPI) representative (JB, co-investigator) helped develop the protocol. Two PPI representatives sit on the Trial Steering Committee.

## Results

**Fig. 1:**
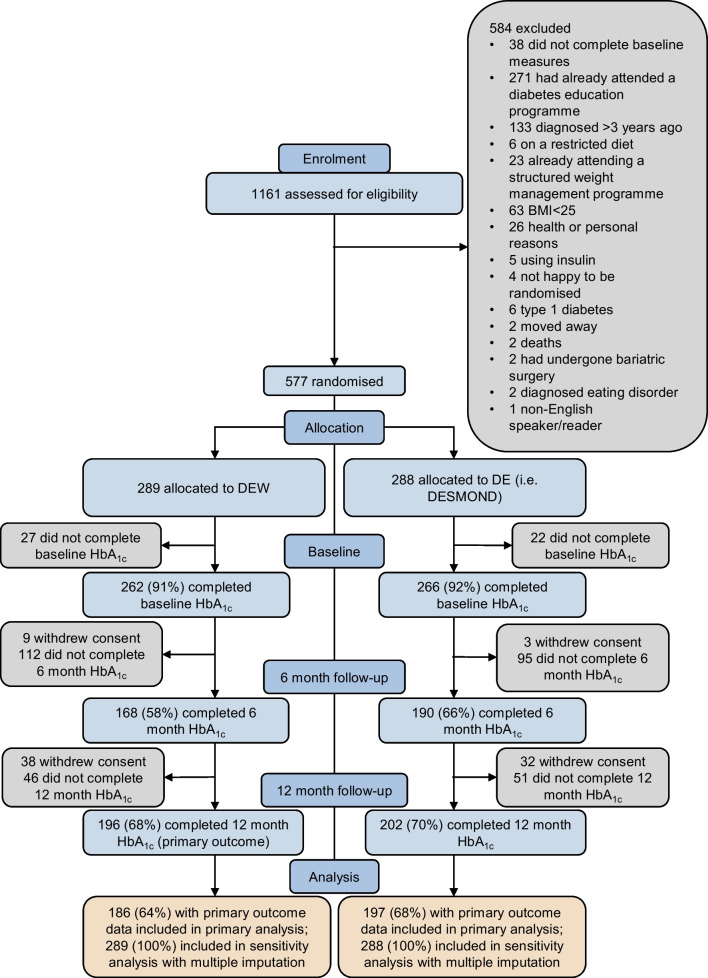
CONSORT flowchart

From 6 September 2018 to 6 August 2021, 1161 participants were assessed for eligibility, and 577 were randomised (Fig. 1); 204 (35.4%) were randomised after the trial was restarted following COVID-19 protocol amendments. Recruitment ended when the recruitment target was reached. Table 1 shows participant characteristics at baseline. HbA_1c_ values were obtained for 528 (91.5%) participants at baseline, 358 (62.0%) at 6 months and 398 (69.0%) at 12 months. Baseline characteristics for those with missing data on the primary outcome were similar across intervention groups and similar to baseline characteristics for those without missing data (ESM 2.2, p. 18). We were able to obtain intervention engagement data for 289 participants in DEW and 179 participants in DE. In DEW 60.6% (175/289) and in DE 50.3% (90/179, missing=109) took up the intervention (definition in ESM 2.2, p. 17).

**Table 1:** Baseline characteristics of participants

Characteristic	DEW, *n*=289		DE, *n*=288		Total sample, *N*=577	
	*n* or *n* (%)^a^	M (SD)	*n* or *n* (%)^a^	M (SD)	*n* or *n* (%)^a^	M (SD)
Age (years)	289	60.0 (12.8)	288	59.6 (12.4)	577	59.8 (12.6)
Baseline BMI (kg/m^2^)	288	34.3 (6.4)	288	34.9 (7.2)	576	34.6 (6.8)
HbA_1c_ (mmol/mol)	262	53.5 (12.9)	266	54.3 (14.2)	528	53.9 (13.6)
HbA_1c_ (%)	262	7.0 (3.3)	266	7.1 (3.4)	528	7.1 (3.4)
Good glycaemic control (HbA_1c_<53 mmol/mol)	160 (61.1)	–	163 (61.3)	–	323 (61.2)	–
Missing	27		22		49	
Weight (kg)	288	97.6 (20.1)	288	98.0 (20.9)	576	97.8 (20.5)
Cholesterol (mmol/l)	194	4.7 (1.0)	198	4.8 (1.1)	392	4.74 (1.1)
HDL-C (mmol/l)	182	1.3 (0.6)	199	1.2 (0.4)	390	1.25 (0.5)
LDL-C (mmol/l)	182	2.5 (0.8)	187	2.5 (0.9)	369	2.53 (0.9)
Triglycerides (mmol/l)	187	2.2 (1.2)	194	2.3 (1.2)	381	2.23 (1.2)
Systolic BP (mmHg)	220	134.3 (15.3)	220	134.8 (19.1)	440	134.5 (17.3)
Diastolic BP (mmHg)	220	81.0 (9.9)	219	80.6 (10.2)	439	80.8 (10.0)
Gender						
Male	137 (47.4)	–	139 (48.3)	–	276 (47.8)	–
Female	152 (52.6)	–	149 (51.7)	–	301 (52.2)	–
Ethnicity						
White	236 (91.1)	–	238 (90.8)	–	474 (91.0)	–
Black	6 (2.3)	–	13 (5.0)	–	19 (3.6)	–
Asian or Asian-British	15 (5.8)	–	7 (2.7)	–	22 (4.2)	–
Other ethnicity	2 (0.8)	–	4 (1.5)	–	6 (1.2)	–
Missing or prefer not to say	30		26		56	
Education						
Below post-secondary (up to and including A-levels)	132 (61.7)	–	122 (60.1)	–	254 (60.9)	–
Post-secondary (post A-levels)	82 (38.3)	–	81 (39.9)	–	163 (39.1)	–
Missing or prefer not to say	75	–	85	–	160	–
IMD quintile						
1	37 (14.4)	–	42 (16.6)	–	79 (15.5)	–
2	42 (16.3)	–	39 (15.4)	–	81 (15.9)	–
3	63 (24.5)	–	61 (24.1)	–	124 (24.3)	–
4	59 (23.0)	–	54 (21.3)	–	113 (22.2)	–
5	56 (21.8)	–	57 (22.5)	–	113 (22.2)	–
Missing or prefer not to say	32		35		67	
Diabetes duration						
Less than 1 year	154 (53.5)	–	158 (55.4)	–	312 (54.5)	–
1–3 years	134 (46.5)	–	127 (44.6)	–	261 (45.6)	–

^a^For the categorical variables, percentages within sub-categories are calculated using the number of non-missing values as the denominator

HDL-C, HDL-cholesterol; LDL-C, LDL-cholesterol; M, mean; '–' indicates data do not exist

### Primary outcome

From baseline to 12 months, we found no evidence for an intervention effect on change in HbA_1c_ (difference: −0.84 [95% CI −2.99, 1.31] mmol/mol, *p*=0.44). We also found no effect in the sensitivity analyses, across the different pattern mixture scenarios (ESM 2.2, p. 19*)* and in the per-protocol analysis (ESM 2.4, p. 25).

### Secondary biochemical outcomes and anthropometric outcomes

Mean changes in continuous biochemical/anthropometric outcomes in each study group are shown in ESM 2.2 (p. 21). We found no evidence for a difference between randomised groups in HbA_1c_ at 6 months (−1.83 [95% CI −4.05, 0.40] mmol/mol).

From baseline to 6 months, participants in DEW lost 1.77 (95% CI 0.67, 2.86) kg more than participants in DE. From baseline to 12 months, participants in DEW lost 1.38 (0.19, 2.56) kg more than participants in DE.

At 6 months, the likelihood of achieving ≥5% weight loss in DEW was 2.43 (95% CI 1.48, 4.04) times higher than in DE. The likelihood of achieving ≥10% weight loss was 3.15 (1.41, 7.72) times higher in DEW than in DE. At 12 months these effects were attenuated (Table 2).

**Table 2:** Categorical secondary outcomes at baseline, 6 months and 12 months by study group, and adjusted differences between the groups

Variable	Baseline		6 months		RR (95% CI)	12 months		RR (95% CI)
	*N*	*n* (%)	*N*	*n* (%)		*N*	*n* (%)	
Good glycaemic control (HbA_1c_<53 mmol/mol)								
DEW	262	160 (61.1)	168	125 (74.4)	1.43 (0.82, 2.52)	162	127 (78.4)	1.51 (0.80, 2.88)
DE	266	163 (61.3)	190	128 (67.4)	REF	174	120 (69.0)	REF
Losing ≥5% of initial body weight								
DEW	–	–	186	58 (31.2)	2.43 (1.48, 4.04)	146	47 (32.2)	1.08 (0.66, 1.78)
DE	–	–	196	29 (14.8)	REF	150	44 (29.3)	REF
Losing ≥10% of initial body weight								
DEW	–	–	186	23 (12.4)	3.15 (1.41, 7.72)	146	21 (14.4)	2.03 (0.95, 4.57)
DE	–	–	196	8 (4.1)	REF	150	11 (7.3)	REF
Diabetes remission (HbA_1c_<48 mmol/mol and not prescribed glucose-lowering medication for the past 6 months)^a^								
DEW	243	33 (13.6)	170	34 (20.0)	2.10 (1.03, 4.47)	199	40 (20.1)	2.53 (1.30, 5.16)
DE	252	42 (16.7)	195	34 (17.4)	REF	207	31 (15.0)	REF

^a^Participants in remission at baseline were included as remission was not the primary aim of the intervention, and participants in remission would still benefit from managing their weight/general lifestyle in order to maintain remission. At baseline, remission was based on prescriptions of glucose-lowering medication for the past 3 months; at 6 and 12 month follow-up, it was based on prescriptions over the past 6 months

REF, reference; '–' indicates data do not exist

In adjusted models, participants in DEW had 2.10 (95% CI 1.03, 4.47) times higher likelihood of achieving remission than participants in DE at 6 months; at 12 months, participants in DEW had 2.53 (1.30, 5.16) times higher likelihood of achieving remission than participants in DE (Table 2; also ESM 2.2, p. 20).

We did not find evidence for an effect of the intervention on secondary continuous biochemical outcomes (ESM 2.2, p. 21), or for a difference between groups in likelihood of achieving good glycaemic control (Table 2).

### Behavioural and psychosocial secondary outcomes

We found no evidence of an effect of randomised group on behavioural and psychosocial secondary outcomes, excepting a small effect on the rigid control dimension of dietary restraint at 6 months (ESM 2.2, p. 22).

### Post hoc sensitivity analyses

Post hoc sensitivity analyses to examine potential impacts of the COVID-19 pandemic are described in ESM 2.3 (pp. 23–24).

### Economic evaluation

The within-trial economic analysis (including intervention costs, primary care costs, secondary care costs and drug costs) showed that DEW had lower mean costs than DE over a 12 month period, and yielded marginally fewer QALYs (ESM 3, pp. 30–49): mean incremental costs for DEW vs DE were −£232; mean QALYs gained were −0.001 (95% CI −0.02, 0.03). The incremental NMB of DEW vs DE in the within-trial analysis was positive at cost effectiveness thresholds of £13,000, £20,000 and £30,000 per QALY gained. The probability that DEW was cost-effective vs DE at these cost effectiveness thresholds was 0.66, 0.64 and 0.62, respectively (ESM 3, p. 48).

Table 3 reports the main lifetime modelling results across three scenarios in which the intervention and comparator are delivered through a mix of face-to-face and online services. In the mixed scenario the model estimated a higher discounted lifetime cost for DEW compared with DE (incremental costs £81), with additional lifetime discounted QALYs per participant (0.0353 QALYs gained). This resulted in an expected incremental cost effectiveness ratio of £2290, and an expected incremental net benefit of £625. In the probabilistic sensitivity analysis, at a cost-per-QALY threshold of £20,000, DEW had a 97% probability of being cost-effective compared with DE (ESM 2.5, pp. 26–29). The estimates remained cost-effective across alternative cost scenarios for DE. The impact of subgroup and sensitivity analysis did not substantially impact the incremental cost effectiveness ratios (ESM 2.5, pp. 26–29).

**Table 3:** Lifetime discounted NHS and PSS costs and discounted QALYs and cost effectiveness estimates

Number of PSA samples for uncertainty analysis=2000	Total discounted NHS costs per person (£)	Total discounted QALYs per person	Incremental costs (£)	Incremental QALYs	Incremental expected NMB (£): £20,000 threshold	Incremental cost effectiveness ratio
Mixed F2F and online delivery						
DE	40,861	8.7008				
DEW	40,942	8.7361	81	0.0353	625	2290
F2F only service delivery						
DE	40,968	8.7008				
DEW	40,942	8.7361	−26	0.0353	732	Dominant
Online only service delivery						
DE	40,716	8.7008				
DEW	40,942	8.7361	226	0.0353	480	6410
Cost of DESMOND assuming optimistic uptake and full capacity for F2F (per person DESMOND cost £96)						
DE	40,799	8.7008				
DEW	40,942	8.7361	143	0.0353	563	4041

The cost of DESMOND in the optimistic scenario was estimated from a weighted average of 57.6% F2F DESMOND at £160.53 per person and 42.4% online at £8.48 per person, assuming higher rate of uptake of DESMOND and full capacity at F2F meetings

F2F, face-to-face; PSA, probabilistic sensitivity analysis

## Discussion

In this trial, we did not find evidence for differences in changes in HbA_1c_ over 12 months in people with overweight/obesity recently diagnosed with type 2 diabetes who were allocated to a tailored diabetes education and behavioural weight management programme compared with those allocated to structured diabetes education. However, those randomised to the intervention lost more weight and had more than twofold higher likelihood of achieving ≥5% weight loss and threefold higher likelihood of achieving ≥10% weight loss at 6 months than those allocated to structured diabetes education. When we assessed the impact of the interventions on health and healthcare costs during the within-trial period and over a lifetime, we showed that, due to the expected benefits of the achieved weight loss for wider health outcomes and healthcare use, tailored diabetes education combined with behavioural weight management would be cost-effective compared with current standard care.

We did not detect a significant intervention effect on our primary outcome, HbA_1c_ over 12 months. O’Neil et al compared a similar intervention (WW classes and remote dietary counselling) with standard care in the USA and found a group difference in HbA_1c_ of 4 mmol/mol (2.5%) over 12 months [8]. The GLoW trial was designed to have 90% power to detect a difference of 3 mmol/mol (2.4%); however, there was a slightly higher than anticipated attrition rate (31% vs 25%), and the CI around the estimated intervention effect was wide. Although our finding regarding HbA_1c_ is therefore inconclusive, the estimated intervention effect and CI were similar across a range of sensitivity analyses using either multiple imputation (assuming data are MAR) or pattern mixture models (allowing for departures from the MAR assumption).

We found small but significant reductions in weight in DEW compared with DE. Impacts on weight are of considerable importance in this population, since even modest weight loss can have beneficial effects on wider health outcomes such as quality of life and mobility [28].

While the reductions in weight were modest, they nevertheless constitute a significant improvement in outcomes compared with currently commissioned standard care, at a marginally higher cost (comparing £325 per participant for DEW with £265 for in-person DESMOND). Once primary care costs, secondary care costs and drug costs were also accounted for, DEW could be cost saving over a 12 month period compared with DE. The lifetime economic evaluation captures further cost savings over time from ongoing reduction in diabetes medications costs (simulated remission to a maximum of 4 years) and reduction in the risk of complications.

Previous studies have shown that TDR interventions and intensive, specialist-led lifestyle interventions can help individuals with type 2 diabetes to achieve weight loss and remission [2, 3]. However, these interventions are specialist-led and expensive and therefore difficult to scale. For example, the estimated costs are £1137 per participant for the intervention in the DIRECT trial (TDR + structured support) [29]. The GLoW trial indicates that a scalable, acceptable and less intensive intervention using remote dietitian consultations and a behavioural programme (both provided commercially) can lead to weight loss at a considerably lower cost of £325 per participant. While this cost is higher than for the current standard care programme, our within-trial and lifetime economic evaluations provided evidence that DEW was cost-effective compared with DE, and this finding was consistent across a range of scenarios and sensitivity analyses, including when DE was delivered online at a very low cost. In both the within-trial and lifetime analyses, the favourable economic position of DEW vs DESMOND was due to the substantial benefits of weight loss in this population and the associated improvements in health-related quality of life and cost savings.

Our significant effects on remission in the absence of effects on HbA_1c_ are challenging to explain. Remission was a binary outcome which took into account whether participants were taking glucose-lowering medication in the past 6 months, whereas HbA_1c_ was a continuous measure not adjusted for medication use. Therefore, it is possible to detect a significant effect in one and not the other. It is possible that the small reductions in weight in DEW led to some reductions in HbA_1c_, but that these were too limited to be detected with our sample size, yet nevertheless affected remission rates. However, this hypothesis is not verifiable based on the present data. We therefore recommend caution in interpreting these findings.

### Strengths and limitations

Our combined insights from the clinical and cost effectiveness analyses show that a model of care involving tailored diabetes education and commercial behavioural weight management is more effective than the currently commissioned DESMOND programme in helping people reduce their weight, and, due to the benefits of weight loss, is cost-effective across a range of scenarios and assumptions. By comparing this programme with a standard of care that is widely commissioned, findings are directly applicable to clinical practice and decision-making.

The COVID-19 pandemic led to disruptions of planned study procedures and intervention delivery. This likely led to increased attrition. We were unable to collect secondary biochemical outcomes (apart from HbA_1c_) during the pandemic due to restriction measures. Therefore, sample sizes for these outcomes were small. We deemed the proportion of missing data to be too high to render imputation of missing data appropriate.

We conducted sensitivity analyses to explore whether the COVID-19 pandemic impacted our results. Descriptively, intervention participants in the pre-pandemic group had a small decrease in HbA_1c_ while controls experienced an increase; post pandemic, both groups had an increase over 12 months. This suggests the intervention effects may have differed pre and post pandemic; however, we found no statistical evidence of an interaction.

We recruited a large sample of adults broadly generalisable to the UK population of adults living with type 2 diabetes; baseline characteristics, including distribution across IMD quintiles, are similar compared with population-based cohorts of adults living with type 2 diabetes in the UK [30, 31]. However, results may be less applicable to ethnic minority groups, as our sample included >90% White participants. We recruited a slightly lower proportion of men than seen in a nationally representative type 2 diabetes cohort (48% vs. 56%) [32]. We did not assess gender differences in intervention effects because our pre-specified SAP only planned to explore interaction effects with demographic variables if an overall effect was detected. Lower proportions of men are common in trials of behavioural weight management, but there is limited evidence to indicate how gender influences intervention effectiveness [32].

### Conclusion

We found no evidence that a model of care combining tailored diabetes education with a commercially available behavioural weight management programme achieved reductions in HbA_1c_ compared with standard care diabetes education, although high attrition for the primary outcome renders our findings inconclusive. We found that the intervention led to more weight loss and was likely to be cost-effective in the short term and longer term compared with standard care.

## Supplementary Information

Below is the link to the electronic supplementary material.

Supplementary file1 (PDF 2495 KB)

## Data Availability

The dataset analysed during the current study is not publicly available. Participant consent allows for data to be shared in future analyses with appropriate ethical approval, and the host institution has an access policy (https://www.mrc-epid.cam.ac.uk/wp-content/uploads/2019/02/Data-Access-Sharing-Policy-v1-0_FINAL.pdf) so that interested parties can obtain the data for replication or other research purposes that are ethically approved. Data access is available upon reasonable request (datasharing@mrc-epid.cam.ac.uk) and the data dictionary is available at https://epidata-ext.mrc-epid.cam.ac.uk/ddic/overview/GLOW/. Study documents (study protocol, statistical analysis plan, informed consent form, participant information sheet, analytic code) are available at https://www.isrctn.com/ISRCTN18399564 or on request. The fully executable code, simulated individuals and simulated parameters used to conduct the economic analyses are available under a GPL version 2 or later licence at: https://doi.org/10.15131/shef.data.24999692.v1

## Abbreviations

DE: Diabetes education (group)
DESMOND: Diabetes Education and Self Management for Ongoing and Newly Diagnosed
DEW: Diabetes education and behavioural weight management programme (group)
GLoW: Glucose Lowering through Weight management
IMD: Index of Multiple Deprivation
MAR: Missing at random
MI: Myocardial infarction
MNAR: Missing not at random
NHS: National Health Service
NMB: Net monetary benefit
PPI: Patient and Public Involvement
PSS: Personal Social Services
QALY: Quality-adjusted life year
SAP: Statistical analysis plan
TDR: Total diet replacement
UKPDS: United Kingdom Prospective Diabetes Study
WW: Weight Watchers
