# Supplementary material for: Glucose Lowering through Weight management (GLoW): a randomised controlled trial of the clinical and cost effectiveness of a diabetes education and behavioural weight management programme vs a diabetes education programme in adults with a recent diagnosis of type 2 diabetes
Source: Diabetologia. 2025 Jan 23;68(5):969–80. doi: 10.1007/s00125-024-06355-6 (PMC12021704; doi:10.1007/s00125-024-06355-6)
Supplement: Supplementary file 1 — Supplementary file1 (PDF 2495 KB) [file 125_2024_6355_MOESM1_ESM.pdf]

Electronic supplementary material

Glucose Lowering through Weight management (GLoW): a randomised controlled trial of the clinical and cost effectiveness of a diabetes education and behavioural weight management programme vs a diabetes education programme in adults with a recent diagnosis of type 2 diabetes

Contents

Electronic supplementary material (ESM) 1. Tidier Checklists. 4

ESM Table 1. TIDieR Checklist for tailored diabetes education and behavioural weight management programme (DEW) 4

ESM Table 2. TIDieR Checklist for DESMOND 8

ESM Table 3. TIDieR Checklist for MyDESMOND 11

Electronic supplementary material (ESM) 2. Supplementary information to the main trial analysis. 14

ESM 2.1: Medication coding 14

Step 1 14

ESM Table 4 14

Step 2 15

ESM Table 5. Drug codes, dosing information and removal of inconsistent reported doses. 15

Step 3 16

Step 4 16

Step 5 16

ESM 2.2: Supplementary Tables 17

ESM Table 6 17

ESM Table 7 18

ESM Table 8 18

ESM Table 9 19

ESM Table 10 20

ESM Table 11 21

ESM Table 12 21

ESM Table 13 22

ESM Table 14 23

ESM 2.3 Post-hoc sensitivity analyses 23

ESM Figure 1 24

ESM 2.4: Per-protocol analysis 25

ESM 2.5: Supplementary Economic evaluation Figures and Tables 26

Probabilistic Sensitivity Analysis 26

Scenario and sub-group sensitivity analysis 27
Electronic supplementary material (ESM) 3. Within-trial economic evaluation. 30
Methods 30
Overview 30
Resource use and costs 30
Utilities and QALYs 31
Dealing with missing data 32
Statistical methods 32
Sensitivity and sub-group analyses 33
Results 34
References 49
Electronic supplementary material (ESM) 4. Costs of delivering the DESMOND programme. 50
Description 50
ESM Table 24 51
ESM Table 25 52
Electronic supplementary material (ESM) 5: Modelled lifetime cost-effectiveness. 53
Baseline Population 53
Data 53
Sampling the Baseline Population 54
Exclusion Criteria 57
GP Attendance in General Population 58
Longitudinal Trajectories of Natural History metabolic Risk Factors 61
HbA_{1c} 62
BMI 63
SBP 63
High-density lipoprotein cholesterol (HDL-C) 64
Low-density lipoprotein cholesterol (LDL-C) 64
Other – Dynamic Modelling 65
Other - multivariable parametric proportional hazards survival models 65
Comorbid Outcomes and Mortality 67
Macrovascular Complications 68
Relative Risk of MI and Stroke 69
Microvascular Complications 70
Cancer 72
Breast cancer 72

Colorectal cancer 73
Osteoarthritis 74
Depression 75
Mortality 76
Calibration 77
Direct Health Care Costs 80
GP attendance 81
Diabetes 81
Statins 86
Anti-hypertensives 86
Cardiovascular Events 87
Osteoarthritis, Cancer, and Depression 89
Depression 90
Social Care costs 91
Osteoarthritis 91
Stroke 91
Utilities 91
Baseline Utility 92
Utility Decrements 92
Intervention Effects 93
Mixed-Effects Regression 94
Intervention effect beyond 12 months 96
Intervention Costs 97
Validation 99
Model Stability and Probabilistic Sensitivity Analysis 106
References 107

Electronic supplementary material (ESM) 1. Tidier Checklists.

ESM Table 1. TIDieR Checklist for tailored diabetes education and behavioural weight management programme (DEW)

Item, No	Item description	Intervention description
**Brief Name**		
1	Provide the name or a phrase that describes the intervention.	The tailored diabetes education and behavioural weight management (DEW) programme combines remote diabetes education and dietetic counselling with a supportive, group-based behaviour change programme.
**Why**		
2	Describe any rationale, theory, or goal of the elements essential to the intervention.	We have previously shown that commercial open-group behavioural weight management programmes, such as WW, are a scalable and cost-effective way to help people lose weight and reduce risk of type 2 diabetes (T2D). A randomised trial in the US showed that a combination of WW meetings and remote dietary counselling achieved greater weight losses and reductions in HbA_{1c} than standard care over 1 year in people with T2D. The tailored diabetes education and behavioural weight management (DEW) programme is a similar intervention that has been developed for use in the UK NHS. The DEW programme combines referral to WW with NICE-compliant diabetes education and dietary advice. Behaviour change techniques, such as goal-setting, self-monitoring and problem-solving, support the implementation of the educational components to facilitate effective self-management of T2D. The proposed mechanisms of action of this intervention include engagement with the intervention, increased physical activity, and improved diet quality (as evidence by increased fruit and vegetable intake, increased dietary restraint and increased control over food cravings).
**What**		
3	Materials: Describe any physical or informational materials used in the intervention, including those provided to participants or used in intervention delivery or in training of intervention providers. Provide information on where the materials can be accessed (such as online appendix, URL).	The DEW programme will be delivered through: i) telephone calls with a dietitian, ii) in-person, group-based sessions with WW, and iii) digital tools and online materials, which include food, weight and activity tracking, feedback loops, meal and recipe ideas and cooking skills, activity inspiration and videos, educational content, a closed digital community called ‘connect’ and access to an online coach 24/7 (service provided by real life coaches) for in the moment motivation and advice.
4	Procedures: Describe each of the procedures, activities, and/or processes used in the intervention, including any enabling or support activities.	Participants receive a triage call from a registered dietitian, with a specialist interest in diabetes, within two days of a referral, to explain the DEW programme and book their telephone sessions. The core QISMET-accredited structured diabetes

		education programme is then delivered to participants across two 1:1 telephone sessions with their dietitian. These telephone sessions last a total of 90 minutes combined. Telephone session one occurs within 10 days of referral to the programme and covers orientation and core curriculum topics. Telephone session two occurs within 10 days of telephone session one and covers further core curriculum topics and ways to tailor the WW programme for type 2 diabetes. Participants can contact their dietitian proactively during the intervention period for additional support where needed (and are encouraged to do so via the WW coach). Additional self-help education materials to support the curriculum are available and are delivered to all participants via email or mail, depending on the preference of the participant.<br><br>Following telephone session one, participants will be sent free membership of WW for six months. This includes access to group-based sessions and digital tools, including a website and an app.<br><br>WW sessions are held weekly in local community settings (e.g. schools, community centres) and last approximately one hour. They are open-group meetings (new people may join or leave the group at any time) and are led by a coach (trained lay person with experience of changing their lifestyle and losing weight on the WW programme). WW sessions include a confidential weigh-in with the coach and a 30-minute interactive education session led by the coach that includes advice on diet, physical activity, positive mindset, and behavioural strategies (e.g. goal setting, self-monitoring, problem solving, modifying the personal food environment, and relapse prevention). Peer support is available from other group members and coaches. Participants can contact their coach for support and advice between meetings. Participants can be accompanied to sessions by a friend, relative or carer.<br><br>Participants are also invited to join a closed social media group which is monitored and supported by a registered dietitian for any questions, frequent support and group social connections. The curriculum is also signposted to participants via this closed group to drive engagement.
**Who provided**		
5	For each category of intervention provider (such as psychologist, nursing	Dietitians: Details not provided

	assistant), describe their expertise, background, and any specific training given.	WW group coach: trained lay person with experience of changing their lifestyles and losing weight on the programme. Training not detailed.
**How**		
6	Describe the modes of delivery (such as face to face or by some other mechanism, such as internet or telephone) of the intervention and whether it was provided individually or in a group.	The core QISMET-accredited structured diabetes education programme is delivered individually to participants by a dietitian over two telephone calls. WW sessions are group-based and take place in-person at local community settings (e.g. schools, community centres). Participants also have access to digital tools, including the WW website and app, and a closed social media group. Additional self-help resources will emailed or mailed to each participant, depending on their preferences.
**Where**		
7	Describe the type(s) of location(s) where the intervention occurred, including any necessary infrastructure or relevant features.	The core QISMET-accredited structured diabetes education programme is delivered over the telephone. WW sessions are delivered in-person in local community settings (e.g. schools, community centres). Digital tools, such as the WW website and app, and the closed social media group, can be accessed by participants in their own homes through a computer, laptop, tablet and/or smartphone.
**When and How Much**		
8	Describe the number of times the intervention was delivered and over what period of time including the number of sessions, their schedule, and their duration, intensity, or dose.	The DEW programme is delivered over a 6-month period. It includes delivery of the core QISMET-accredited structured diabetes education programme across two telephone sessions, lasting a total of 90 minutes divided between the two calls. Telephone session one occurs within 10 days of referral and session two occurs within 10 days of session one. Following telephone session one, participants are sent free membership of WW for six months. This includes access to group-based sessions and digital tools, including a website and an app, which they can use at any time. WW session are held weekly, lasting approximately one hour each. Participants can contact their dietitian or WW coach proactively during the intervention period for additional support at any time when needed.
**Tailoring**		
9	If the intervention was planned to be personalised, titrated or adapted, then describe what, why, when, and how.	The core QISMET-accredited structured diabetes education programme has core topics to be delivered to each participant over the course of two telephone sessions. However, participants can contact their dietitian proactively during the intervention period for additional support where needed and can therefore receive tailored advice in addition to the standard diabetes education.

		Similarly, WW meetings include a confidential weigh-in with a coach and a 30-minute interactive education session at each session, which includes standard advice on diet, physical activity, positive mindset, and behavioural strategies (e.g. goal setting, self-monitoring, problem solving, modifying the personal food environment, and relapse prevention). However, participants can contact their WW coach for additional support and can therefore receive further tailored advice.
**Modifications**		
10	If the intervention was modified during the course of the study, describe the changes (what, why, when, and how)	Due to the COVID-19 pandemic, social distancing measures, including repeated cycles of lockdown, were put in place. This meant that WW group-based meetings had to transition to remote delivery using video software.
**How well**		
11	Planned: If intervention adherence or fidelity was assessed, describe how and by whom, and if any strategies were used to maintain or improve fidelity, describe them.	
12	Actual: If intervention adherence or fidelity was assessed, describe the extent to which the intervention was delivered as planned.	Dietician-reported attendance at dietitian calls<br>Data recorded by WW on attendance of group meetings

ESM Table 2. TIDieR Checklist for DESMOND

Item, No	Item description	Intervention description
**Brief Name**		
1	Provide the name or a phrase that describes the intervention.	The Diabetes and Education Self-Monitoring for Ongoing and Newly Diagnosed (DESMOND) programme is a structured diabetes education programme for people with a new diagnosis of type 2 diabetes (<3 years since diagnosis).
**Why**		
2	Describe any rationale, theory, or goal of the elements essential to the intervention.	As Type 2 diabetes is predominantly managed by the individual, with the support of their health care providers, structured education is required to support the individual in self-managing their condition, including on medication, diet, physical activity and self-monitoring. The DESMOND programme was developed by a multidisciplinary, multicentre collaborative that combined the experience of several centres already providing programmes for individuals newly diagnosed with type 2 diabetes. The philosophy of DESMOND is one of informed choice and patient empowerment, underpinned by a humanistic view of the individual. The content and processes of the programme is based on a series of psychological theories of learning: Leventhal's common sense theory, the dual process theory, and social learning theory.
**What**		
3	Materials: Describe any physical or informational materials used in the intervention, including those provided to participants or used in intervention delivery or in training of intervention providers. Provide information on where the materials can be accessed (such as online appendix, URL).	The DESMOND programme is delivered through in-person, group-based session/s (a total of 6 hours) over one day or two half-days. The education sessions are supported by specially developed resources.
4	Procedures: Describe each of the procedures, activities, and/or processes used in the intervention, including any enabling or support activities.	The DESMOND programme is a total of six hours, deliverable across one day or two half-days. Sessions are delivered in groups of up to 10 participants and are facilitated by two trained educators in local health care or community venues. Participants can bring a friend or partner with them.<br><br>The educators use a non-didactic approach, where learning is elicited rather than taught. The content covers thoughts and feelings about diabetes, understanding diabetes and glucose and what happens in the body, risk factors and complications associated with diabetes, monitoring and medication, food choices and physical activity, and

		planning for the future. Participants are also encouraged to consider medication. The programme activates participants to consider their own personal risk factors and choose a specific, achievable goal of behaviour change to work on.<br><br>The sessions are supported by specially developed resources.
**Who provided**		
5	For each category of intervention provider (such as psychologist, nursing assistant), describe their expertise, background, and any specific training given.	DESMOND educators complete two days of training.<br><br><u>Info taken from Skinner et al 2007:</u><br>To prepare them for delivering the programme, educators, who were a mixture of registered dieticians, practice nurses or nurse specialists, completed 2 days of training led by a consultant dietician, two diabetes nurse consultants, a consultant clinical psychologist and a chartered health psychologist, which included modelling of the program and interactive sessions. Throughout, the training modelled the style and methods used to deliver the patient program.<br><br>DESMOND educators receive formal training to deliver the programme and were supported by a quality assurance component of internal and external assessment to ensure consistency of delivery.
**How**		
6	Describe the modes of delivery (such as face to face or by some other mechanism, such as internet or telephone) of the intervention and whether it was provided individually or in a group.	The DESMOND programme is delivered in face-to-face groups of up to 10 participants in local health care or community venues by two educators. Participants also received specially developed resources to support the sessions.
**Where**		
7	Describe the type(s) of location(s) where the intervention occurred, including any necessary infrastructure or relevant features.	The DESMOND programme is delivered in local health care or community venues .<br><br>Specially developed resources can be accessed by participants.

**When and How Much**		
8	Describe the number of times the intervention was delivered and over what period of time including the number of sessions, their schedule, and their duration, intensity, or dose.	The DESMOND programme is delivered over a total of six hours: either across one day or two half-days.
**Tailoring**		
9	If the intervention was planned to be personalised, titrated or adapted, then describe what, why, when, and how.	The DESMOND programme has core topics to be delivered to each participant over the course of the one or two half-day sessions. All participants receive specially developed resources.
**Modifications**		
10	If the intervention was modified during the course of the study, describe the changes (what, why, when, and how)	Due to the COVID-19 pandemic, social distancing measures, including repeated cycles of lockdown, were put in place. This meant that many participants received a digital version of the programme, called MyDESMOND. This is described in a separate checklist.
**How well**		
11	Planned: If intervention adherence or fidelity was assessed, describe how and by whom, and if any strategies were used to maintain or improve fidelity, describe them.	Attendance data for in-person DESMOND workshops were not provided to the research team. The research team assessed attendance via a self-report questionnaire.
12	Actual: If intervention adherence or fidelity was assessed, describe the extent to which the intervention was delivered as planned.	

ESM Table 3. TIDieR Checklist for MyDESMOND

Item, No	Item description	Intervention description
**Brief Name**		
1	Provide the name or a phrase that describes the intervention.	MyDESMOND (Diabetes Education and Self-Management for Ongoing and Newly Diagnosed) is a digital self-management programme for people with type 2 diabetes.
**Why**		
2	Describe any rationale, theory, or goal of the elements essential to the intervention.	As type 2 diabetes (T2D) is predominantly managed by the individual, with the support of their health care providers, structured education is required to support the individual in self-managing their condition. MyDESMOND is a digital self-management programme for adults with T2D that was developed in response to barriers associated with the original in-person DESMOND programme (Diabetes Education and Self-Management for Ongoing and Newly Diagnosed), such as low uptake and attendance, suboptimal access, and limited capacity and infrastructure in the NHS to offer in-person programmes. MyDESMOND adopts a holistic approach to improve medical, behavioural, and emotional self-management of T2D, through improving knowledge, promoting self-care skills, increasing self-efficacy, and improving well-being. MyDESMOND was informed by several theoretical frameworks, including: i) Corbin and Strauss Model on Self-Management Framework, ii) the Capability-Opportunity- Motivation-Behaviour Model and the Taxonomy of Behaviour Change Techniques, iii) Health Action Process Approach Model, and iv) Social Support Theory.
**What**		
3	Materials: Describe any physical or informational materials used in the intervention, including those provided to participants or used in intervention delivery or in training of intervention providers. Provide information on where the materials can be accessed (such as online appendix, URL).	MyDESMOND is mobile app that is delivered through the Web and is accessible on any digital device (tablet, desktop computer or smartphone). The app includes several features and functions for users, including educational content, group dynamic and peer support, interactive activities whereby users can reflect on answers and increase their confidence, and goal setting to help with behaviour change.
4	Procedures: Describe each of the procedures, activities, and/or processes	• Referral and access info

	used in the intervention, including any enabling or support activities.	Bite-sized education sessions are released on the app each week . These short sessions consist of animations, gamification, quizzes, and games designed to be no more than 5 minutes in duration. Educational content covers the following topics: what is T2D, medication, complications of diabetes, food choices, physical activity, sedentary behaviour, emotions, diabetes-related distress, setting goals and relapse. Following completion of these sessions, users can then refresh their knowledge with weekly additional sessions for a further 8 weeks (each of which include educational material, an interactive quiz and animations). Additional features include health monitoring; step challenges and activity tracking to increase physical activity and focus on personal achievement; "Ask the expert" to provide users the opportunity to ask questions about their diabetes; a chat forum to share ideas, experiences, and questions with others who live with T2D; "Our buddies initiative" to allow users to invite family and friends to learn more about T2D and join the step challenges; and "Action planning and decision making" to help users to set realistic and achievable goals and tailor a personal action plan suitable to their environment and needs.
**Who provided**		
5	For each category of intervention provider (such as psychologist, nursing assistant), describe their expertise, background, and any specific training given.	Unclear. Not clear who the "expert" is in the "Ask the expert" feature.
**How**		
6	Describe the modes of delivery (such as face to face or by some other mechanism, such as internet or telephone) of the intervention and whether it was provided individually or in a group.	MyDESMOND is web-based app that is accessible on any digital device (tablet, desktop computer, and smartphone). It is used individually by each user.
**Where**		
7	Describe the type(s) of location(s) where the intervention occurred, including any necessary infrastructure or relevant features.	MyDESMOND is accessible on any digital device (tablet, desktop computer, and smartphone) and so can be used in any location with Internet connectivity.
**When and How Much**		
8	Describe the number of times the intervention was delivered and over	

	what period of time including the number of sessions, their schedule, and their duration, intensity, or dose.	
**Tailoring**		
9	If the intervention was planned to be personalised, titrated or adapted, then describe what, why, when, and how.	All users receive the same content and features via the app, which includes core structured diabetes education. However, users can use the "Ask the expert" feature to receive tailored advice and support.
**Modifications**		
10	If the intervention was modified during the course of the study, describe the changes (what, why, when, and how)	N/A
**How well**		
11	Planned: If intervention adherence or fidelity was assessed, describe how and by whom, and if any strategies were used to maintain or improve fidelity, describe them.	
12	Actual: If intervention adherence or fidelity was assessed, describe the extent to which the intervention was delivered as planned.	Objectively measured data on app (activated, completed, not activated)

Electronic supplementary material (ESM) 2. Supplementary information to the main trial analysis.

ESM 2.1: Medication coding

Medication coding for glucose-lowering medications was undertaken by the first author (JM), with guidance (and joint reviewing of specific cases) from SJG, Professor of General Practice with specialist expertise in Type 2 Diabetes. In the following "medication" refers specifically to glucose-lowering medication.

Step 1

Initially, JM reviewed free-text responses derived from GP notes reviews and extracted medication (ATC code), dose and frequency for each medication.
Where ambiguous dosage descriptions were given (e.g. "one tablet daily"), JM checked what dosage the participant was on at another timepoint and also what would be considered a "typical" dose based on ESM Table 4 below (created with the help of SJG) and made informed decisions. Any further ambiguous cases were reviewed together with SJG. Where no other data for the same medication were available for that participant, the typical dose from ESM Table 4 was used.
Example: The typical dose for A10BA02 would be 500mg, but if the participant was on 750mg at BL and 6m, and at 12m it said "one tablet daily", 750mg was used. In the absence of any other information, "one tablet daily" would be coded as 500mg.
Where participants had two entries for the same medication at the same timepoint, we used the first entry. There was no indication in the data whether one entry was made before or after the other.

ESM Table 4.

**ACT code**	**Name**	**Typical dose**
A10BA02	Metformin	500mg
A10BJ05	Dulaglutide	Once weekly injection 0.75mg
A10BK03	Empagliflozin	10mg; most people start on 10, not many go up to 25
A10BB09	Gliclazide	80mg
A10BH01	Sitagliptin	100mg once daily (never more than once a day)
A10BH04	Alogliptin	25mg a day
A10BH03	Saxagliptin	5mg once daily
A10BB12	Glimepiride	1mg a day, can go up to 4 times a day
A10BH05	Linagliptin	5mg once daily
A10BJ06	Semaglutide	Injection 0.25mg once weekly; can increase up to 1mg
A10BB07	Glipizide	2.5-5mg a day, can go up to 4 a day
A10AC01	Insulin (human)	Injection; short acting and longer acting; usually twice a day
A10BK02	Canagliflozin	100mg once daily (can go up to 300 daily)
A10BJ02	Liraglutide	0.6mg once daily can go up to 1.8mg<br>Injection once daily
A10BK01	Dapagliflozin	10mg once daily

Step 2

Outlier identification: We used the cut-offs below (ESM Table 5) to identify potential outliers. Identified cases were reviewed together with a clinician (SJG) against the original GP notes and cases were either edited or excluded as appropriate.

ESM Table 5. Drug codes, dosing information and removal of inconsistent reported doses.

code	drug name	DDD	DDD unit	BNF Drug Type	BNF Dose Information	Removing inconsistent reported doses.
a10ba02	metformin	2000	mg	Immediate release	Initially 500 mg once daily, then 500 mg twice daily, then 500 mg 3 times a day; maximum 2 g per day.	Remove any dose reported <200mg and>4000mg (inc. 1,15,60,1000000, potentially inconsistent measure)
a10bj05	dulaglutide	0.16	mg	Trulicity	0.75mg once weekly for T2D adults as monotherapy or 1.5mg once weekly for T2D adults alongside other antidiabetic medication; maximum 4.5mg per week.	Remove any dose reported <0.1mg and >0.64mg. (inc. 0.75 – Max daily dose from BNF is 0.64mg)
a10bk03	empagliflozin	17.5	mg	Jardiance	10 mg once daily, increased to 25 mg once daily if necessary and if tolerated.	
a10bb09	gliclazide	60	mg	Immediate release	Initially 40–80 mg daily, adjusted according to response, increased if necessary up to 160 mg once daily, dose to be taken with breakfast, doses higher than 160 mg to be given in divided doses; maximum 320 mg per day. Some patients are prescribed half a 40mg gliclazide tablet per day.	Remove any dose reported <20mg. (inc. 0.4 , smallest tablet dose offered in BNF is 30mg)
a10bh01	sitagliptin	100	mg		100 mg once daily	
a10bh04	alogliptin	25	mg		25 mg once daily	
a10bh03	saxagliptin	5	mg		5 mg once daily	
a10bb12	glimepiride	2	mg		Initially 1 mg daily, then increased in steps of 1 mg every 1–2 weeks, increased to 4 mg daily; maximum 6 mg per day.	
a10bh05	linagliptin	5	mg		5 mg once daily	
a10bj06	semaglutide	10.5	mg	By mouth	The starting dose for the oral version is 3mg/day, subsequently increasing to 7mg and then 14mg. In contrast, the starting dose for the subcutaneous injection version is 0.25 mg semaglutide once weekly. After 4 weeks the dose should be increased to 0.5 mg once weekly. After at least 4 weeks with a dose of 0.5 mg once weekly, the dose can be increased to 1 mg once weekly to further improve glycaemic control. After at least 4 weeks with a dose of 1 mg once weekly, the dose can be increased to 2 mg once weekly to further improve glycaemic control.	Remove any dose <0.25/7 = 0.036mg
a10bb07	glipizide	10	mg		Initially 2.5–5 mg daily, doses up to 15 mg may be given as a single dose, higher doses to be given in divided doses; maximum 20 mg per day.	
a10ac01	insulin (human)	40	U	Humulin - By subcutaneous injection, intramuscular injection, intravenous injection, or intravenous infusion		
a10bk02	canagliflozin	200	mg		100 mg once daily; increased if tolerated to 300 mg once daily if required	
a10ae06	insulin degludec	40	U			

a10bj02	liraglutide	1.5	mg	Victoza for T2D.	Initially 0.6 mg once daily for at least 1 week, then increased to 1.2 mg once daily for at least 1 week, then increased if necessary to 1.8 mg once daily.	Remove any dose reported >1.8mg. (inc. 6, maximum dosage BNF states is 1.8mg)
a10ab05	insulin aspart	40	U	Novorapid		

Step 3

Once all medications were clearly coded in terms of dosage and frequency, this was used to establish, for each medication separately, whether each participant (if they were on that particular medication) had an increase, decrease, or stayed the same from baseline to 12 months.
To do this, we first coded for each medication separately, whether each participant (if they were on that particular medication), had an increase/decrease/stayed same in terms of dose. We then worked out whether each participant had an increase/decrease/stayed same for dosage in general using the following logic:

- If there is a decrease in any one medication, code "decrease"
- If there is an increase in any one medication, code "increase"
- If someone has an increase on some medication(s) and a decrease on other(s), give priority to “decrease” (e.g., decrease in Med1, increase in Med2, code as decrease)
- All others coded as “stayed same”

Step 4

Next, we computed the number of medications each participant was on at each timepoint. We then coded whether each participant had an increase/decrease/stayed same in terms of number of medications from baseline to 12 months.

Step 5

We then combined the increase/decrease/stayed same variables for dosage and number of medications using the following logic (based on discussion with SJG and DEK, Academic Clinical Fellow, Cambridge University Hospitals NHS Foundation Trust):

- If dosage is same and number of meds is same = same
- If the dosage is same and number of meds increased = increase
- If the dosage is same and number of meds decreased = decrease

- If dosage is increased and number of meds is same = increase
- If dosage is increased and number of meds is decreased = decrease
- If the dosage is increased and number of meds increased = increase

- If dosage is decreased and number of meds is same = decrease
- If dosage is decreased and number of meds is decreased = decreased
- If the dosage is decreased and number of meds increased = increase

- If dosage is missing, code based only on number of medications

ESM 2.2: Supplementary Tables

ESM Table 6

Definitions for uptake, programme allocation timepoint, and mode of delivery for the two study groups, DEW and DE.

	**DEW**	**DE**
**Uptake**	Completing at least 1 dietitian call and at least 1 other form of engagement (attending a WW meeting virtually or in-person, or using the WW app at least once for any action, e.g. Weight tracking or food tracking).	Attending DESMOND or completing MyDESMOND online
**Primarily post onset of COVID-19 pandemic**	Since duration of access to WW sessions in DEW was 6 months, participants who received their referral 3 months or less prior to the onset of the pandemic (23rd March 2020, i.e. when the first lockdown was announced by the prime minister) will be considered to have spent ≥50% of their intervention during the pandemic (i.e. "primarily post onset of COVID-19 pandemic").	For DE, programme duration was shorter since, pre-pandemic, it involved only one full-day session. Therefore, participants who received their referral 1 month or less prior to the onset of the pandemic (23rd March 2020) will be considered "primarily post onset of COVID-19 pandemic".
**Intervention engagement type: Remote**	All WW sessions completed remotely OR only online materials accessed (no in-person WW sessions attended)	Only MyDESMOND accessed; no in-person DESMOND session attended
**Intervention engagement type: In-person**	All WW sessions completed in-person, online materials not accessed	DESMOND session(s) attended in-person, MyDESMOND not accessed
**Intervention engagement type: Mixed**	Some WW sessions attended online and some in person, OR WW sessions attended in person and online materials accessed	DESMOND session(s) attended in-person and MyDESMOND was accessed
**Intervention engagement type: Did not access programme**	No WW sessions attended and no online materials accessed	Did not attend DESMOND or access MyDESMOND

ESM Table 7

Intervention effects and costs inputs for the economic modelling

	**Mean**	**Standard error**	**Uncertainty distribution**
Difference in BMI (kg/m^2) between DEW and DE at 12 months	-0.51	0.258	NORMAL
Difference in HbA_{1c} (%) between DEW and DE at 12 months	-0.08	0.099	NORMAL
Diabetes remission cost saving	£33.89	5.64	NORMAL
DEW cost per participant	£325.20	N/A	Fixed cost
DE cost per participant face to face	£264.88	N/A	Fixed cost
DE cost per participant online	£12.47	N/A	Fixed cost
DE average cost per participant	£157.86	N/A	Fixed cost
Proportion DE face to face	58%	N/A	Fixed cost

ESM Table 8

Baseline characteristics of those with missing data by study group and for the total sample.

	With missing data in DE (control), n=86	With missing data in DEW (intervention), n=93	With missing data in total sample, N=179	Without missing data in total sample, N=398
Age (years) M(SD)	59.19 (13.28)	58.65 (13.21)	60.22 (12.33)	58.91 (13.21)
Gender (male)	48 (55.81%)	43 (46.24%)	172.00 (46.24%)	75.00 (49.02%)
Ethnicity (White)	64 (87.67%)	73 (92.41%)	337.00 (91.33%)	137.00 (90.13%)
IMD quintile(5)	14 (19.72%)	19 (24.05%)	80 (22.22%)	33 (22.00%)
Education (below post-secondary)	29 (56.86%)	37 (62.71)	188 (61.24%)	66 (60.00%)
Baseline BMI (kg/m^2)	35.44 (7.43)	34.52 (6.38)	34.44 (6.74)	34.96 (6.90)
Baseline BMI ≥ 30 kg/m^2	59 (68.60%)	69 (74.19%)	298.00 (75.06%)	128.00 (71.51%)
Baseline HbA_{1c} (mmol/mol)	58.58 (17.24)	55.92 (14.81)	52.64 (12.32)	57.19 (16.00)
Baseline HbA_{1c} (%)	7.5 (3.7)	7.3 (3.5)	7.0 (3.3)	7.4 (3.6)
Diabetes duration (less than 1 year)	44 (51.16%)	51 (54.84%)	221 (55.53%)	95 (53.07%)

ESM Table 9

Outputs of sensitivity analyses.

**Sensitivity analysis**	**Adjusted* difference between DEW and DE in change in** HbA_{1c} **from baseline to 12 months**
MICE with pattern mixture models for missing data of change in HbA_{1c} from baseline to 12 months	Values imputed using MICE:<br>-1.50 (95% CI: -3.76; 0.75) mmol/mol<br>**Pattern mixture**:<br>Missing values…<br>… 30% smaller: -1.31 (-3.29; 0.67)<br>… 20% smaller: -1.37 (-3.44; 0.69)<br>… 10% smaller: -1.44 (-3.59; 0.72)<br>… 10% larger: -1.57 (-3.93; 0.80)<br>… 20% larger: -1.63 (-4.11; 0.85)<br>… 30% larger: -1.69 (-4.29; 0.91)<br>than imputed values.
Adjustment of the primary analysis model for duration of follow-up	-0.83 (-3.06; 1.40) mmol/mol
Adjustment of the primary analysis model for glucose-lowering medication (stayed same/increase/decrease)	-1.09 (-3.48; 1.29) mmol/mol
Transformation of the primary outcome variable using cube root transformation	-0.17 (-0.16; 0.50)

* Adjusted for outcome at baseline and randomisation stratifiers. DEW = Diabetes education + behavioural weight management (intervention); DE = Standard care diabetes education (control); MICE = multiple imputation with chained equations

ESM Table 10

Remission cross-tabulation. The table includes all participants who had data for remission at baseline and 12 months.

		**DEW (Intervention)**		**DE (Control)**	
		**Baseline**		**Baseline**	
		**In remission**	**Not in remission**	**In remission**	**Not in remission**
12 months	In remission	14	21	17	6
	Not in remission	9	133	13	153

Remission = HbA_{1c} < 48 mml/mol and not currently prescribed glucose-lowering medication

ESM Table 11

Change in continuous biochemical and anthropometric outcomes from baseline to 6 and 12 months by study group, and adjusted differences between the groups in change from baseline.

**Outcomes**		**Change from baseline to 6 months**		**Adjusted difference[a] (95% CI)**	**Change from baseline to 12 months**		**Adjusted difference[a] (95% CI)**
		**N**	**Mean (SD)**		**N**	**Mean (SD)**	
HbA_{1c} (mmol/mol)	DEW	163	-2.55 (10.11)	-1.83 (-4.05; 0.40)	186	0.13 (11.50)	-0.84 (-2.99; 1.31)
	DE	184	-1.04 (9.88)		197	0.96 (14.25)	
HbA_{1c} (%)	DEW	163	-2.4 (3.1)	---	186	2.2 (3.2)	---
	DE	184	-2.2 (3.1)		197	2.2 (3.5)	
Weight (kg)	DEW	187	-3.21 (4.99)	-1.77 (-2.86;-0.67)	147	-3.27 (6.27)	-1.38 (-2.56; -0.19)
	DE	196	-1.23 (4.83)		151	-2.00 (7.17)	
Cholesterol (mmol/L)	DEW	82	-0.05 (0.68)	-0.03 (-0.24; 0.19)	79	-0.16 (0.87)	-0.09 (-0.31; 0.13)
	DE	87	-0.08 (0.79)		80	-0.09 (0.91)	
HDL-C (mmol/L)	DEW	82	-0.07 (0.86)	-0.04 (-0.19; 0.12)	78	0.19 (0.61)	-0.02 (-0.18; 0.14)
	DE	87	0.03 (0.16)		79	0.16 (0.66)	
LDL (mmol/L)	DEW	75	-0.07 (0.55)	-0.03 (-0.21; 0.16)	67	-0.11 (0.73)	-0.07 (-0.28; 0.13)
	DE	81	-0.07 (0.60)		63	0.01 (0.84)	
Triglycerides (mmol/L)	DEW	81	-0.01 (0.93)	-0.07 (-0.39; 0.24)	72	0.26 (1.23)	-0.07 (-0.40;0.27)
	DE	85	-0.01 (1.05)		71	0.17 (1.20)	
Systolic blood pressure (mmHg)	DEW	111	-2.11 (16.53)	-1.57 (-5.30; 2.14)	97	-0.78 (16.96)	1.35 (-2.55; 5.25)
	DE	108	-1.73 (17.09)		100	-1.33 (17.39)	
Diastolic blood pressure (mmHg)	DEW	109	-1.52 (8.09)	0.18 (-2.16; 2.51)	97	-1.29 (11.21)	0.05 (-2.40; 2.49)
	DE	108	-1.54 (9.51)		100	-1.37 (10.27)	

[a] Adjusted for outcome at baseline and the randomisation stratifiers (sex and diabetes duration [<1 year, 1-3 years]); N number; SD standard deviation; HDL-C high-density lipoprotein cholesterol; LDL-C low density lipoprotein cholesterol; kg kilograms; DE standard care diabetes education (DESMOND, control); DEW tailored diabetes education and behavioural weight management (intervention); CI confidence interval.

ESM Table 12

Medication changes from baseline to 12 months by study group.

**Medication change**	**DE**	**DEW**
**Same**	110 (81.5%)	87 (81.3%)
**Decreased**	12 (8.9%)	7 (6.5%)
**Increased**	13 (9.6%)	13 (12.2%)
**Missing**	153	182

Relative risk (RR) for having a decrease in glucose-lowering medication (compared to staying at the same level) over 12 months in DEW compared to DE: 0.47 [0.16; 1.39]; RR for having an increase: 1.31 [0.50; 3.43].

ESM Table 13

Change in self-reported behavioural and psychosocial outcomes from baseline to 6 and 12 months by study group, and adjusted differences between the groups in change from baseline.

**Outcomes**		**Change from baseline to 6 months**		**Adjusted difference (95% CI)**	**Change from baseline to 12 months**		**Adjusted difference (95% CI)***
		**N**	**Mean (SD)**		**N**	**Mean (SD)**	
Binge eating (Binge Eating Scale [BES])[28,29]	DEW	137	-2.11 (4.60)	-0.54 (-1.62; 0.53)	137	-1.93 (4.56)	-0.34 (-1.42; 0.75)
	DE	155	-1.57 (5.19)		143	-1.86 (5.56)	
Control over food cravings (Control of Eating Questionnaire)[27]	DEW	130	-3.24 (11.76)	-0.89 (-3.22; 1.44)	134	-2.19 (11.95)	0.41 (-1.96; 2.77)
	DE	151	-1.20 (11.61)		136	-2.42 (10.64)	
Capability/wellbeing (ICECAP-A)[30,34]	DEW	134	0.001 (0.12)	-0.01 (-0.04; 0.02)	142	-0.002 (0.14)	-0.004 (-0.03; 0.02)
	DE	155	0.01 (0.11)			0.002 (0.14)	
Flexible control dimension of dietary restraint (Three Factor Eating Questionnaire [TFEQ])[26]	DEW	134	1.01 (3.06)	0.50 (-0.07; 1.07)	135	0.21 (2.66)	-0.12 (-0.69; 0.46)
	DE	147	0.32 (2.78)		134	0.42 (2.68)	
Rigid control dimension of dietary restraint (Three Factor Eating Questionnaire [TFEQ])[26]	DEW	135	0.71 (2.76)	0.66 (0.10; 1.2)	137	0.03 (2.79)	0.02 (-0.54; 0.59)
	DE	150	-0.11 (2.76)		136	0.11 (2.62)	
Diabetes related quality of life (Audit of Diabetes Dependent Quality of Life [ADDQoL])[32]	DEW	120	0.31 (1.64)	0.25 (-0.12; 0.62)	120	0.07 (1.65)	-0.20 (-0.59-0.18)
	DE	124	0.10 (1.71)		119	0.44 (1.37)	
Self-reported physical activity energy expenditure (PAEE; Recent Physical Activity Questionnaire [RPAQ][23,24])	DEW	134	-2.22 (25.04)	-3.90 (-9.40; 1.60)	137	-0.29 (30.32)	0.01 (-5.57; 5.59)
	DE	148	2.47 (30.56)		137	4.07 (25.56)	
Objectively measured physical activity (Euclidian Norm Minus One [ENMO] in mg)	DEW	101	-0.38 (3.50)	-0.49 (-1.45; 0.46)	72	-0.02 (3.92)	-0.14 (-1.26; 0.99)
	DE	99	0.16 (3.51)		64	0.28 (3.46)	
Total energy intake in kilojoules (EPIC Food Frequency Questionnaire[FFQ])[25]	DEW	82	-986.21 (3414.72)	-590 (-1608.20; 427.44)	75	-981.06 (3537.32)	-327.44 (-1363.51;708.63)
	DE	92	-916.93 (5983.43)		81	-1066.05 (2860.51)	

* Adjusted for outcome at baseline and randomisation stratifiers. DEW = Diabetes education + behavioural weight management (intervention); DE = Standard care diabetes education (control)

ESM Table 14

Subgroup analyses.

		Adjusted difference (95% CI) in change in HbA_{1c} (baseline to 12 months)	Effect estimate for interaction term (randomised group * subgroups)	P-value for interaction term
**Intervention delivery timepoint**	Pre onset of the COVID-19 pandemic	-2.53 (-5.41; 0.35)	-2.96 (-6.84; 0.93).	0.14
	Post onset of the COVID-19 pandemic	1.82 (-1.34; 4.99)		
**HbA_{1c} source at 12 months**	Fingerprick self testing kit	-2.05 (-4.80; 0.69)	-1.07 (-5.51; 3.36)	0.64
	All other sources	1.48 (-2.41; 5.38)		
**Intervention engagement type**	In-person/mixed [a]	-1.17 (-4.80; 2.45)	REF	
	None	-2.86 (-6.79; 1.08)	-0.36 (-5.7; 4.97)	0.89
	Remote	3.36 (-1.67; 8.40)	3.65 (-2.33; 9.63)	0.23

[a] The in-person and mixed categories were amalgamated due to small sample sizes.

ESM 2.3 Post-hoc sensitivity analyses

We examined whether the estimated intervention effect on the primary outcome differed a) depending on whether participants were referred to their programmes such that they would have completed the programme primarily before or after the onset of the COVID-19 pandemic (for definitions see ESM-2.2, ESM *Table 6)*, b) depending on how participants engaged with the programme (remote, in-person/mixed, no engagement; ESM-2.2, ESM *Table 6*), and c) depending on measurement method for the primary outcome (home-testing finger-prick test vs. other methods).

Of 577 participants, 247 (42.8%) were referred to their allocated programme such that, if taken up, they would have completed their programme primarily post-onset of the COVID-19 pandemic (DEW: 137/289 [47.4%], DE: 110/288 [38.2%]). Among those who would have completed their programme primarily pre-onset of the pandemic, participants in DE had an increase in HbA_{1c} from baseline to 12 months of 1.11 mmol/mol [2.25%] (SD=16.11 [3.6%]), while participants in DEW had a decrease of 1.92 mmol/mol [2.33%] (SD=10.51 [3.11%]). Post-pandemic, both groups had an increase (DEW: 2.79 mmol/mol [2.41%]; SD=12.23 [3.27%]; DE: 0.69 mmol/mol [2.21%]; SD=10.21 [3.08%]) (ESM *Figure 1*). We found no evidence of an interaction between randomised group and pre- or post-pandemic programme referral, nor for an interaction between randomised group and HbA_{1c} source at 12 months, nor for an interaction between randomised group and intervention engagement type (ESM *Table 14*).

b) Pre-onset of the COVID-19 pandemic,

a) Post-onset of the COVID-19 pandemic,

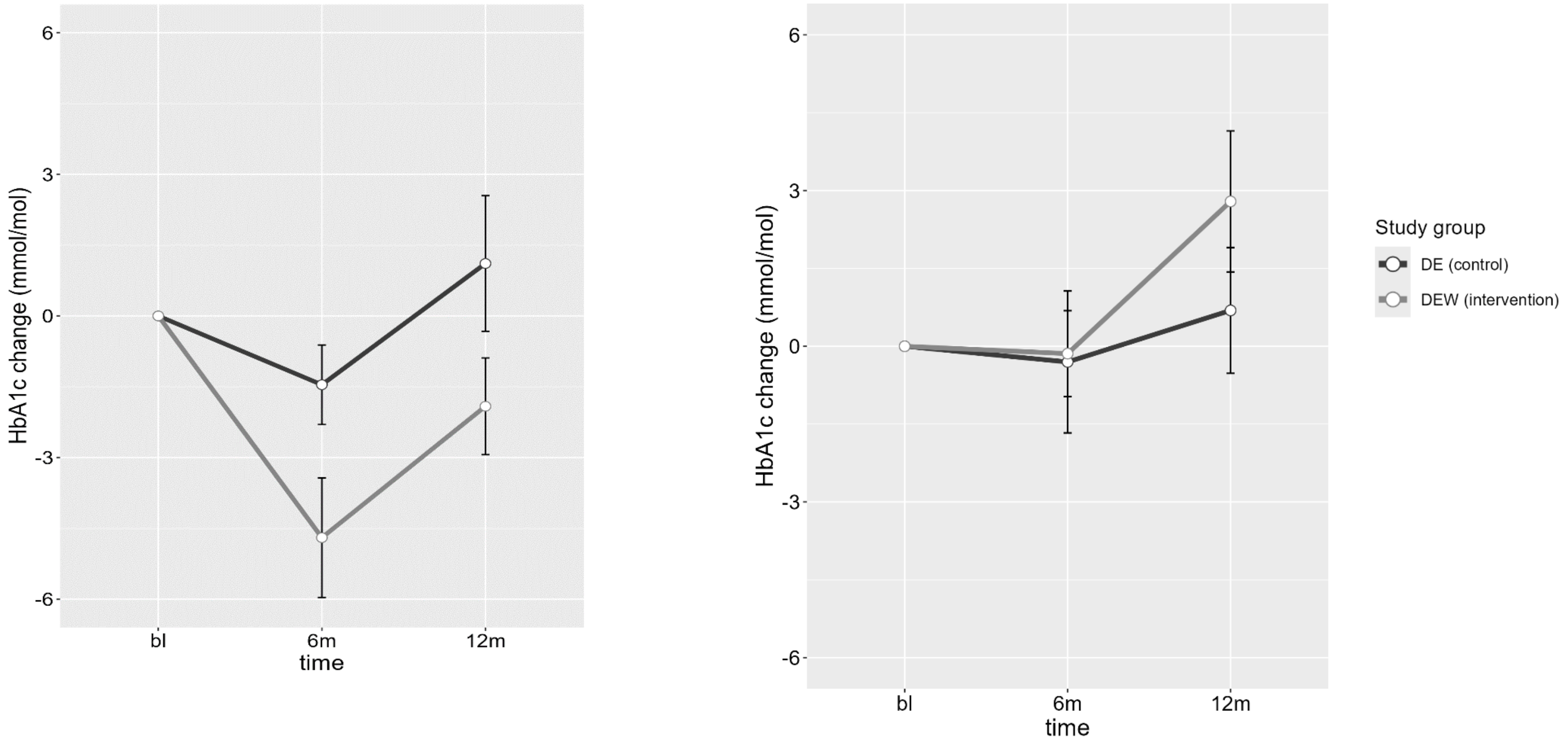

ESM Figure 1

Change in HbA_{1c} from baseline to 6 and 12 months; sample split into those who were referred to their intervention primarily pre vs. primarily post onset of the COVID-19 pandemic.

ESM 2.4: Per-protocol analysis

When including only those who took up their allocated programme (DEW: n=175/289, 60.6%; DE: n=90/179, 50.3%, missing=109), participants in DE had a reduction in HbA_{1c} of 1.88 mmol/mol [2.32%] (SD=10.91 [3.15%]), and participants in DEW had an increase of 0.50 mmol/mol [2.20%] (SD=12.33 [3.28%]; adjusted difference: 1.86 [-1.01; 4.72] mmol/mol).

ESM 2.5: Supplementary Economic evaluation Figures and Tables

Probabilistic Sensitivity Analysis

The results of the probabilistic sensitivity analysis indicate the uncertainty in the incremental costs and benefits of the DEW intervention when compared with DE. The PSA was analysed for 2000 samples of the model parameters. In the base case scenario the majority of sampled results fall below the £20,000 thresholds as illustrated in ESM Figure 1. The cost-effectiveness acceptability curve in ESM Figure 3 illustrate that the DEW is more likely to be cost-effective at a willingness to pay threshold above £5,000 per QALY.

ESM Figure 2
Incremental costs and incremental QALY scatter plot

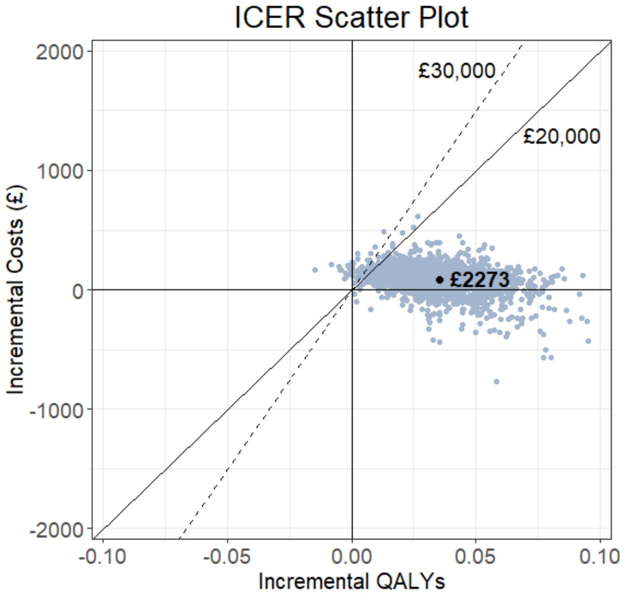

ESM Figure 3
Cost-effectiveness Acceptability Curve

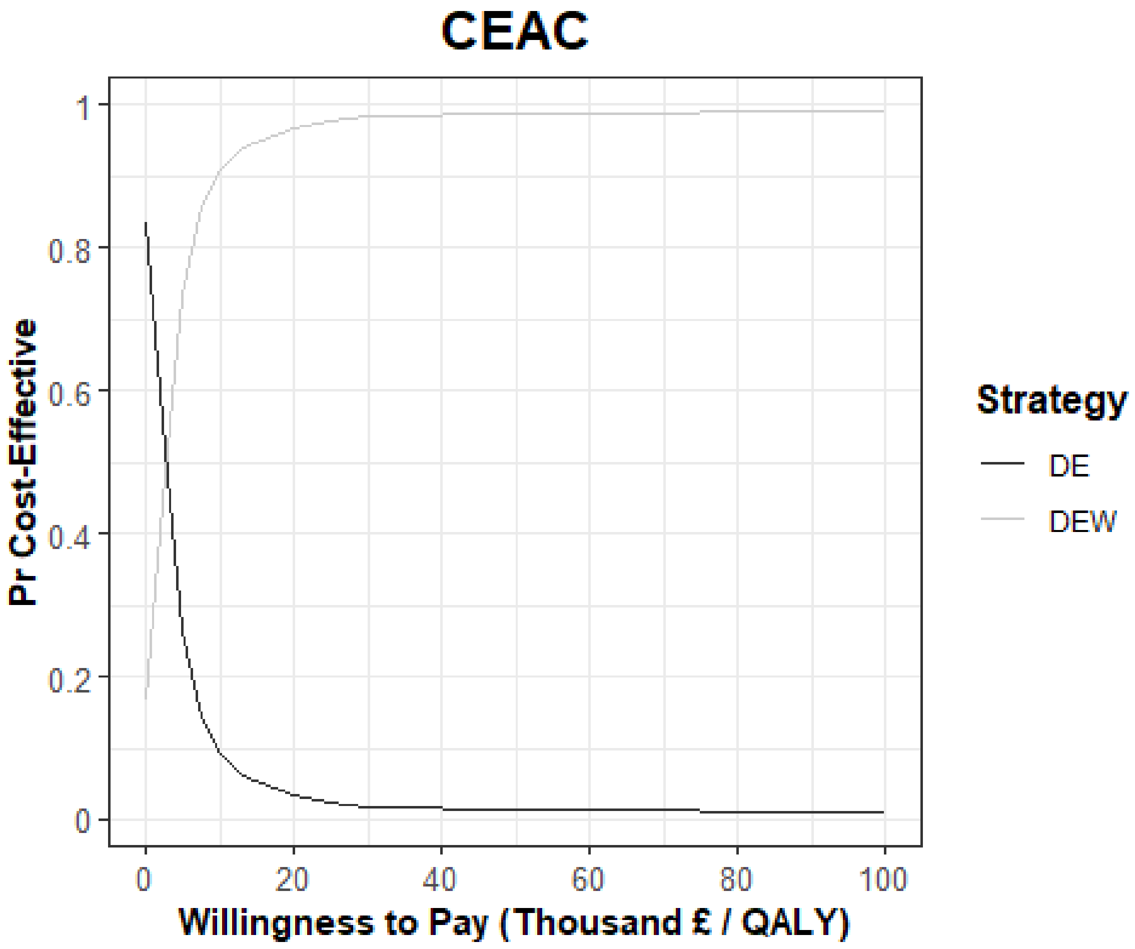

Scenario and sub-group sensitivity analysis

Sub-group sensitivity analysis identified that the expected net benefit of DEW increased for a population with high BMI, longer disease duration, and lower socioeconomic status (ESM Table 15).

ESM Table 15
Sub-group sensitivity analysis

**psa=2000**	**Total Discounted NHS Costs per person (£)**	**Total Discounted QALYs per person**	**Incremental Costs (£)**	**Incremental QALYs**	**Incremental expected net Monetary Benefit (£) – £20000 Threshold**
Base case ITT analysis					
DE	40861.29	8.7008	-	-	-
DEW	40942.11	8.7361	80.81	0.0353	625.00

Disease duration <=1 year					
DE	41045.33	8.6960	-	-	-
DEW	41133.04	8.7311	87.71	0.0352	615.47
Disease duration 2-3 years					
DE	40409.70	8.7238	-	-	-
DEW	40484.34	8.7593	74.64	0.0355	634.58
BMI groups: 28-30kg/m2					
DE	36866.50	8.8036	-	-	-
DEW	36972.85	8.8378	106.35	0.0342	576.82
BMI groups: 30-35kg/m2					
DE	39145.87	8.7912	-	-	-
DEW	39240.49	8.8261	94.62	0.0350	604.79
BMI groups: 35-40kg/m2					
DE	42151.30	8.7132	-	-	-
DEW	42228.82	8.7493	77.52	0.0361	644.17
BMI groups: 40kg/m2 +					
DE	46353.89	8.4922	-	-	-
DEW	46396.71	8.5296	42.82	0.0374	705.32
IMD Socioeconomic quantiles (1: most deprived)					
DE	42316.97	8.8870	-	-	-
DEW	42400.70	8.9227	83.73	0.0356	628.64
IMD Socioeconomic quantiles (2)					
DE	41610.89	8.8030	-	-	-
DEW	41692.58	8.8385	81.69	0.0355	628.05
IMD Socioeconomic quantiles (3)					
DE	40903.46	8.7279	-	-	-
DEW	40984.96	8.7634	81.50	0.0355	628.02
IMD Socioeconomic quantiles (4)					
DE	40181.27	8.6474	-	-	-
DEW	40262.18	8.6826	80.90	0.0352	622.72
IMD Socioeconomic quantiles (5: least deprived)					
DE	39282.24	8.5216	-	-	-
DEW	39361.93	8.5565	79.69	0.0349	619.00

Scenario analyses tested assumptions in the model that may have impacted on the overall findings of the analysis. Due to the time taken to analyse the results of the model only a limited number of scenario sensitivity analyses were conducted.

ESM Table 16
Scenario sensitivity analyses

psa=2000	Total Discounted NHS Costs per person (£)	Total Discounted QALYs per person	Incremental Costs (£)	Incremental QALYs	Incremental expected net Monetary Benefit (£) – £20000 Threshold
Base case ITT analysis					
DE	40861.29	8.7008	-	-	-
DEW	40942.11	8.7361	80.81	0.0353	625.00
Treatment effect maintenance 3 years					
DE	40861.29	8.7008	-	-	-
DEW	40965.47	8.7200	104.18	0.0192	279.45
Treatment effect maintenance 13 years					
DE	40861.29	8.7008	-	-	-
DEW	40880.59	8.7434	19.30	0.0426	833.26
Assume no utility adjustment for BMI					
DE	40861.29	9.6390	-	-	-
DEW	40942.11	9.6480	80.81	0.0090	99.06

Electronic supplementary material (ESM) 3. Within-trial economic evaluation.

Methods

Overview

We performed a within-trial cost-utility analysis to compare DEW versus DE from a UK NHS and Personal Social Services (PSS) perspective, using individual level data collected in the GLoW trial. The analysis was based on mean intervention costs for DEW and DE per participant, and participant-level NHS and social services resource use, health outcomes (changes in HbA_{1c}, changes in weight), and health related quality of life (HRQL) assessed in the trial at baseline, 6 and 12 months. The main outcome measure for the economic evaluation was quality-adjusted life years (QALYs), which combine length of life and quality of life, and is consistent with NICE recommendations.(1) Cost-effectiveness was expressed as the incremental cost per QALY gained and the incremental net monetary benefit (NMB).(1) We also calculated the incremental cost per 1mmol/mol decrease in HbA_{1c} and the incremental cost per 1kg decrease in weight. The time horizon was the within-trial period only (12 months), hence discounting of costs and benefits was not needed. All costs were reported in 2021/22 UK£ Sterling.

Resource use and costs

The cost of DEW was based on the price charged per participant by WW to deliver it. The cost of DE was calculated using a bottom-up costing exercise (ESM 4). For every participant we calculated the cost of contacts and drug use over 12 months using resource use data collected prospectively in the trial on primary and secondary care contacts and drug use. This included: general practitioner visits; nurse visits; NHS physiotherapist visits; NHS dietitian visits; NHS counsellor visits; visits to an NHS weight management service; visits to an education or peer support group; meals on wheels deliveries; home care attendant visits; other

health/social care worker contacts; inpatient hospitalisations; outpatient visits; accident and emergency department visits; day hospital visits; and, prescribed medications taken for reasons relating to weight and/or diabetes.

In addition, the following private (non-NHS/non-PSS) resource use data were collected but not included in the analysis given the costing perspective: visits to a commercial weight management group; visits to a private dietitian; visits to a personal trainer; visits to a private osteopath; visits to a private physiotherapist; visits to a private chiropractor; visits to a private chiropodist; visits to a private physiotherapist; over-the-counter (non-prescribed) products.

Unit costs were obtained from published sources,(2–4) inflated where appropriate to 2021/22 £ Sterling using NHS Pay and Prices Indices (ESM Table 17).(2) For inpatient stays a unit cost was applied using *NHS Reference Costs*(3) data based on the type of admission (elective stays, emergency stays of 2 or more nights, emergency stays of less than 2 nights). The costs of prescribed medications for weight and/or diabetes were determined using defined daily doses (DDD) of each drug and their respective unit costs in the UK.(5,6) DDDs were used to calculate the total drug administered to participants per each time point, and these estimates were then used to compute medication costs per participant.

The costs of all reported resource use were calculated for each participant. These figures were summed across cost components for each participant, to produce a total cost over the 12 month time horizon period for each individual participant.

Utilities and QALYs

Generic HRQL was described at baseline, 6 months and 12 months using the EQ-5D-5L descriptive system.(7) Each EQ-5D-5L health state was converted into a single summary index (utility value) applying a formula that attached weights to each of the levels in each dimension based on valuations by general population samples. We used a value set for the UK population to calculate utility values at each time point for every participant.(8) QALYs

were calculated for each individual patient assuming a straight-line relationship between the utility score at baseline, 6 months and 12 months.

Dealing with missing data

We assumed that missing data were missing at random, and multiple imputation was used to impute missing data on costs and utility scores at each time point. The equation used to impute missing data included baseline HbA_{1c}, baseline body weight, baseline utility scores, costs during the 3 months prior to baseline, gender, index of multiple deprivation quintile of place of residence, education qualifications, duration of diabetes, plus interaction terms between gender, index of multiple deprivation quintiles, education qualifications and, duration of diabetes with treatment group (to facilitate sub-group analyses). We used predictive mean matching to impute missing values, and generated 5 imputed datasets. Rubin's rules (9) were implemented for the subsequent analysis of multiple datasets.

Statistical methods

Analyses were performed on an intention-to-treat basis. Unadjusted means and standard deviations for resource use, utility scores and QALYs per participant based on the data collected at each follow-up point were estimated for each randomised group; values were unadjusted with no imputation for missing data at this stage. Mean differences and 95% confidence intervals of those differences between arms were also presented.

After accounting for missing data with multiple imputation, we calculated differences in mean costs and QALYs per group using ordinary least squares regression analysis. Incremental costs were adjusted for costs in the three-month period prior to baseline, baseline HbA_{1c}, baseline weight, gender, deprivation index, education, and time from diabetes diagnosis. QALYs gained were adjusted for baseline utility values, baseline HbA_{1c}, baseline weight, gender, deprivation index, education, and time from diabetes diagnosis.

Incremental NMBs were calculated as the difference in mean QALYs per participant with DEW versus DE multiplied by the maximum willingness to pay for a QALY minus the difference in mean costs per participant. We used cost-effectiveness thresholds of £13,000, £20,000 and £30,000 per QALY gained as willingness to pay values for a QALY. We ran 5,000 bootstrap replications nested within multiple imputation using the 5 imputed datasets(10) to calculate standard errors around mean values accounting for uncertainty in imputed values, the skewed nature of the cost data and utility values and sampling variation. 95% confidence intervals around the point estimates were calculated as the 2.5th and 97.5th percentiles of the distributions of each variable. To evaluate cost-effectiveness in terms of HbA_{1c} decrease and weight loss we used the effect sizes for these outcomes generated by the main statistical analysis (see main text). We calculated incremental cost-effectiveness ratios (ICERs) as the difference in costs between the two study arms divided by the difference in effects (QALYs gained, HbA_{1c} decrease, weight loss). We did not calculate 95% confidence intervals around the ICERs because the values were spread across all four quadrants of the cost-effectiveness plane, making interpretation problematic.

All statistical analyses were performed using Stata version 14.(11)

Sensitivity and sub-group analyses

As well as the base case cost-utility analysis (missing values imputed using MI, with adjustment for potential confounders, described above), cost-effectiveness estimates were also reported for three other scenarios: missing values imputed using multiple imputation but with no adjustment for potential confounders; no multiple imputation but adjustment for potential confounders; and, no multiple imputation or adjustment for potential confounders. For the base case, we constructed a cost-effectiveness acceptability curve(12) showing the probability that DEW was cost-effective compared with DE at a range of values for the cost-

effectiveness threshold for a QALY. These were generated from the positive incremental NMBs estimated across all 5 imputed datasets.(13)

In pre-defined sub-group analyses using the base case we investigated cost-effectiveness by gender, index of multiple deprivation, educational qualifications, and duration of diabetes. We used interaction terms between indicators for each sub-group category and treatment group.

In deterministic sensitivity analyses using the base case, we investigated how the incremental NMB (calculated at a cost-effectiveness threshold of £20,000 per QALY gained) varied according to different values of the incremental costs and QALYs gained, ranging from a 1.25 times decrement to a 1.25 times increment of the base case values. We also calculated the unit cost of the DEW programme required for it to be cost-effective (for the incremental NMB to equal zero at cost-effectiveness threshold values of £13,000, £20,000 and £30,000 per QALY gained).

Results

The intervention cost per participant of the DEW programme was £271 + Value Added Tax (VAT) (£325). The intervention cost of the DE programme was £159 (Appendix 4). Mean differences per participant across all NHS/PSS contacts at each follow-up point were small (all under 0.15; ESM Table 17), as were mean differences per participant in non-NHS/non-PSS resource use (all under 0.25; ESM Table 18). The unadjusted mean (95% confidence interval) cost difference for DEW versus DE, including intervention costs, primary care costs, secondary care costs and drug costs, was -£99 (-£1143 to £944; ESM Table 19). Mean utility scores remained constant over time in both groups over the 12 month period (range of mean values was 0.012 for DEW and 0.004 for DE), and the mean (95% confidence interval) QALY difference at 12 months for DEW versus DE was -0.008 (-0.068 to 0.052; ESM Table 20).

In the base case (with missing values imputed using multiple imputation, and adjusting for potential confounders) the mean incremental costs for DEW versus DE were -£232 (95% confidence interval -£1177 to £547), the mean QALYs gained were -0.001 (-0.02 to 0.03), and the incremental cost per QALY gained was £236,314 (note in this case that larger values of the ICER indicate better value for money; ESM Table 21). The incremental NMB was positive at each value of the cost-effectiveness threshold considered. The mean HbA_{1c} decrease for DEW versus DE was 0.84mmol/mol (-2.99 to 1.31) and mean weight loss was 1.38kg (0.19 to 2.56). DEW therefore dominated DE with regards to both these outcomes (i.e. was more effective and less costly). When considering different scenarios in terms of imputing missing data and adjusting for potential confounders, DEW was cost-effective when adjusting for potential confounders, but was not cost-effective when not adjusting for potential confounders, irrespective of adjusting or not for missing data. In all incremental analyses the 95% confidence intervals included 0.

There were variations by sub-group in point estimates of all incremental analyses (ESM Table 22). Trends according to cost-effectiveness were difficult to discern across categories of sub-groups, with the exception of gender; these findings suggest that DEW is likely to be cost-effective among women but not men.

The cost-effectiveness acceptability curves showed that at a maximum willingness to pay for a QALY of £13,000, £20,000 and £30,000 the probability that DEW is cost-effective versus DE was 0.66, 0.64 and 0.62, respectively (ESM Figure 4).

Varying the QALYs gained from DEW versus DE +/-1.25 times did not appreciably affect the incremental NMB (ESM Table 23). The incremental NMB was sensitive to varying the incremental costs by the same order of magnitude.

The intervention cost per participant of the DEW programme was £325 (£271 + VAT). If this increased to £544 then DEW would have an incremental cost per QALY gained equal to

£13,000. For incremental costs per QALY gained of £20,000 and £30,000 the analogous figures were £537 and £527, respectively. Therefore, as long as the intervention cost per participant of the DEW programme was less than £527 (£439 + VAT) then based on the point estimates, *ceteris paribus*, it is likely to be cost-effective.

ESM Table 17. Mean NHS/PSS contacts per participant and associated unit costs

	Mean (SD) [N]			Unit cost
	**-3 months to baseline**	**Baseline to 6 months**	**7 to 12 months**	
General practitioner visits				41
DE	0(0)[287]	0(0)[288]	0.031(0.395)[288]	
DEW	0(0)[289]	0.024(0.358)[289]	0.031(0.316)[289]	
Difference	-	0.024(95% CI: -0.017 to 0.066)	0(95% CI: -0.059 to .0058)	
Nurse visits				17
DE	0(0)[287]	0(0)[288]	0.07(0.759)[287]	
DEW	0.01(0.176)[289]	0.017(0.176)[289]	0.01(0.131)[289]	
Difference	0.01(95% CI: -0.01 to 0.031)	0.017(95% CI: -0.003 to 0.038)	-0.059(95% CI: -0.149 to 0.03)	
NHS physiotherapist visits				92
DE	0(0)[288]	0(0)[288]	0(0)[287]	
DEW	0(0)[288]	0.003(0.059)[289]	0.069(1.176)[289]	
Difference	-	0.003(95% CI: -0.003 to 0.01)	0.069(95% CI: -0.067 to 0.205)	
NHS dietitian visits				100
DE	0(0)[288]	0(0)[288]	0(0)[286]	
DEW	0(0)[288]	0.007(0.118)[289]	0(0)[289]	
Difference	-	0.007(95% CI: -0.007 to 0.021)	-	
NHS counsellor visits				53
DE	0(0)[288]	0(0)[288]	0(0)[286]	
DEW	0(0)[288]	0(0)[289]	0(0)[289]	
Difference	-	-	-	
Visits to an NHS weight management service				159
DE	0(0)[288]	0(0)[288]	0.042(0.71)[286]	
DEW	0(0)[289]	0.048(0.824)[289]	0(0)[289]	
Difference	-	0.048(95% CI: -0.047 to 0.144)	-0.042(95% CI: -0.124 to 0.04)	

Visits to an education or peer support group				25
DE	0(0)[288]	0(0)[288]	0.003(0.059)[286]	
DEW	0(0)[289]	0(0)[289]	0(0)[289]	
Difference	-	-	-0.003(95% CI: -0.01 to 0.003)	
Meals on wheels deliveries				7
DE	0(0)[288]	0(0)[288]	0(0)[286]	
DEW	0(0)[289]	0(0)[289]	0(0)[289]	
Difference	-	-	-	
Home care attendant visits				23
DE	0(0)[287]	0(0)[288]	0.085(1.424)[284]	
DEW	0(0)[287]	0(0)[287]	0(0)[287]	
Difference	-	-	-0.085(95% CI: -0.25 to 0.081)	
Other health/social care worker contacts				Various
DE	0.052(0.291)[287]	0.045(0.315)[288]	0.049(0.309)[288]	
DEW	0.059(0.363)[289]	0.063(0.413)[288]	0.093(0.929)[289]	
Difference	0.007(95% CI: -0.047 to 0.06)	0.017(95% CI: -0.043 to 0.077)	0.045(95% CI: -0.068 to 0.158)	
Inpatient hospitalisations				Elective stays 7,076
DE	0.031(0.174)[288]	0.038(0.209)[288]	0.052(0.278)[288]	Emergency stays 2+ nights 4,974
DEW	0.076(0.365)[289]	0.038(0.225)[289]	0.038(0.24)[289]	Emergency stays <2 nights 985
Difference	0.045(95% CI: -0.002 to 0.092)	0(95% CI: -0.036 to 0.035)	-0.014(95% CI: -0.057 to 0.028)	
Outpatient visits				235
DE	0.188(1.615)[282]	0.046(0.301)[260]	0.022(0.258)[270]	
DEW	0.221(1.838)[285]	0.036(0.268)[274]	0.057(0.334)[279]	
Difference	0.033(95% CI: -0.252 to 0.318)	-0.01(95% CI: -0.058 to 0.039)	0.035(95% CI: -0.015 to 0.085)	
Accident and emergency department visits				242
DE	0.011(0.133)[283]	0.019(0.185)[260]	0.007(0.122)[270]	
DE	0.057(0.333)[281]	0.026(0.277)[272]	0.011(0.182)[271]	
DEW	0.046(95% CI: 0.004 to 0.088)	0.007(95% CI: -0.033 to 0.047)	0.004(95% CI: -0.023 to 0.03)	

Day hospital visits				1224
DE	0.014(0.145)[282]	0.012(0.141)[252]	0.03(0.336)[265]	
DEW	0.039(0.257)[282]	0(0)[262]	0.131(10.844)[267]	
Difference	0.025(95% CI: -0.01 to 0.059)	-0.012(95% CI: -0.029 to 0.005)	0.101(95% CI: -0.124 to 0.326)	

NHS, National Health Service; PSS, personal social services; DE, current standard care diabetes education (DESMOND programme); DEW, tailored diabetes education and behavioural weight management programme, delivered by WW; SD, standard deviation; N, number of observations; CI, confidence interval. Unit costs are in 2022/22 UK£. The “Difference” rows show the values for DEW minus the values for DE; numbers may not sum due to rounding.

ESM Table 18. Mean private (non-NHS/non-PSS) resource use per participant

	Mean (SD) [N]		
	**-3 months to baseline**	**Baseline to 6 months**	**7 to 12 months**
Visits to a commercial weight management group			
DE	0.306(2.287)[288]	0.365(2.687)[288]	0.519(3.21)[287]
DEW	0.277(1.764)[289]	0.465(2.614)[286]	0.474(2.639)[287]
Difference	-0.029(95% CI: -0.363 to 0.305)	0.1(95% CI: -0.334 to 0.535)	-0.045(95% CI: -0.527 to 0.437)
Visits to a private dietitian			
DE	0.003(0.059)[288]	0(-)[288]	0(-)[288]
DEW	0(-)[289]	0(-)[289]	0(-)[289]
Difference	-0.003(95% CI: -0.01 to 0.003)	-	-
Visits to a personal trainer			
DE	0.017(0.212)[288]	0.118(1.775)[288]	0.083(1.414)[288]
DEW	0(-)[289]	0.035(0.342)[289]	0.076(1.184)[288]
Difference	-0.017(95% CI: -0.042 to 0.007)	-0.083(95% CI: -0.293 to 0.126)	-0.007(95% CI: -0.22 to 0.207)
Visits to a private osteopath			
DE	0.021(0.25)[288]	0.066(0.817)[288]	0.042(0.499)[288]
DEW	0.017(0.212)[289]	0.014(0.166)[289]	0(-)[289]
Difference	-0.004(95% CI: -0.041 to 0.034)	-0.052(95% CI: -0.149 to 0.044)	-0.042(95% CI: -0.099 to 0.016)
Visits to a private physiotherapist			
DE	0.007(0.118)[288]	0.003(0.059)[288]	0.038(0.428)[288]
DEW	0.014(0.186)[289]	0.014(0.235)[289]	0.052(0.727)[289]
Difference	0.007(95% CI: -0.019 to 0.032)	0.01(95% CI: -0.018 to 0.038)	0.014(95% CI: -0.084 to 0.111)
Visits to a private chiropractor			
DE	0.007(0.118)[288]	0.017(0.243)[288]	0.007(0.118)[288]
DEW	0(-)[289]	0(-)[289]	0(-)[288]
Difference	-0.007(95% CI: -0.021 to 0.007)	-0.017(95% CI: -0.045 to 0.011)	-0.007(95% CI: -0.021 to 0.007)

Visits to a private chiropodist			
DE	0.038(0.28)[288]	0.066(0.464)[288]	0.09(0.486)[288]
DEW	0.076(0.458)[289]	0.059(0.363)[289]	0.097(0.605)[289]
Difference	0.038(95% CI: -0.024 to 0.1)	-0.007(95% CI: -0.075 to 0.061)	0.007(95% CI: -0.083 to 0.096)
Visits to a private physiotherapist			
DE	0.066(0.751)[288]	0.063(0.901)[288]	0.167(2.828)[288]
DEW	0.045(0.56)[289]	0.01(0.176)[289]	0.007(0.118)[289]
Difference	-0.021(95% CI: -0.129 to 0.087)	-0.052(95% CI: -0.158 to 0.054)	-0.16(95% CI: -0.487 to 0.168)
Number of over-the-counter (non-prescribed) products			
DE	0.3(1.798)[287]	0.337(2.033)[288]	0.38(2.865)[284]
DEW	0.295(1.616)[288]	0.582(3.923)[287]	0.511(2.763)[284]
Difference	-0.005(95% CI: -0.285 to 0.276)	0.245(95% CI: -0.267 to 0.757)	0.13(95% CI: -0.334 to 0.594)

NHS, National Health Service; PSS, personal social services; DE, current standard care diabetes education (DESMOND programme); DEW, tailored diabetes education and behavioural weight management programme, delivered by WW; SD, standard deviation; N, number of observations; CI, confidence interval. The "Difference" rows show the values for DEW minus the values for DE; numbers may not sum due to rounding.

ESM Table 19. Mean costs per participant from baseline to 12 months

	**Mean (SD)[N]**
Primary care costs	
DE	16(183)[284]
DEW	13(139)[281]
Difference	-2(95% CI: -29 to 24)
Secondary care costs	
DE	269(1349)[230]
DEW	316(1839)[241]
Difference	46(95% CI: -245 to 337)
Drugs costs	
DE	2185(5668)[288]
DEW	2140(5667)[289]
Difference	-44(95% CI: -971 to 882)
Total costs	
DE	2370(6223)[227]
DEW	2104(5128)[236]
Difference	-265(95% CI: -1308 to 778)
Total costs plus intervention costs	
DE	2529(6223)[227]
DEW	2430(5128)[236]
Difference	-99(95% CI: -1143 to 944)

DE, current standard care diabetes education (DESMOND programme); DEW, tailored diabetes education and behavioural weight management programme, delivered by WW; SD, standard deviation; N, number of observations; CI, confidence interval. Costs are in 2022/22 UK£. The “Difference” rows show the values for DEW minus the values for DE. Note the numbers across cost components do not sum due to missing data and different denominators.

ESM Table 20. Mean utility scores and QALYs per participant

	Mean EQ-5D-5L scores (SD) [N]			Mean QALYs (SD) [N]
	**Baseline**	**6 months**	**12 months**	
DE	0.715(0.246)[219]	0.718(0.254)[177]	0.714(0.261)[162]	0.719(0.246)[124]
DEW	0.696(0.243)[226]	0.708(0.265)[158]	0.706(0.267)[161]	0.711(0.224)[112]
Difference	-0.019(95% CI: -0.064 to 0.027)	-0.01(95% CI: -0.066 to 0.046)	-0.008(95% CI: -0.066 to 0.049)	-0.008(95% CI: -0.068 to 0.052)

EQ-5D-5L, EuroQoL 5 dimension 5 levels; QALY, quality adjusted life year; DE, current standard care diabetes education (DESMOND programme); DEW, tailored diabetes education and behavioural weight management programme, delivered by WW; SD, standard deviation; N, number of observations; CI, confidence interval. The "Difference" rows show the values for DEW minus the values for DE. Note the QALYs do not reflect the aggregate of the utility scores at each time point due to missing data and different denominators.

ESM Table 21. Incremental cost-effectiveness of DEW versus DE

	Incremental analysis (SE)[95% CI]									
	**Incremental costs**	**QALYs gained**	**HbA_{1c} decrease (mmol/mol)**	**Weight loss (kg)**	**Incremental cost per QALY gained**	**Incremental cost per 1mmol/mol reduction in HbA_{1c}**	**Incremental cost per 1kg reduction in weight**	**iNMB**		
								**£13,000**	**£20,000**	**£30,000**
Base case[a]	-232 (487) [-1177 to 547]	-0.001 (0.01) [-0.02 to 0.03]	0.84 (1.10) [-2.99 to 1.31]	1.38 (0.83) [0.19 to 2.56]	236,314	DEW dominates DE	DEW dominates DE	219 (514) [-550 to 1310]	212 (549) [-679 to 1421]	202 (619) [-892 to 1622]
No adjustment[b]	-21 (498) [-928 to 893]	-0.01 (0.02) [-0.05 to 0.03]			1,593			-151 (592) [-1142 to 1119]	-244 (682) [-1361 to 1255]	-377 (839) [-1801 to 1508]
Complete case analysis[c]	-304 (556) [-1204 to 981]	0.001 (0.01) [-0.02 to 0.02]			DEW dominates DE			320 (592) [-1044 to 1285]	329 (627) [-1088 to 1354]	342 (692) [-1211 to 1493]
Complete case analysis, no adjustment[d]	-99 (554) [-1089 to 1109]	-0.01 (0.03) [-0.06 to 0.07]			12,476			-4 (755) [-1445 to 1521]	-60 (928) [-1746 to 1885]	-140 (1208) [-2219 to 2487]

QALY, quality adjusted life year; iNMB, incremental net monetary benefit; DE, current standard care diabetes education (DESMOND programme); DEW, tailored diabetes education and behavioural weight management programme, delivered by WW; SE, standard error; CI, confidence interval. All incremental analyses show the values for DEW minus the values for DE. Costs are in 2022/22 UK£. iNMBs are calculated at cost-effectiveness thresholds of £13,000, £20,000 and £30,000 per QALY gained.

[a] Data include values imputed using multiple imputation (see text). Figures are adjusted for potential confounders (see text).

[b] As for the base case except there is no adjustment for potential confounders.

[c] As for the base case except there is no multiple imputation of missing values

[d] As for the base case except there is no multiple imputation of missing values or adjustment for potential confounders

ESM Table 22. Incremental cost-effectiveness of DEW versus DE by sub-group

**Sub-group**	**Incremental costs**	**QALYs gained**	**Incremental cost per QALY gained**	**iNMB**		
				**£13,000**	**£20,000**	**£30,000**
Gender						
Male	£401	-0.030	DE dominates DEW	-£792	-£1,003	-£1,303
Female	-£412	0.003	DEW dominates DE	£454	£476	£508
Deprivation						
Most deprived (quintile 1)	-£356	0.042	DEW dominates DE	£905	£1,201	£1,623
Deprivation quintile 2	-£31	-0.048	645	-£597	-£935	-£1,418
Deprivation quintile 3	£1,056	0.001	1056027	-£1,049	-£1,046	-£1,040
Deprivation quintile 4	-£730	-0.007	104332	£638	£588	£517
Least deprived (quintile 5)	-£829	-0.064	12948	-£3	-£450	-£1,090
Education						
No formal qualification	£1,203	-0.076	DE dominates DEW	-£2,195	-£2,729	-£3,492
GCSE	£559	-0.056	DE dominates DEW	-£1,288	-£1,681	-£2,241
A-level	-£763	0.041	DEW dominates DE	£1,292	£1,578	£1,985
Post-secondary	£1,126	-0.013	DE dominates DEW	-£1,290	-£1,379	-£1,506
University degree	£1,211	0.029	41776	-£835	-£632	-£343
Higher degree	£167	-0.045	DE dominates DEW	-£758	-£1,076	-£1,531
Other	£142	-0.018	DE dominates DEW	-£370	-£493	-£668
Not stated	-£1,982	0.024	DEW dominates DE	£2,290	£2,456	£2,693
Duration of diabetes						
Diagnosed <1y ago	£1,504	-0.050	DE dominates DEW	-£2,156	-£2,507	-£3,008
Diagnosed 1-3y ago	£456	-0.025	DE dominates DEW	-£783	-£960	-£1,211

QALY, quality adjusted life year; iNMB, incremental net monetary benefit; DE, current standard care diabetes education (DESMOND programme); DEW, tailored diabetes education and behavioural weight management programme, delivered by WW; SE, standard error; CI, confidence interval. All incremental analyses show the values for

DEW minus the values for DE. Costs are in 2022/22 UK£. All analyses are as for the base case analysis but run on each sub-group. iNMBs are calculated at cost-effectiveness thresholds of £13,000, £20,000 and £30,000 per QALY gained.

ESM Table 23. Deterministic sensitivity analysis showing iNMB of different combinations of incremental costs and QALYs gained of DEW versus DE

		**Incremental costs**								
		-1.25 times (58)	-0.875 times (-29)	-0.5 times (-116)	-0.25 times (-174)	0 times (-232)	0.25 times (-290)	0.5 times (-347)	0.875 times (-434)	1.25 times (-521)
**QALYs gained**	-1.25 times (0)	-53	34	121	179	237	294	352	439	526
	-0.875 times (0)	-60	27	113	171	229	287	345	432	519
	-0.5 times (0)	-68	19	106	164	222	280	338	424	511
	-0.25 times (-0.001)	-73	14	101	159	217	275	333	420	506
	0 times (-0.001)	-78	9	96	154	212	270	328	415	502
	0.25 times (-0.001)	-82	4	91	149	207	265	323	410	497
	0.5 times (-0.001)	-87	0	86	144	202	260	318	405	492
	0.875 times (-0.002)	-95	-8	79	137	195	253	311	398	484
	1.25 times (-0.002)	-102	-15	72	130	188	245	303	390	477

QALY, quality adjusted life year; iNMB, incremental net monetary benefit; DE, current standard care diabetes education (DESMOND programme); DEW, tailored diabetes education and behavioural weight management programme, delivered by WW. Costs are in 2022/22 UK£. The heatmap shows the iNMB at a cost-effectiveness threshold of £20,000 per QALY gained for DEW minus the values for DE. All analyses are as for the base case analysis. The incremental costs and QALYs gained range from a 1.25 times decrement to a 1.25 times increment of the base case values shown on ESM Table 18. The absolute values of the incremental estimates are reported in parentheses. All possible combination are plotted and depicted on a chromatic scale ranging from red (not cost effective) to green (cost-effective).

ESM Figure 4. Cost-effectiveness acceptability curves showing the probability that DEW is cost-effective versus DE (y-axis) at different values of the maximum willingness to pay for a QALY (x-axis)

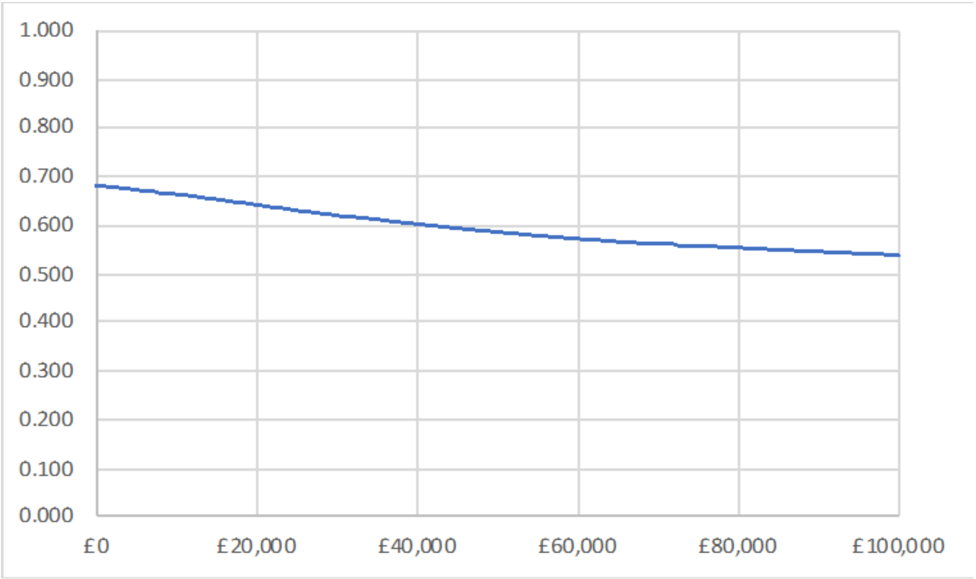

References

1. 4 Economic evaluation | NICE health technology evaluations: the manual | Guidance | NICE.
2. Personal Social Services Research Unit. Unit Costs of Health and Social Care [Internet]. [cited 2023 Nov 24]. Available from: https://www.pssru.ac.uk/project-pages/unit-costs/
3. NHS England » National Cost Collection for the NHS [Internet]. [cited 2024 Jan 9]. Available from: https://www.england.nhs.uk/costing-in-the-nhs/national-cost-collection/
4. BNF (British National Formulary) | NICE [Internet]. [cited 2024 Jan 9]. Available from: https://bnf.nice.org.uk/
5. dm+d browser [Internet]. [cited 2024 Jan 9]. Available from: https://dmd-browser.nhsbsa.nhs.uk/
6. WHOCC - ATC/DDD Index [Internet]. [cited 2024 Jan 9]. Available from: https://www.whocc.no/atc_ddd_index/
7. Herdman M, Gudex C, Lloyd A, Janssen M, Kind P, Parkin D, et al. Development and preliminary testing of the new five-level version of EQ-5D (EQ-5D-5L). Qual Life Res [Internet]. 2011 Dec [cited 2022 May 30];20(10):1727–36. Available from: https://pubmed.ncbi.nlm.nih.gov/21479777/
8. Hernández Alava M, Pudney S, Wailoo A. Estimating the Relationship Between EQ-5D-5L and EQ-5D-3L: Results from a UK Population Study. Pharmacoeconomics [Internet]. 2023 Feb 1 [cited 2024 Jan 9];41(2):199–207. Available from: https://link.springer.com/article/10.1007/s40273-022-01218-7
9. Little R, Rubin D. Statistical analysis with missing data. Vol. 333. Hoboken: Wiley; 2014.
10. Brand J, van Buuren S, le Cessie S, van den Hout W. Combining multiple imputation and bootstrap in the analysis of cost-effectiveness trial data. Stat Med [Internet]. 2019 Jan 30 [cited 2024 Jan 9];38(2):210–20. Available from: https://onlinelibrary.wiley.com/doi/full/10.1002/sim.7956
11. StataCorp. Stata Statistical Software: Release 14. College Station, TX: StataCorp LP;
12. Briggs AH, Gray AM. Methods in health service research: Handling uncertainty in economic evaluations of healthcare interventions. BMJ : British Medical Journal [Internet]. 1999 Sep 9 [cited 2024 Jan 9];319(7210):635. Available from: /pmc/articles/PMC1116497/
13. Nixon R, Wonderling D, Grieve R. How to estimate cost-effectiveness acceptability curves, confidence ellipses and incremental net benefits alongside randomised controlled trials [Internet]. [cited 2024 Jan 9]. Available from: https://www.mrc-bsu.cam.ac.uk/wp-content/uploads/nixon-wonderling-grieve-05.pdf

Electronic supplementary material (ESM) 4. Costs of delivering the DESMOND programme.

Description

DESMOND may be delivered either via face-to-face workshops or online. We calculated the costs of delivering both options and then calculated the weighted average of these based on the proportion of people attending each option.

For the face-to-face DESMOND programme the cost components are as follows:

- The cost of the license;
- The Clinical Commissioning Group (CCG)-wide costs, which need to be spread across all participants attending face-to-face workshops in the CCG; and,
- The cost per workshop, which needs to be spread across all participants attending the workshops.

We developed the costing in consultation with a commissioner of DESMOND based on local data for referrals, uptake of the service, and choice of delivery (face-to-face or online). Based on these discussions and using local data we assumed the following:

- We assumed that 58% of people attending the DESMOND programme used the face-to-face version, and 42% used the online version.
- On average each commissioner had 576 workshop participants in total,
- 6 people would attend each workshop.

For the online version of the DESMOND programme the cost components are as follows:

- The cost of the license;
- The per-participant onboarding costs for the online programme.

The costs for each of these components are in ESM Table 24. The mean costs per participant for each version of the programme, the proportion of participants attending each version of the programme, and the weighted average cost of the programme are in ESM Table 25.

ESM Table 24
Costs for each component of the DESMOND programme

	Total cost	Number of participants
**License cost (applies to both the face-to-face and online programmes**		
DESMOND licence	£10,000	1000
License cost per person	£10	
**CCG-wide costs for the face-to-face programme**		
Staff training payment to DESMOND	£2800	576
Staff training (Band 4 staff time £37 per hour)	£518	576
Staff training Band 7 staff time £66 per hour)	£924	576
Uniforms	£300	576
CCG-wide cost per person	£7.89	
**Workshop costs for the face-to-face programme**		
Staff cost (10 hours Band 4 staff time £37)	£370	6
Equipment and materials for 6 participants	£23.94	6
Venue hire (£300 per session)	£300	6
Band 4 Diabetes educator (Band 4, £37 per hour, 7.5 hours)	£277.50	6
Band 7 Diabetes educator (Band 4, £66 per hour, 7.5 hours)	£495.00	6
Mileage	£15.53	6
Workshop cost per person	£247.00	

**Online onboarding costs for the online programme**		
B4 staff	£2.47	1

ESM Table 25

Mean costs per participant of the DESMOND programme

**Mean cost per participant**	
Face-to-face programme (Licence + CCG-wide costs + Workshop costs)	£264.88
Online programme (Licence + Online onboarding costs)	£12.47
**Proportion of participants attending each version of the programme**	
Face-to-face programme	57.6%
Online programme	42.4%
**Weighted average cost per participant**	£157.86

Electronic supplementary material (ESM) 5: Modelled lifetime cost-effectiveness.

Baseline Population

Data

The model requires a complete population dataset that represents the baseline population that faced the weight management program in the Glucose Lowering through Weight Management (GLoW) trial [1]. The data must include demographic characteristics, anthropometric and metabolic measures, and a history of cardiovascular event outcomes for each individual in the model. To generate the appropriate cohort of patients to enter the model, the population is sampled using data from relevant sources. In order to represent the population facing the weight management program, a large portion of these requirements is informed by the baseline GLoW trial data, including the crucial demographic characteristics, weight and height and clinical risk factor measures such as HbA_{1c}. However, as the trial did not collect all data required within the Diabetes Treatment Model, the remaining required was taken from alternative sources, including the NICE Health Economic Model Report [2]. The NICE health economic model report's main data source was The Health Improvement Network (THIN) [3].

The GLoW trial had a sample of 577 participants, all recently diagnosed with type 2 diabetes. Summary statistics for the data extracted from the GLoW trial are reported in ESM Table 26. The inclusion criteria for the trial were adults (over 18 years) considered overweight or obese (BMI $\geq 25 kg/m^2$) who had been diagnosed with type 2 diabetes within the previous 36 months. Individuals that were using insulin or have previously had/are due to have bariatric surgery were excluded.

Sampling the Baseline Population

The baseline population used in the model was sampled from a multivariate normal distribution, informed by the data from GLoW and the reported population characteristics in NICE health economic model report and a review [1, 2, 4, 5]. To generate the necessary distributional information from the GLoW data, without reducing the sample size and losing representativeness, a data imputation process was used. Missing data from the GLoW trial dataset were imputed using multiple imputation methods from the mice package in R. Imputation through predictive mean methods filled any missing data, the majority of which was seen within clinical measures including cholesterol levels and blood pressure, and medication status. To account for the skewed distributions seen in the clinical risk factors in the GLoW data, including HbA_{1c}, low density lipoproteins, high density lipoproteins, triglycerides, systolic blood pressure, and weight, a log normal distribution was used when sampling.

The imputed GLoW data generated a correlation matrix, a matrix of standard deviations and a vector of means. These were combined with the data reported in the NICE report [5], which included the summary statistics and correlations from data from THIN, specifically in the 'initial therapy' subgroup. This subgroup provides a good source of data to replace that missing from GLoW as NICE defined those from THIN in initial therapy as having a median duration of diabetes of 1.5 years. The summary statistics from the THIN data, reported in NICE are in ESM Table 26. The THIN dataset reports the correlations between the additional data used and many of the overlapping demographic and clinical risk factors also reported in GLoW. This allowed for a collated correlation matrix to be formed. Any correlations not found through GLoW trial data or reported in NICE report were assumed to be zero. Using the collated distributional information, a variance-covariance matrix of all necessary variables was generated, and a baseline population sampled. The summary statistics of this sampled population and the source data are reported in ESM Table 26 .

ESM Table 26: summary statistics for baseline characteristics, sampling distribution and data sources

		Sample Population			Data Source		
**Variable**		**Mean/ %**	**SD**	**Assumed Distribution**	**Mean/ N**	**SD/%**	**Data Source**
Age (years)		59.2	12.1	Normal	59.8	12.6	[1]
Gender	Female (%)	52%			301	52.2%	[1]
	Male (%)	48%			276	47.8%	[1]
Ethnicity	Black (Afro-Caribbean)	3%			19	3.3%	[1]
	Asian	4%			22	3.8%	[1]
	Other (inc. Mixed)	93%			536	92.9%	[1]
IMD Quintile	1st Quintile (Most Deprived)	16%			79	15.5%	[1]
	2nd Quintile	16%			81	15.9%	[1]
	3rd Quintile	24%			124	24.3%	[1]
	4th Quintile	22%			113	22.2%	[1]
	5th Quintile (Least Deprived)	22%			113	22.2%	[1]
Height (cm)		168.2	10.1	Normal	168.25	10.09	[1]
Weight (kg)		98.9	19.9	Log-Normal	97.90	20.44	[1]
BMI		35.1	6.4	Normal	34.62	6.78	[1]
	Underweight (<18kg/m2)	0%			0	0	[1]
	Healthy Weight	6%			9	1.6%	[1]
	Overweight (>25kg/m2)	94%			566		[1]
Diabetes Duration	Less than 1 Year	55%			316	54.8%	[1]
	Between 1-3 Years	45%			261	45.2%	[1]
HbA_{1c} (mmol/mol)					53.89	13.57	[1]
HbA_{1c} (%)		7.2	1.1	Log-Normal	7.08	1.24	[1]
Cholesterol (mmol/l)		4.8	1.2	Log-Normal	4.74	1.07	[1]
HDL-C (mmol/l)		1.2	0.3	Log-Normal	1.25	0.48	[1]
LDL-C (mmol/l)		2.6	0.9	Log-Normal	2.53	0.85	[1]
Triglycerides (mmol/l)		2.3	1.1	Log-Normal	2.23	1.16	[1]
SBP (mmHg)		134.6	18.1	Log-Normal	134.53	17.30	[1]
HbA_{1c} at Diagnosis (%)		7.0	1.6	Log-Normal	ESM Table 7	2.0	[1, 5]
HDL-C at Diagnosis (mmol/l)		1.2	0.3	Log-Normal	ESM Table 7	0.3	[1, 5]
LDL-C at Diagnosis (mmol/l)		2.6	0.9	Log-Normal			Sampled Baseline LDL-C
SBP at Diagnosis (mmHg)		136.3	16.5	Log-Normal	ESM Table 7	17.1	[1, 5]
BMI at Diagnosis		35.1	6.4	Normal			Sampled Baseline BMI
eGFR		73.7	14.3	Normal	73.7	14.26	Mean [2] SD is same proportion of Mean for eGFR [4]
Heart Rate		72.0	12.0	Normal	72	12	[4]
Haemoglobin		14.5	0.0	Fixed	14.5		[2]
WBC		7.6	0.0	Fixed	7.58		[2]
Smoking Status	Currently Smoking	11%			54	10.4%	[1]
	Not Currently Smoking	89%			465	89.6%	[1]
Smoking Status at diagnosis	Currently Smoking	11%			54	10.4%	[1]
	Not Currently Smoking	89%			465	89.6%	[1]
Antihypertensive Medication	Currently prescribed	55%			195	55.4%	[1]
	Not currently prescribed	45%			242	44.6%	[1]
Statins	Currently prescribed	56%			193	55.8%	[1]
	Not currently prescribed	44%			244	44.2%	[1]
Metformin Monotherapy	Currently prescribed	100%					Assumption
	Not currently prescribed	0%					
**Event History**							
Atrial Fibrillation	Yes	0.8%			0.81%		[5]
	No	99.2%					[5]
Peripheral Vascular Disease	Yes	0.5%			0.51%		[5]
	No	99.5%					[5]
Ischemic Heart Disease	Yes	2.7%			2.7%		[5]
	No	97.3%					[5]

Congestive Heart Failure	Yes	0.5%			0.5%		[5]
	No	99.5%					[5]
Amputation	Yes	0.1%			0.1%		[5]
	No	99.9%					[5]
Renal Failure	Yes	0.2%			0.2%		[5]
	No	99.8%					[5]
Stroke	Yes	0.5%			0.5%		[5]
	No	99.5%					[5]
Myocardial Infarction	Yes	0.8%			0.8%		[5]
	No	99.2%					[5]
Blindness	Yes	0.4%			0.4%		[5]
	No	99.6%					[5]
Ulcer	Yes	0.2%			0.2%		[5]
	No	99.8%					[5]
Micro-Albuminuria	Yes	10.4%			10.30 %		[5]
	No	89.6%					[5]

HDL-C, HDL-cholesterol; LDL-C, LDL-cholesterol;

The model requires data indicating the patient risk factors measured at the point they were diagnosed. However, this data was not available from the GLOW trial. To generate a measure of risk factors at diagnosis for the GLoW population, the difference between mean values of HbA_{1c}, total cholesterol, HDL-C, SBP at diagnosis and at baseline reported in NICE [5] were used to calculate the mean value at diagnosis by taking this difference seen in THIN from the baseline values of mean risk factors in the GLoW trial. The baseline measures of risk factors were then sampled from these mean values, assuming the standard deviation of baseline risk factors reported within NICE. The mean LDL-C measurements at the point of diagnosis in the THIN data were not reported in the NICE28 report, therefore the sampled baseline values of LDL-C were used for the value at diagnosis. Smoking status at diagnosis was set to the value at baseline, sampled from the GLoW trial data.

ESM Table 27: Derivation of metabolic risk at diagnosis using data observed data from GLOW and differences reported in THIN

Risk factor	Difference in Means from THIN [5]	Baseline Mean - GLoW	Estimated mean score at diagnosis
Total cholesterol (mmol/l)	-0.31	4.96	5.27
HD lipoprotein (mmol/l)	0.01	1.18	1.17
Sys. blood pressure (mmHg)	-2.1	137.5	139.6
HbA_{1c} (%)	0.2	8.40	8.20

The UKPDS 90 risk factor trajectories have several limitations. The nature of the equations and the age of the data used to generate them reduces random variation between individual's trajectory and causes their direction to be highly dependent on the current and earlier recorded value of the risk factor relative to the average seen in UKPDS population. To ensure the trajectories imposed are realistic, we took careful consideration of the risk factor values sampled at the point of diagnosis. THIN informed HbA_{1c} and systolic blood pressure suitably such that the sampled values at diagnosis, alongside the baseline values from the GLoW trial, produced a trajectory that followed a similar pattern to that observed in UKPDS. However, the value at diagnosis sampled for LDL-C and HDL-C produce a trajectory that changed dramatically within the first 5 years, before levelling off. To ensure the trajectory estimated followed a path expected by UKPDS, it was deemed appropriate to select a value at diagnosis of these risk factors that would produce a trajectory that was consistent with trends in UKPDS adjusting for their different baseline values. LDL-C at diagnosis was given as the individuals sampled baseline LDL-C minus 1 mmol/l, while HDL-C at diagnosis was given as the individuals sampled baseline HDL-C plus 0.1 mmol/l. The trajectories estimated from our sampled population are shown in Section 0: Validation.

Where HbA_{1c} required converting from mmol/mol unit to percentage, the following formula was used [6].

$$\mathbf{A1C}(\%) = (\mathbf{A1C}(\text{mmol/mol}) / 10.929) + 2.15$$

Exclusion Criteria

To avoid the inclusion of unrealistic individual characteristics that can be sampled using multivariate normal distributions, certain limitations were set. The inclusion criteria defined participants as adults, and thus the sampled population were limited to only those above 18 years old. Similarly, while there were no formal exclusion criteria based on old age, to avoid sampling individuals much older than could realistically participate in the GLoW trial, only

sampled individuals aged at or below the oldest participant in the trial (89 years) sample were included. To ensure BMI and HbA_{1c} was sampled to reflect the likely population that would participate in the GLoW trial, the lowest value of these recorded in the trial data was used as a lower boundary, at 20.9kg/m^2 and 5.08% (32mmol/mol) respectively. Despite the inclusion criteria defining BMI as being over 25kg/m^2, the minimum recorded BMI within the trial was 20.9kg/m^2, therefore this was the chosen boundary.

GP Attendance in General Population

GP visit frequency was simulated in the dataset to estimate healthcare utilisation for the general population and the costs associated with these GP appointments, in order to complete a comprehensive cost-effective analysis in the model. A statistical model of GP attendance, conditional on characteristics that are associated, such as age, ethnicity, and comorbidities, was developed. If those with life limiting health concerns visit the GP more, then a change in the presence of these conditions will change the number attending GP appointments and thus creating an overall change in primary-care costs.

A negative binomial model was used to generate count data and a skewed distribution was observed in the dataset.

$$\mu_i = \exp{(x_i\beta)}$$

The dispersion parameter of the Negative Binomial distribution v_i was sampled from a gamma distribution with mean 1 and variance α based on estimates reported in ESM Table 28. The frequency was estimated from the Poisson function.

$$p(Y = y|y > 0, x) = \frac{(v_i\mu_i)^y e^{-(v_i\mu_i)}}{y!}$$

We used data from the Health Survey for England (2019), which collected a patient reported variable on the number times they attended a GP in the last year. It also collected

demographic and health related information, including whether they had diabetes or not. This would allow the GP attendance model to represent the relevant population within the Diabetes Treatment Model. While explicit variables indicating comorbidities were not included, variables indicating which related medications they are taken is reported. Whether the patient has a prescription for antidepressants, lipid lowering medication or anti-hypertensive medication is thought to have a relation with GP attendance, especially considering the policies relating to regular medication reviews. A variable indicating whether an individual has a prescription for these medications is included in the model. Finally, to account for any comorbidities, a variable indicating whether they have a life limiting illness was included. The population included were adults above 20 years old. The characteristics of the study population are reported in ESM Table 28.

ESM Table 28: Characteristics of HSE 2019

**Variable**	**Observations**	**Mean**	**SD**
GP Visits	7,746	2.96	2.99
Age (20+)	7,855	52.52	17.69
Male	7,855	0.46	0.50
Black	7,826	0.03	0.18
Asian	7,826	0.10	0.30
Ethnic Minority	7,826	0.17	0.37
BMI	6,470	26.24	6.60
Diabetes	7,848	0.08	0.27
Life Limiting Illness	7,847	0.24	0.43
Lipid Lowering Medication	4,774	0.15	0.36
Anti-Hypertensive Medication	4,768	0.24	0.43
Antidepressant	4,774	0.10	0.30

The coefficients of the Negative Binomial model, described in ESM Table 29, were used to calculate the first parameter of the Negative Binomial distribution μ_i based on an individual's characteristics. The dispersion parameter sampled from the gamma distribution using the alpha reported in ESM Table 29.Three regression specifications are reported in ESM Table 29 Model 1 regresses GP visits against age, sex, ethnicity, BMI, whether they have diabetes

and whether they have a life limiting illness. Model 2 regresses GP visits against all parameters in model 1, but with the additional variable indicating if they are prescribed antidepressant medications. Finally Model 3 included whether they are prescribed CVD medication (lipid lowering and anti-hypertensive medication) on addition to the coefficients in Model 2.

ESM Table 29: Negative Binomial model coefficients to predict GP attendance using the HSE 2019

	**Model 1**		**Model 2**		**Model 3**	
	Mean	SE	Mean	SE	Mean	SE
Age	0.00263***	(0.000725)	0.00195**	(0.000880)	-0.00221**	(0.000989)
Male	-0.211***	(0.0246)	-0.177***	(0.0295)	-0.220***	(0.0297)
Black	0.135*	(0.0726)	0.296***	(0.0906)	0.303***	(0.0902)
Asian	0.211***	(0.0438)	0.247***	(0.0562)	0.242***	(0.0557)
BMI	0.0105***	(0.00207)	0.00866***	(0.00246)	0.00548**	(0.00247)
Diabetes	0.282***	(0.0438)	0.238***	(0.0517)	0.112**	(0.0533)
Life Limiting Illness	0.700***	(0.0263)	0.587***	(0.0320)	0.553***	(0.0319)
Antidepressants			0.406***	(0.0417)	0.407***	(0.0413)
Lipid lowering Medication					0.207***	(0.0424)
Anti-Hypertensive Medication					0.224***	(0.0392)
Constant	0.422***	(0.0704)	0.480***	(0.0844)	0.727***	(0.0883)
Alpha	0.547	(0.0180)	0.494	(0.0206)	0.476	(0.0202)
LnAlpha	-0.603***	(0.0330)	-0.705***	(0.0417)	-0.741***	(0.0424)
N	6,378		4,215		4,209	
Pseudo R^2	0.0357		0.0392		0.0434	
Standard errors in parentheses (*** p<0.01, ** p<0.05, * p<0.1)						

The average number of GP visits was approximately 3 times in the year. The number of GP visits was statistically significantly related with all variables included. Individuals that are Black or Asian are more likely to attend the GP, while males are less likely. Those with a higher BMI, a life limiting illnesses or are taking CVD medication and antidepressants go to the GP more often. The large positive relationships between GP attendance and indicators of

ill health justify their inclusion. An unexpected negative relationship is seen in age when including CVD medication variables. This could be explained by the strong positive correlation between age and taking CVD related medication.

Model 3 was chosen to model GP utilisation due to the high statistical significance of all variables and the greater Pseudo R^2 value. The variable covariance matrix from Model 3 is reported in ESM Table 30.

ESM Table 30: Variance-covariance matrix for GP Attendance, Model 3

	Age	Male	Black	Asian	BMI	Diabetes	Life Limiting Illness	Anti-depressants	Lipid Lowering Med	Anti-Hypertensive Med	Constant	LnAlpha
Age	0.0000											
Male	-0.0000	0.0009										
Black	0.0000	0.0001	0.0081									
Asian	0.0000	0.0000	0.0003	0.0031								
BMI	0.0000	-0.0000	-0.0000	0.0000	0.0000							
Diabetes	-0.0000	-0.0000	-0.0002	-0.0001	-0.0000	0.0028						
Life Limiting Illness	-0.0000	0.0000	-0.0000	0.0000	-0.0000	-0.0001	0.0010					
Antidepressants	0.0000	0.0001	0.0002	0.0002	-0.0000	-0.0001	-0.0003	0.0017				
Lipid lowering Med	-0.0000	-0.0002	0.0001	-0.0000	-0.0000	-0.0005	-0.0000	-0.0001	0.0018			
Anti-Hypertensive Med	-0.0000	-0.0000	-0.0000	-0.0000	-0.0000	-0.0002	-0.0001	0.0000	-0.0005	0.0015		
Constant	-0.0001	-0.0003	-0.0005	-0.0009	-0.0002	0.0004	0.0000	-0.0001	0.0006	0.0008	0.0078	
**LnAlpha**	0.0000	-0.0000	-0.0000	0.0000	0.0000	0.0000	0.0000	0.0000	0.0000	0.0000	-0.0000	0.0018

Longitudinal Trajectories of Natural History metabolic Risk Factors

To simulate the change in metabolic risk factors within the population over time, the UKPDS90 risk factor trajectory equations are included in the model [7]. UKPDS90 used data from the UKPDS trial, which evaluated different management regimes for individuals who had recently been diagnosed with type 2 diabetes, representing a similar position of diagnosis as that for the participants of the GLoW trial. Starting in 1977, the trail followed individuals for 20 years (1977-1997), collecting data relating to metabolic risk factors. After, all surviving participants entered a post-trial monitoring period for an additional 10 years (1997-2007). The UKPDS outcomes model was a simulation model for predicting diabetes related complications based on the patients' demographics, health history and clinical risk factors. UKPDS 90 added to this by producing models to estimate the trajectories of these clinical risk factors.

Dynamic linear models were developed to examine the trajectory of the continuous risk factors (HbA_{1c}, SBP, LDL-C, HDL-C, BMI, Heart rate, WBC, Haemoglobin). Using panel

data, they estimated the time path of these metabolic factors by fitting a random effects model. Multivariate parametric proportional hazards survival models were used to estimate the risk of developing PVD, micro or macro-albuminuria, atrial fibrillation or having an eGFR below 60 ml/min/173m2. Two additional multivariable random effects Tobit autoregressive models (order 1) were used to find the time path for a continuous value of eGFR. Finally, a random effects logistic autoregressive model was used to model smoker status. These growth factors are conditional on sex, ethnicity, the first recorded value of the risk factor, the most recent recorded value of the risk factor and the amount of time since diagnosis of T2D [7].

These trajectories, estimated by UKPDS90, were included in the treatment model to simulate how individuals' risk factors change overtime and therefore more accurately predict the risk of CVD event outcomes. In this model, these clinical risk factors are not considered to be co-dependent on each other, only on previous values of themselves.

HbA_{1c}

An individual's HbA_{1c} in the treatment model follows an estimated trajectory, conditional on their demographics and previous HbA_{1c} measurements. HbA_{1c} is estimated to increase with previous measures of HbA_{1c} and the length of time since diagnosis. The time path does not consider the impact of glucose management medications. The regression parameters used are reported in ESM Table 31.

ESM Table 31: UKPDS 90 parameters for HbA_{1c} (%) trajectory

Measurement Frequency	Annual	
Units	%	
No. of Observations	54,714	
No. of Individuals	4906	
Functional Form	Panel	
R^2 Overall	0.603	
**Risk factor**	**Estimate of Coefficient**	**SE**

Constant	1.419	(0.041)
Female	0.054	(0.012)
African Caribbean	0.066	(0.026)
Asian Indian	0.046	(0.020)
Value of HbA_{1c} in Previous Year	0.724	(0.005)
ln(year since diagnosis)	0.141	(0.007)
First recorded value of HbA_{1c}	0.081	(0.007)

BMI

The UKPDS90 estimated trajectory will determine the individuals BMI time path in the model. An individual's BMI today is highly informative for their future BMI. Although, BMI falls with time after diagnosis of T2D. The regression parameters used are reported in ESM Table 32.

ESM Table 32: UKPDS 90 parameters for BMI (kg/m^2) trajectory

Measurement Frequency	Annual	
Units	kg/m^2	
No. of Observations	59,219	
No. of Individuals	4951	
Functional Form	Panel	
R^2 Overall	0.965	
**Risk factor**	**Estimate of Coefficient**	**SE**
Constant	0.830	(0.039)
Female	0.045	(0.011)
African Caribbean	-0.094	(0.016)
Asian Indian	-0.087	(0.014)
Value of BMI in Previous Year	0.952	(0.003)
ln(year since diagnosis)	-0.165	(0.006)
First recorded value of BMI	0.034	(0.003)

SBP

UKPDS90 estimated SBP to increase with the number of years since diagnosis and by the previous year's level. The regression parameters used to estimate SBP are reported in ESM Table 33.

ESM Table 33: UKPDS90 parameters for Systolic Blood Pressure (mmHg) trajectory

Measurement Frequency	Annual	
Units	mmHg	
No. of Observations	58,549	
No. of Individuals	4933	
Functional Form	Panel	
R^2 Overall	0.570	
**Risk factor**	**Estimate of Coefficient**	**SE**
Constant	29.007	(0.597)
Female	0.684	(0.142)

African Caribbean		
Asian Indian	-1.393	(0.224)
Value of SBP in Previous Year	0.669	(0.005)
ln(year since diagnosis)	0.570	(0.064)
First recorded value of SBP	0.118	(0.005)

High-density lipoprotein cholesterol (HDL-C)

The UKPDS90 growth trajectories of HDL-C are conditional on sex, being African Caribbean, and previous measures of HDL-C. The regression parameters used to estimate HDL-C are reported in ESM Table 34.

ESM Table 34: UKPDS90 parameters for High-density lipoprotein (HDL-C) (mmol/l) trajectory

Measurement Frequency	Annual	
Units	mmol/l	
No. of Observations	41,805	
No. of Individuals	4900	
Functional Form	Panel	
R^2 Overall	0.565	
**Risk factor**	**Estimate of Coefficient**	**SE**
Constant	0.170	(0.009)
Female	0.043	(0.003)
African Caribbean	0.051	(0.006)
Asian Indian		
Value of HDL in Previous Year	0.603	(0.015)
ln(year since diagnosis)		
First recorded value of HDL-C	0.220	(0.009)

Low-density lipoprotein cholesterol (LDL-C)

UKPDS90 estimates LDL-C to be lower in ethnic minorities but higher in females. It is estimated to increase with previous measures of LDL-C but decrease with time since diagnosis of T2D. The regression parameters used to estimate LDL-C are reported in ESM Table 35.

ESM Table 35: UKPDS90 parameters for High-density lipoprotein (HDL-C) (mmol/l) trajectory

Measurement Frequency	Annual	
Units	mmol/l	
No. of Observations	41,635	
No. of Individuals	4901	
Functional Form	Panel	
R^2 Overall	0.585	
**Risk factor**	**Estimate of Coefficient**	**SE**
Constant	0.763	(0.020)
Female	0.065	(0.009)
African Caribbean	-0.050	(0.016)
Asian Indian	-0.074	(0.014)
Value of LDL-C in Previous Year	0.578	(0.007)

ln(year since diagnosis)	-0.042	(0.004)
First recorded value of LDL-C	0.210	(0.007)

Other

Other – Dynamic Modelling

The estimated relationships in the remaining risk factors determined through dynamic modelling are described in ESM Table 36. While these risk factors are not as central to this investigation, UKPDS-OM2 found them to have an important impact on potential health outcomes. Therefore, it is important to simulate their trajectories within the model.

ESM Table 36: Other UKPDS 90 parameters for continuous trajectories

**Risk factor (Y)**	**HEART R**		**WBC**		**HAEM**		**EGFR<60[b] (continuous)**		**EGFR≥60[c] (continuous)**	
Measurement frequency	Every 3 years		Every 3 years		Every 3 years		Annual		Annual	
Units	bpm		$1x10^6$ ml		g/dL					
No. of observations	7973		8526		13,411					
No. of individuals	3891		4042		4655		4279		4279	
Functional form	Panel		Panel		Panel		Tobit		Tobit	
R^2 overall	0.321		0.457		0.476					
**Parameters**	**Coefficient**	**SE**	**Coefficient**	**SE**	**Coefficient**	**SE**	**Coefficient**	**SE**	**Coefficient**	**SE**
Constant	31.231	(1.468)	1.446	(0.242)	5.040	(0.295)	26.102	(0.976)	23.970	(0.783)
Γ/σ							9.452		12.575	
Female	1.006	(0.316)	0.087	(0.042)	-0.349	(0.036)	-2.409	(0.271)	-2.985	(0.262)
African Caribbean			-0.331	(0.066)	-0.185	(0.045)	2.162	(0.594)	3.419	(0.474)
Asian-Indian							1.229	(0.506)	2.404	(0.421)
Value of Y in previous year*	0.327	(0.021)	0.460	(0.116)			0.567	(0.010)	0.406	(0.006)
ln (year since diagnosis)	0.918	(0.469)	0.167	(0.050)	-0.326	(0.028)	-3.280	(0.185)	-3.013	(0.128)
First recorded value of Y	0.272	(0.021)	0.292	(0.100)	0.692	(0.020)	0.138	(0.009)	0.297	(0.008)

[b] estimating annual eGFR values conditional having transitioned to eGFR lower than 60 ml/min/173m^2; [c] estimating annual eGFR values conditional on not having transitioned to eGFR lower than 60 ml/min/173m^2

Other - multivariable parametric proportional hazards survival models

The remaining risk factors are binary and found using multivariable parametric proportional hazards survival models. These allow estimation of the probability the individual will be diagnosed with albuminuria, PVD, atrial fibrillation, or an eGFR of less than 60. UKPDS 90 also produces a model describing the probability of being a smoker. The predicted presence of these factors will influence probability of event outcomes. The estimated regression parameters from UKPDS90 for the risk factor hazard models are reported in ESM Table 37.

ESM Table 37: Other UKPDS 90 parameters for binary risk factors

**Risk factor/event**	**MIC ALB**	**PVD**	**AT FIB**	**SMOKER**	**EGFR<60[a] (binary)**
Measurement frequency	Annual	Every 3 years	Every 3 years	Every 3 years	Annual
Patient-years/no. of observations	37,853	38,831	62,369	12,961	39,958
Number of individuals	4594	4591	4987	4194	4621
Number of events	866	685	85	-	1107
Functional form	Weibull	Weibull	Exponential	Logistic	Weibull

Parameters	**Coefficient**	**SE**	**Coefficient**	**SE**	**Coefficient**	**SE**	**Coefficient**	**SE**	**Coefficient**	**SE**
Constant	-9.047	(0.443)	-12.271	(0.058)	-13.313	(1.148)	0.016	(0.529)	-11.784	(0.425)
Γ/σ	1.138	(0.049)	1.515	(0.058)					1.871	(0.052)
Female	-0.463	(0.077)					-0.297	(0.146)	0.745	(0.067)
African Caribbean									-0.974	(0.169)
Asian-Indian									-0.302	(0.142)
Age at diagnosis	0.012	(0.004)	0.057	(0.006)	0.089	(0.016)	-0.050	(0.008)	0.080	(0.005)
Smoker in previous year	0.329	(0.076)	0.865	(0.083)			2.018	(0.175)		
Smoker at first recorded value							5.535	(0.436)		
SBP in previous year (/10)	0.186	(0.018)	0.098	(0.022)					0.075	(0.017)
HbA_{1c} in previous year	0.165	(0.028)	0.095	(0.022)						
BMI in previous year	0.028	(0.006)	0.023	(0.007)	0.065	(0.017)			0.014	(0.006)
HDL-C in previous year (x10)	-0.030	(0.013)							-0.028	(0.011)
LDL in previous year (x10)			0.025	(0.003)					0.008	(0.003)
ln (year since diagnosis)							-1.574	(0.169)		

[a] estimating probability of transitioning to eGFR lower than 60 ml/min/1.73m^2;

Ethnicity appears less related to these binary risk factors as it is only included in the eGFR model, despite it relating to nearly all continuous clinical measures listed previously. On the other hand, being older at the point of type 2 diabetes diagnosis increases the probability of being diagnosed with all these health concerns.

The parametric survival models were used to generate estimates of the cumulative hazard in the current and next period. From which the probability of getting a diagnosis of these health outcomes was estimated.

$$p(outcome) = 1 - \exp\,(H(t) - H(t+1))$$

The functional form for these risk factor models included exponential and Weibull, and logistic for the smoking status.

The exponential model assumes a baseline hazard λ, which can be calculated from the model coefficients reported in ESM Table 37 and the individual characteristics for $\boldsymbol{X}$.

$$\lambda = exp\left(\beta_0 + \boldsymbol{X\beta}\right)$$

The Weibull model assumes a baseline hazard:

$$h(t) = \rho t^{\rho-1} \exp\,(\lambda)$$

where λ is also conditional on the coefficients and individual characteristics at time t.

The logistic model for ulcer is described below.

$$\Pr(y = 1|\mathbf{X}) = \frac{\exp\,(\mathbf{X\beta})}{1 + \exp\,(\mathbf{X\beta}))}$$

Comorbid Outcomes and Mortality

In every model cycle, individuals within the model are evaluated to determine whether they have a clinical event, including mortality, within that cycle period. In each case the simulation estimates the probability that an individual has the event and uses a random number draw to determine whether the event occurred.

The UKPDS Outcomes Model (version 2) contains 13 risk equations that estimate the probability of an individual facing a range of macrovascular and microvascular complications. The UKPDS-OM2 was generated using UKPDS trial data on individuals recently diagnosed with T2D. The UKPDS outcomes models are ‘a patient-level epidemiological model for a target population of adults aged 30 and over with any duration of diabetes’[8]. Parametric proportional hazards models that predict the absolute risk of diabetes complications were derived using data from the patients from the 20-year UKPDS trial and any survivors that entered the 10-year post-trial monitoring period. The probabilities of complications were estimated conditional on a range of individual characteristics, demographics and clinical risk factors but also on current and previous CVD events.

Due to the population of recently diagnosed Type 2 diabetics used in UKPDS trial, the UKPDS82 risk equations have similarities to the population used in this analysis of the GLOW trial. These 13 equations are used in the Diabetes Treatment Model to simulate the CVD event outcomes for individuals facing T2D, allowing analysis of the effectiveness and cost-effectiveness of the weight management program within the Type 2 diabetic population included in this analysis. The risk equations predict the probability of death, a number of macrovascular outcomes (Myocardial infarction (MI), Stroke, Congestive Heart Failure (CHF), Ischaemic heart Disease (IHD)) as well as a number of microvascular outcomes (amputation, ulcer, blindness and renal failure). For some of the complications, multiple models were produced to predict secondary events. The covariates considered across these

equations included age at diagnosis of T2D, sex, ethnicity, whether they smoked, BMI, HbA_{1c}, SBP, HDL-C, LDL, heart rate, eGFR, WBC, Haemoglobin, whether they have Micro/macro albuminuria, atrial fibrillation or peripheral vascular disease, and their history of cardiovascular events.

The parametric survival models were used to generate estimates of the cumulative hazard in the current and previous period. From which the probability of these events occurring was estimated.

$$p(Death) = 1 - \exp\left(H(t) - H(t-1)\right)$$

The functional form for the macro- and microvascular models included exponential, Weibull and logistic.

Macrovascular Complications

The covariates of the UKPDS-OM2 models estimating the probability of a macrovascular event occurring are reported in ESM Table 38.

ESM Table 38: UKPDS 82 parameters for macrovascular events

	Eq.1		Eq.2		Eq.3		Eq.4		Eq.5		Eq.6		Eq.7	
	1st CHF		1st IHD		1st MI Male		1st MI Female		2nd MI		1st Stroke		2nd Stroke	
Patient-Years	77941		75163		43032		32093		4799		77332		2368	
Number of Patients	4977		4967		2910		2042		1012		4981		506	
Number of Events	334		721		619		334		169		490		78	
Functional Form	Weibull		Weibull		Exponential		Weibull		Exponential		Weibull		Weibull	
	Mean	SE	Mean	SE	Mean	SE	Mean	SE	Mean	SE	Mean	SE	Mean	SE
Lambda	-12.332	0.859	-6.709	0.503	-8.791	0.486	-8.708	0.844	-4.179	0.262	-13.053	0.722	-9.431	1.569
Rho	1.514	0.096	1.276	0.059			1.376	0.097			1.466	0.081	1.956	0.291
Age Diagnosis	0.068	0.008	0.016	0.005	0.045	0.006	0.041	0.008			0.066	0.007	0.046	0.018
Female			-0.532	0.085							-0.420	0.098		
Afro					-0.83	0.237	-1.684	0.506						
Indian					0.279	0.126								
Smoking Status					0.277	0.091	0.344	0.138			0.331	0.111	0.656	0.263
BMI	0.072	0.008												
HbA_{1c}					0.108	0.023	0.078	0.030			0.092	0.026		
SBP			0.058	0.019	0.046	0.022	0.056	0.027			0.170	0.022		
HDL-C			-0.065	0.014	-0.049	0.016								
LDL	0.012	0.005	0.023	0.003	0.023	0.004			0.021	0.007	0.016	0.004		
LDL>35							0.035	0.007						
eGFR			-0.053	0.023										
eGFR<60	-0.22	0.065					-0.280	0.062			-0.190	0.056		
MMALB	0.771	0.116			0.203	0.094	0.277	0.129	0.344	0.162	0.420	0.101	0.537	0.228
WBC					0.026	0.013	0.070	0.023			0.040	0.012		

ATFib	1.562	0.245									1.476	0.201	
PVD	0.479	0.136	0.486	0.101	0.34	0.111	0.469	0.132					
IHD History					0.846	0.117	0.876	0.163			0.481	0.144	
CHF History			0.824	0.168	0.814	0.195	0.853	0.200					
Stroke History					0.448	0.173							
Amputation History	0.658	0.334	0.526	0.266	0.743	0.241					1.090	0.240	
Ulcer History	0.654	0.291											

The functional form of the exponential model and the Weibull model are explicitly defined previously.

Relative Risk of MI and Stroke

The baseline population is sampled with a proportion receiving treatment for hypertension and high cholesterol. An additional treatment pathway, based on NICE guidelines for treatment of hypertension, simulates whether the population transitions onto antihypertensives. It is assumed that once on antihypertensives or statins, the individual remains on it until they die. These treatments are designed to reduce overall incidence of cardiovascular disease through a known treatment pathway of medications reducing blood pressure and cholesterol. To capture this within the model, any individual receives statins or antihypertensives receives a reduced probability of events, implemented through a relative risk that is informed through two meta-analyses. Once the probability of an MI, stroke of CHF is estimated through UKPDS OM2 equations, a relative risk is applied, reducing this probability by a set proportion. The relative risks are reported in ESM Table 39.

ESM Table 39: Relative risk reduction of major events for individuals receiving statins and anti-hypertensives

				95% Confidence Interval	
		Source	Mean	Lower	Upper
Statins	MI	[9]	0.62	0.53	0.72
	CHF		0.84	0.71	1.02
	Stroke		0.83	0.75	0.92
	Mortality		0.89	0.85	0.93
Antihypertensives	MI	[10]	0.88	0.79	0.97
	CHF		0.81	0.7	0.94

	Stroke		0.91	0.83	0.94
	Mortality		0.86	0.79	0.93

Microvascular Complications

We used the UKPDS Outcomes model to estimate the occurrence of major events relating to microvascular complications, including renal failure, amputation, foot ulcer, and blindness [8]. The entire simulated population is assumed to be at risk of these complications due to their diabetes diagnosis.

The parametric survival models were used to generate estimates of the cumulative hazard in the current and previous period. From which the probability of organ damage being diagnosed was estimated.

$$p(Death) = 1 - \exp\left(H(t) - H(t-1)\right)$$

The functional form for the microvascular models included exponential and Weibull and logistic.

Retinopathy

To investigate diabetes related retinopathy outcomes, we include the UKPDS-OM2 survival model for blindness, which takes an exponential functional form.

ESM Table 40: Parameters of the UKPDS2 Exponential Blindness survival model (Eq. 8)

	Mean coefficient	Standard error
Lambda	-11.607	0.759
Age at diagnosis	0.047	0.009
HbA_{1c}	0.171	0.032
Heart rate	0.080	0.039
SBP	0.068	0.032
White Blood Count	0.052	0.019
CHF History	0.841	0.287
IHD History	0.0610	0.208
SBP Systolic Blood Pressure; CHF Congestive Heart Failure; IHD Ischaemic Heart Disease		

Neuropathy

The UKPDS includes four statistical models to predict foot ulcers, amputation with no prior ulcer, amputation with prior ulcer and a second amputation [8]. The parameters of these models are reported in ESM Table 41.

ESM Table 41: Parameters of the UKPDS2 Exponential model for Ulcer, Weibull model for first amputation with no prior ulcer and exponential model for 1st amputation with prior ulcer

	Ulcer (Eq.9)		1st Amputation no prior ulcer (Eq.10)		1st Amputation prior ulcer (Eq.11)		2nd Amputation (Eq.12)	
	Logistic		Weibull		Exponential		Exponential	
	Mean	Standard error	Mean	Standard error	Mean	Standard error	Mean	Standard error
Lambda	-11.295	1.130	-14.844	1.205	-0.881	1.39	-3.455	0.565
Rho			2.067	0.193				
Age at diagnosis	0.043	0.014	0.023	0.011	-0.065	0.027		
Female	-0.962	0.255	-0.0445	0.189				
Atrial fibrillation			1.088	0.398				
BMI	0.053	0.019						
HbA_{1c}	0.160	0.056	0.248	0.042			0.127	0.06
HDL-C			-0.059	0.032				
Heart rate			0.098	0.050				
MMALB			0.602	0.180				
PVD	0.968	0.258	1.010	0.189	1.769	0.449		
SBP			0.086	0.043				
WBC			0.040	0.017				
Stroke History			1.299	0.245				

Nephropathy

To consider diabetic nephropathy, we include the UKPD model for renal failure.

The parameters of the renal failure risk model are reported in ESM Table 42.

ESM Table 42: Parameters of the UKPDS2 Exponential renal failure survival model

Parameters of the UKPDS2 Exponential renal failure survival model (Eq.13)		
	Mean coefficient	Standard error
Lambda	.549	1.48
Age at diagnosis	-0.029	0.013
Afro	0.686	0.324
Female	-0.869	0.224
BMI	-0.054	0.02
eGFR < 60	-1.031	0.085
eGFR > 60	-0.487	0.136
Haemoglobin	-0.268	0.061
LDL	0.027	0.007
MMALB	1.373	0.236
SBP	0.085	0.047
White Blood Count	0.029	0.013
Amputation History	1.108	0.0337
Blindness History	0.732	0.29
SBP Systolic Blood Pressure; CHF Congestive Heart Failure; IHD Ischaemic Heart Disease		

In the simulation, the estimated risk of these equations for each individual, in each model cycle, are calculated in the order the equations are listed here (from equation 1 through to equation 13). Therefore, the macrovascular event outcomes are predicted before the microvascular outcomes. Given certain microvascular outcomes can make an individual more

susceptible to macrovascular complications, this could result in an underestimation of the number of macrovascular complications in a given year. Although, as the analysis is long term, this is not thought to have a significant impact on the conclusions drawn.

Cancer

The conceptual model identified breast cancer and colorectal cancer risk as being related to BMI. However, these outcomes were not frequently included in previous cost-effectiveness models for diabetes. Discussion with stakeholders identified the EPIC Norfolk epidemiology cohort study as a key source of information about cancer risk in a UK population [11]. Therefore, we searched publications from this cohort to identify studies reporting the incidence of these risks. In order to obtain the best quality evidence for the relationship between BMI and cancer risk we searched for a recent systematic review and meta-analysis using key terms ‘Body Mass Index’ and ‘Cancer’, filtering for meta-analysis studies.

Breast cancer

Incidence rates for breast cancer in the UK were estimated from the European Prospective Investigation of Cancer (EPIC) cohort [11]. This is a large multi-centre cohort study looking at diet and cancer. In 2004 the UK incidence of breast cancer by menopausal status was reported in a paper from this study investigating the relationship between body size and breast cancer [12]. The estimates of the breast cancer incidence in the UK are reported in ESM Table 43.

ESM Table 43: UK breast cancer incidence

	Number of Cases	Person Years	Mean BMI	Incidence Rate of per person-year	Standard error	Reference
UK pre-menopause	102	103114.6	24	0.00099	0.00009	[12]
UK post-menopause	238	84214.6	24	0.00283	0.00004	[12]

A large meta-analysis that included 221 prospective observational studies has reported relative risks of cancers per unit increase in BMI, including breast cancer by menopausal status [13]. We included a risk adjustment in the model so that individuals with higher BMI have a higher probability of pre-and post-menopausal breast cancer [13]. In the simulation we adjusted the probability of breast cancer according to the difference in the individual's BMI and the average BMI reported in the EPIC cohort. The relative risk and confidence intervals per 5mg/m^2 increase in BMI are reported in ESM Table 44.

ESM Table 44: Relative risk of Breast cancer by BMI

	Mean Relative risk	2.5th Confidence Interval	97.5th Confidence Interval	Reference
UK pre-menopause	0.89	0.84	0.94	[13]
UK post-menopause	1.09	1.04	1.14	[13]

Colorectal cancer

Incidence rates for colorectal cancer in the UK were reported from the European Prospective Investigation of Cancer (EPIC) cohort [11]. The UK incidence of colorectal cancer is reported by gender in a paper from this study investigating the relationship between body size and colon and rectal cancer [14]. The estimates of the colorectal cancer incidence are reported in ESM Table 45.

ESM Table 45: UK colorectal cancer incidence

	Number of Cases	Person Years	Mean Age	Mean BMI	Incidence Rate of per person-year	Standard error	Reference
Male	125	118468	53.1	25.4	0.00106	0.0001	[14]
Female	145	277133	47.7	24.5	0.00052	0.0002	[14]

The risk of colorectal cancer has been linked to obesity. We included a risk adjustment in the model to reflect observations that the incidence of breast cancer is increased in individuals with higher BMI. A large meta-analysis that included 221 prospective observational studies has reported relative risks of BMI and cancers, including colon cancer by gender [13]. We selected linear relative risk estimates estimated from pooled European and Australian populations. In the simulation we adjusted the incidence of colorectal cancer by adjusting the

probability of colorectal cancer by the difference in the individual's BMI and the average BMI reported in the EPIC cohort. The relative risk and confidence intervals per 5mg/m^2 increase in BMI are reported in ESM Table 46.

ESM Table 46: Relative risk of colon cancer by BMI

	Mean Relative risk	2.5th Confidence Interval	97.5th Confidence Interval	Reference
Male	1.21	1.18	1.24	[13]
Female	1.04	1.00	1.07	[13]

Osteoarthritis

Stakeholders suggested that diabetes and BMI should be included as independent risk factors for osteoarthritis. Osteoarthritis had not been included as a health state in previous cost-effectiveness models. The stakeholder group requested that BMI and diabetes be included as risk factors for osteoarthritis based on recent evidence [15]. A search for studies using key words 'Diabetes', 'Osteoarthritis' and 'Cohort Studies' did not identify a UK based study with diabetes and body mass index included as independent covariates in the risk model. Therefore, the Italian study was used in the model.

A study from the Bruneck cohort, a longitudinal study of inhabitants of a town in Italy reported diabetes and BMI as independent risk factors for osteoarthritis [15].

The cohort may not be representative of a UK cohort. However, the individuals are from a European country, the study has a large sample size and has estimated the independent effects of BMI and diabetes on the risk of osteoarthritis. No UK based studies identified in our searches met these requirements. The data used to estimate the incidence of osteoarthritis is reported in ESM Table 47. We did not identify any studies that described diabetes risk on a continuous scale.

ESM Table 47: Incidence of osteoarthritis and estimated risk factors

	No cases	Person years	Mean BMI	Incidence rate	Standard error	Reference
No diabetes	73	13835	24.8	0.0053	0.0006	[15]

	Hazard ratio	2.5th	97.5th			Reference
HR Diabetes	2.06	1.11	3.84			[15]
HR BMI	1.076	1.023	1.133			[15] Personal communication

Depression

Depression was not included as a health state in previous cost-effectiveness models for diabetes prevention. However, a member of the stakeholder group identified that a relationship between diabetes and depression was included in the CORE diabetes treatment model [16]. Therefore, the references used in this model were used.

Depression was included as a health state in the model. However, the severity of depression was not modelled. Some individuals enter the simulation with depression at baseline according to individual responses in the Health Survey for England 2014 questionnaire. Depression is described in the simulation as a chronic state from which individuals do not completely remit. We did not estimate the effect of depression on the longitudinal changes for BMI, glycaemia, SBP and cholesterol. As a consequence, it was not possible to relate the impact of depression to the incidence of diabetes and cardiovascular risk.

In the simulation, individuals can develop depression in any cycle of the model. The baseline incidence of depression among all individuals without a history of depression was estimated from a study examining the bidirectional association between depressive symptoms and type 2 diabetes [17]. Although the study was not from a UK population, the US cohort included ethnically diverse men and women aged 45 to 84 years. We assumed that diagnosis of diabetes and/or CVD increased the incidence of depression in individuals who do not have depression at baseline. We identified a method for inflating risk of depression for individuals with diabetes from the US cohort study described above [17]. The risk of depression in

individuals who have had a stroke was also inflated according to a US cohort study [18]. Odds of depression and odds ratios for inflated risk of depression due to diabetes or stroke are presented in ESM Table 48.

ESM Table 48: Baseline incidence of depression

Baseline Risk of depression			
	Mean	Standard error	
Depression cases in NGT	336		
Person years	9139		
Odds of depression	0.0382	0.002	
Log odds of depression	-3.266		
Inflated risk for Diabetes			
	Mean	2.5th CI	97.5th CI
Odds ratio of diabetes	1.52	1.09	2.12
Log odds ratio of diabetes	0.419		
Inflate risk of stroke			
Odds ratio of stroke	6.3	1.7	23.2
Log odds ratio stroke	1.8406		
NGT Normal Glucose Tolerance			

Mortality

We used the UKPDS-OM2 to estimate the time of death for individuals within the treatment model [8]. UKPDS-OM2 includes four mortality predicting models for different patient circumstances. They developed risk equations for all-cause mortality that suited whether the patient had an event or history of illness or not. Two Gompertz proportional hazard survival models estimated mortality in years in which an event has not occurred. The first model estimated mortality in individuals that had no history of cardiovascular events, while the second model estimated mortality for those with a history of events.

To capture the higher risk of death in years where events occurred, two Logistic models estimated the probability of death in a year in which a stroke, MI, amputation, CHF, IHD or renal failure event occurred. Complications not found to impact the risk of mortality in a current year were blindness and ulcer. Thus, these were not included within the models. One logistic model estimated mortality risk in a year of an event, given the individual had no history of events, while a second logistic model estimated mortality in a year of an event given the individual had a history of events.

Depending on the CVD event outcomes and history of a patient in each year, one of these four models will be appropriate to predict their likelihood of mortality.

These four UKPDS-OM2 risk equations are used to predict mortality conditional on patient demographics, clinical risk factors and current and history of clinical events. The estimated coefficients are reported below in ESM Table 49.

ESM Table 49: UKPDS82 parameters for mortality

	Eq.D1		Eq.D2		Eq.D3		Eq.D4	
	Death in years with no history or events		Death in 1st year of event(s)[a]		Death in years with history but not events[b]		Death in subsequent year/s of event(s)[a]	
Patient-Years	73310		2151		13284		847	
Number of Patients	4993		2151		1612		847	
Number of Events	715		683		386		473	
Functional Form	Gompertz		Logistic		Gompertz		Logistic	
	Mean	SE	Mean	SE	Mean	SE	Mean	SE
Lambda	-10.908	0.304	-6.916	0.591	-9.207	0.534	-4.868	0.828
Gamma	0.098	0.004			0.073	0.007		
Year			0.042	0.010				
Female	-0.229	0.077						
Indian			-0.540	0.205				
Smoking Status	0.379	0.089	0.444	0.117	0.374	0.133		
BMI CAT1					1.083	0.511		
BMI CAT3					-0.293	0.114		
HDL-C							0.068	0.030
Heart rate			0.124	0.032				
MMALB					0.348	0.107		
WBC					0.048	0.011	0.089	0.038
ATFib							1.081	0.396
PVD			0.367	0.130			0.352	0.178
MI History							0.440	0.186
MI Event			1.309	0.158			0.982	0.230
IHD History							-0.507	0.191
IHD Event			0.423	0.168			0.583	0.243
CHF History					0.632	0.130		
Stroke History					0.473	0.122		
Stroke Event			0.547	0.176			-0.619	0.246
Renal Failure History					1.150	0.197	0.961	0.396
Renal Failure Event			0.584	0.305				
Amputation History					0.539	0.198	0.753	0.300
Amputation Event			-0.734	0.321			-1.267	0.344
Amputation 2nd Event							-1.727	0.467

a Any event excluding blindness or ulcer. b used when there is a history of any event

The gompertz model for mortality assumes a baseline hazard where λ = exp(xβ) and γ is an ancillary parameter estimated from the UKPDS data.

$$h(t) = \lambda \exp(\gamma t)$$

The logistic model is described previously.

Calibration

A limitation with the UKPDS risk equations was due to the amount of time since the trial, this has led to a number of studies finding that they have a tendency to overpredict CVD event outcomes in more recent UK and European populations population [19-22]. Duration of diabetes is associated with cardiovascular risk, so it was important to select a target population with a disease duration of less than 2 years [23]. Approximate Bayesian computation (ABC) [24] was used to calibrate first MI and Stroke outcomes to a more recent trial called ADDITION-EUROPE, after 10 years [25]. The ADDITION trial was a multifactorial intervention study, focussing on cardiovascular morbidity and mortality among people with screen-detected type-2 diabetes. The trial included several study sites from the United Kingdom (Cambridge and Leicester), Denmark and the Netherlands. The participants attended health assessments at 1 year and 5 years, with 10-year outcomes collected from GP records and national registries. We chose this dataset as our calibration target as it represented a newly diagnosed diabetes population, with substantive representation from the UK and countries with similar health services. ADDITION-EUROPE found the percent of participants to face an MI or stroke after 10 years of follow up was approximately 3.2% and 2.7% respectively.

ABC sampling methods have been applied to microsimulation models because likelihood-based methods are challenging to implement with complicated models, particularly if they are computational expensive [24]. ABC is part of a class of likelihood-free methods that use the difference between the observed and simulated data to determine the posterior sample, rather than the likelihood function. As with likelihood-function approaches, a prior distribution is specified and samples from this are used in the simulation model. These sample parameters are either accepted or rejected depending how similar the simulated outcome is to the target. The accepted values provide an approximation of the joint posterior distribution.

For the calibration process we generated a baseline population with characteristics to match those reported in the ADDITION-trial. The simulated population were newly diagnosed (diabetes duration = 0), matched key demographic characteristics for age, sex and average metabolic risks (BMI, HbA_{1c}, systolic blood pressure, cholesterol).

We compared the rejection sampling method with a tolerance of 0.1, a tolerance of 0.05, a tolerance of 0.01 and an MCMC approach using the marjoram method. For the final calibration run we used the rejection sampling algorithm to generate 1000 samples from the joint posterior distribution, using a tolerance of either 0.1, 0.05, or 0.01. At these tolerance parameters we needed to run 20000, 40000, and 200000 samples respectively to generate the posterior samples. At a tolerance of 0.01 we were able to produce sampled incidence of MI and stroke, close to the 95% confidence interval estimates from ADDITION.

When implemented into the GLOW model the calibration achieved a 10-year MI incidence of approximately 4.1% and a 10-year stroke incidence of approximately 3.5% within the baseline population. ESM Figure 5 illustrates the cumulative incidence of events with and without calibration. Calibrating MI and stroke estimated within the Diabetes treatment model to these levels, resulted in the risk of death decreasing from 26% to 23% after 10 years. This is compared to 15.2% death rate in the ADDITION-EUROPE trial. In later years the calibration of stroke and MI increase the incidence of other comorbidities indirectly because of the increased life expectancy of the population.

ESM Figure 5: A comparison of the cumulative frequency of modelled outcomes with and without calibration

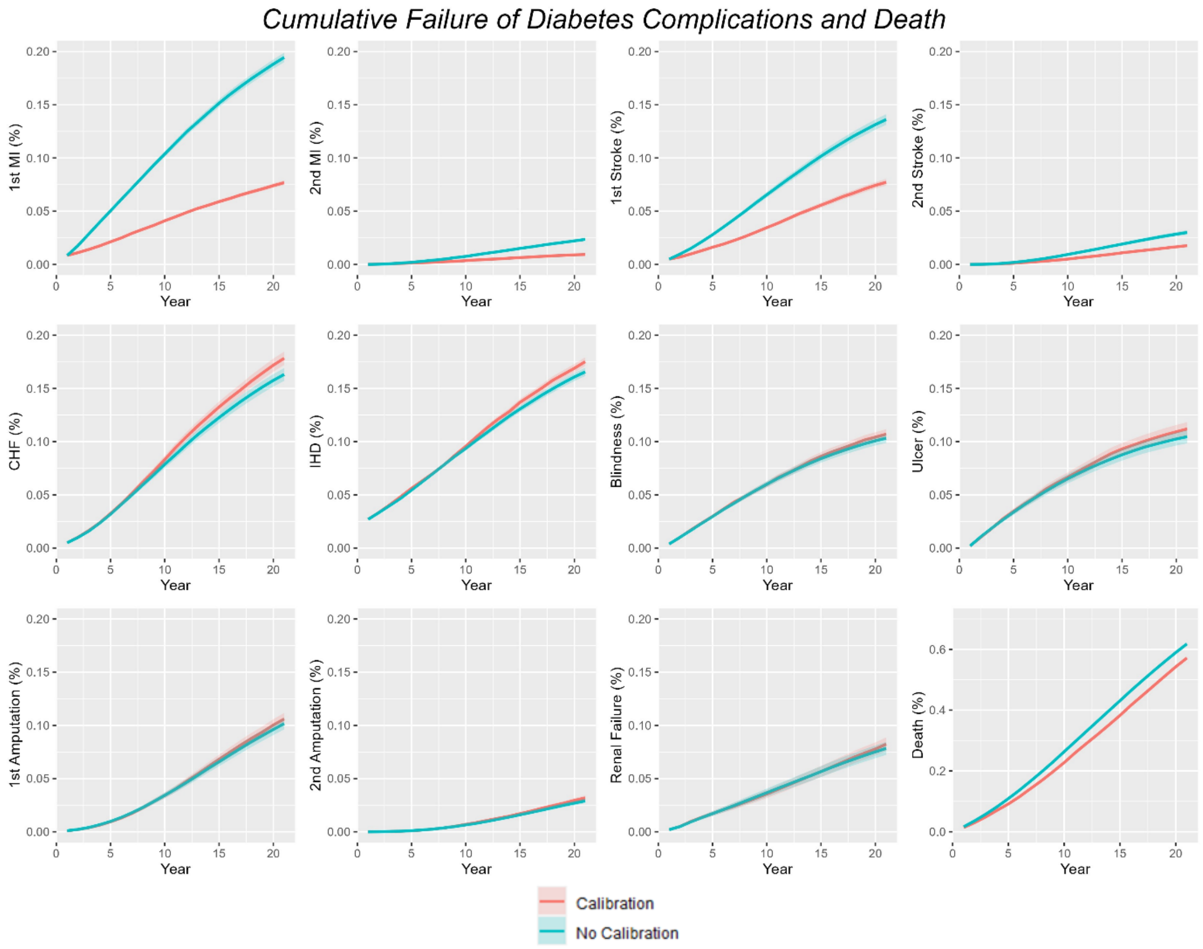

Direct Health Care Costs

At any given time period of the model, individuals can have multiple health complications that incur direct healthcare costs. Some of the health states are mutually exclusive; however an individual can accrue multiple complications within the model. Each health state is associated with an average cost, which is accrued by all individuals for every time period for which the state is indicated. Resource use for each comorbidity is added together and no savings are assumed to be made from the use of the same resources for two or more comorbidities for an individual.

In some instances, we have adopted costs and prices from old studies. We have inflated all prices and costs to 2020 prices (GBP) using inflation indices reported in the Personal Social Services Research Unit (PSSRU) [26].

GP attendance

The costs of each visit to a General Practitioner were estimated at £39, as reported in the Personal Social Services Research Unit [26]. This is under the assumption that each visit is on average 9.22 long.

Diabetes

We were advised by stakeholders to model a simplified diabetes treatment pathway. It was recommended that a single annual cost of prescriptions be applied to all patients diagnosed with diabetes. Initially we explored this as an option but concluded that the timing of more costly treatments for type 2 diabetes is important because treatment costs will be discounted. The model assesses interventions that lower HbA_{1c} and so have the potential to impact on the level of treatment required.

We decided to implement a three-stage treatment regimen as a trade-off between model simplicity and capturing key cost differences between the interventions. In the early phases of the modelling the model assumes that patients start on a low cost regimen. If HbA_{1c} increases above a threshold the individual is prescribed an additional medication in addition to existing therapies. The individual continues to receive combined therapy for a period of time until they require insulin. A summary of unit costs used for diabetes maintenance is detailed in ESM Table 50.

ESM Table 50: Unit costs used for diabetes maintenance

**Resource**	**Assumption for costs**	**Source from Input para excel**	**Unit Cost before inflation**	**Unit cost after inflation**	**Inflation**	**Annual utilisation**	**Cost per year**
Nurse at GP (Advanced Nurse)	Advanced GP Nurse per hour (with qualifications)	[26]	44	44	1	0.25 (15-minute appointment)	11

GP Nurse	Band 4 – Qualified Nurse at GP practice per hour - 10 minute appointment	[26]	33	33	1	0.167 (10 mins)	5.50
Urine sample	Clinical Biochemistry	[27]	2	2	1	1	2
Eye screening	Non-Consultant Lead Ophthalmology	[27]	140.80	140.80	1	1	140.80
HbA_{1c}	Haematology	[27]	4	4	1	1	4
Lipids	Clinical Biochemistry	[27]	2	2	1	1	2
Liver function	Clinical Biochemistry	[27]	2	2	1	1	2
B12	Clinical Biochemistry	[27]	2	2	1	1	2

Monotherapy

The cost of initial monotherapy were calculated using GLoW trial data on participant medications and accounts for both quantity and type of diabetes they are on before and after HbA_{1c} decline. The unit drug costs and dose for all therapies prescribed for participants in the GLoW trial are reported in ESM Table 51.

ESM Table 51: BNF drug costs per dose option.

**Drug Name**	**Drug code**	**Dose Option 1 (Lower Dose)**				**Dose Option 2 (Higher Dose)**			
		**Packet price**	**Packet size**	**Packet dose**	**Packet dose measure**	**Packet Price**	**Packet size**	**Packet dose**	**Packet dose measure**
metformin	a10ba02	0.82	28	500	mg				
dulaglutide	a10bj05	73.25	4	0.75	mg/0.5ml	73.25	4	1.5	mg/0.5ml
empagliflozin	a10bk03	36.59	28	10	mg	36.59	28	25	mg
gliclazide	a10bb09	1.13	28	40	mg	1.04	28	80	mg
sitagliptin	a10bh01	33.26	28	100	mg				
alogliptin	a10bh04	26.6	28	25	mg				
saxagliptin	a10bh03	31.6	28	2.5	mg	31.6	28	5	mg
glimepiride	a10bb12	0.87	30	1	mg	0.93	30	2	mg
linagliptin	a10bh05	33.26	28	5	mg				
semaglutide	a10bj06	78.48	30	7	mg	78.48	30	14	mg
glipizide	a10bb07	1.26	28	5	mg				
insulin (human)	a10ac01	19.08	5	300	units/3ml - 3ml pens				
canagliflozin	a10bk02	39.2	30	100	mg	39.2	30	300	mg
insulin degludec	a10ae06	46.6	5	300	units/3ml - 3ml pens				
liraglutide	a10bj02	78.48	2	18	mg/3ml - 3ml pens				
insulin aspart	a10ab05	30.6	5	300	units/3ml - 3ml pens				

Other resource use costs and resource utilisation assumptions for diabetics receiving initial monotherapy are detailed in ESM Table 52.

ESM Table 52: Drug costs and resource utilisation costs for diabetes monotherapy

**Resource**	**Assumption for costs**	**Source from Input para excel**	**Unit Cost before inflation**	**Unit cost after inflation**	**Inflation**	**Annual utilisation**	**Cost per year**
Annual Drug Cost	Estimated from GLOW trial data, incorporating any diabetes medication taken by individual in monotherapy. Drug costs estimated from BNF. See ESM Table 51	GLOW	33.89	33.89	1	1	33.89
Nurse at GP (Advanced Nurse)	Advanced GP Nurse per hour (with qualifications)	[26]	44	44	1	0.25 (15-minute appointment)	11
GP Nurse	Band 4 – Qualified Nurse at GP practice per hour - 10 minute appointment	[26]	33	33	1	0.167 (10-minute appointment)	5.50
Urine sample	Clinical Biochemistry	[27]	2	2	1	1	2
Eye screening	Non-Consultant Lead Ophthalmology	[27]	140.80	140.80	1	1	140.80
HbA_{1c}	Haematology	[27]	4	4	1	1	4
Lipids	Clinical Biochemistry	[27]	2	2	1	1	2
Liver function	Clinical Biochemistry	[27]	2	2	1	1	2
B12	Clinical Biochemistry	[27]	2	2	1	1	2
						Total annual cost	70.71
						Total annual cost in use	£594

Remission

Individuals are assumed to transition from monotherapy to being on no diabetes related drugs, or 'remission', if their HbA_{1c} drops below 6.5% [28], and the annual drug cost is removed from their total annual cost of diabetes.

Dual Therapy

Simulated individuals experience an annual increase in HbA_{1c}. Bennett et al. (2014), when assessing treatment escalation in patients from The Health Improvement Network (THIN), estimated that individuals were, on average, switched to dual treatment if HbA_{1c} increases above 8.48% [29]. While this is higher than recommendations, it represents the potential delay in escalation in therapy that may occur in reality. Within the model, the individual is switched to a dual treatment in the first annual cycle in which HbA_{1c} exceeds 8.48%. For

costing purposes, the second drug to be added to initial therapy was Sitagliptin, which is reported in the British National Formulary to cost £1.19 per day. Belsey et al. (2009) report that 48% of patients used monitoring strips at a mean weekly consumption of 3.3. ESM Table 53 reports the other resource use costs and utilisation assumptions for diabetics receiving initial therapy plus Gliptins.

ESM Table 53: Drug costs and resource utilisation costs for initial therapy and Gliptins

Resource	Assumption for costs	Source from Input para excel	Unit Cost before inflation	Unit cost after inflation	Inflation	Annual utilisation	Cost per year
Sitagliptin	100mg per day	[30] 28-tabs of 100mg = £33.26 £1.19 per tablet	1.19	1.19	1	365	434.35
Annual Drug Cost	Estimated from GLOW trial data, incorporating any diabetes medication taken by individual in monotherapy. Drug costs estimated from BNF. (ESM Table 51)	GLOW	33.89	33.89	1	1	33.89
Self-monitoring strips	50 strip pack from National Diagnostic Products	[30] 50 strip pack, National Diagnostic Products, 11p per strip	0.11	0.11	1	82.2	9.04
Nurse at GP (Advanced Nurse)	Advanced GP Nurse per hour (with qualifications)	[26]	44	44	1	0.25 (15-minute appointment)	11
GP Nurse	Band 4 – Qualified Nurse at GP practice per hour - 10 minute appointment	[26]	33	33	1	0.167 (10-minute appointment)	5.50
Urine sample	Clinical Biochemistry	[27]	2	2	1	1	2
Eye screening	Non-Consultant Lead Ophthalmology	[27]	140.80	140.80	1	1	140.80
HbA_{1c}	Haematology	[27]	4	4	1	1	4
Lipids	Clinical Biochemistry	[27]	2	2	1	1	2
Liver function	Clinical Biochemistry	[27]	2	2	1	1	2
B12	Clinical Biochemistry	[27]	2	2	1	1	2
					Total annual cost		514.10
					Total annual cost in use		£1037

Insulin plus Oral Anti-diabetics

The second major treatment change is assumed to be initiation of insulin. Bennett et al (2014) estimated that, on average, individuals switch to insulin therapy if HbA_{1c} increases above 9.5%. Within the model, the individual is switched to insulin the first annual cycle at which HbA_{1c} exceeds 9.5%. The total resource use and costs of this health state are reported in

ESM Table 54. The assumptions used to generate a cost for insulin is reported in ESM Table 55.

ESM Table 54: Drug costs and resource utilisation costs for insulin and oral anti-diabetics

Resource	Assumption for costs	Source	Unit Cost before inflation	Unit cost after inflation	Inflation	Annual utilisation	Cost per year
Insulin Treatment Costs	Total Annual Cost	See ESM Table 55.	1,013.51	1328.02	1.31	1	1328.02
Nurse at GP (Advanced Nurse)	Advanced GP Nurse per hour (with qualifications)	[26]	44	44	1	0.75 (3x 15- minute appointments)	33
GP Nurse	Band 4 – Qualified Nurse at GP practice per hour - three 10 minute appointment	[26]	33	33	1	0.5 (3x 10-minute appointments)	16.5
Urine sample	Clinical Biochemistry	[27]	2	2	1	3	6
Eye screening	Non-Consultant Lead Ophthalmology	[27]	140.80	140.80	1	1	140.80
HbA_{1c}	Haematology	[27]	4	4	1	3	12
Lipids	Clinical Biochemistry	[27]	2	2	1	3	6
Liver function	Clinical Biochemistry	[27]	2	2	1	3	6
B12	Clinical Biochemistry	[27]	2	2	1	3	6
					Total annual cost		1436.75
					Total annual cost in use		£1960

ESM Table 55: Breakdown of unit costs used to calculate the total cost of insulin

	Cost before Inflation (2006 prices)	Inflation	Unit cost after inflation	Source
Insulin Glargine	£628.44			[31]
Oral anti-diabetics	£43.68			[31]
Reagent test strips	£221.43			[31]
Hypoglycaemic rescue	£23.43			[31]
Pen delivery devices	£54.79			[31]
Sharps	£68.82			[31]
Total cost per year	£1,013.51	1.31	1328.02	

BNF £35.28 per 5 pen/cartridge 100 units per ml, £7.06 per pen/cartridge

Hospitalisation Costs

The cost of both inpatient and non-inpatient resource use, due to a diabetes-related complication, were estimated in a study using UKPDS patient-level data [32]. This study considers costs associated with consultations, visits, admissions, and procedures for event outcomes in the year in which the event occurred and in subsequent years by matching UKPDS participants to HES hospital records. The inpatient hospitalisation costs were estimated using two-part multivariate regression analysis. The regression coefficients were then used to estimate the mean cost of hospitalisation (considering the probability of hospitalisation) for the average representative individual (a 60-year-old male). Within this estimation, they estimated the mean yearly cost of hospitalisation for a year in which the individual had a cardiovascular event, but also for years in which no event occurs. This 'no event' hospitalisation cost (ESM Table 59)

was added to the cost of diabetes treatment in this model, presented in the tables previously, with the understanding that some of these individuals will incur some cost of hospitalisation, even when they don't experience a cardiovascular event. It is an average hospitalisation cost of having no event, with the consideration that approximately 16% of individuals (male, aged 60) will go so hospital for a non-cardiovascular event purpose [19]. This cost is added to the diabetes treatment costs at each line of therapy. Combining the cost of hospitalisation when no event has occurred to the cost of diabetes treatments ensures that the hospitalisation costs associated with not having an event are included in the model for all individuals, as everyone is on some form of diabetes treatment. This cost is removed from the valuations of average hospitalisation costs during a year of having a cardiovascular event to better represent the change in cost within these years. The hospitalisation costs estimated in this paper does not consider prescribed drugs or outpatient care, therefore double counting of these costs is avoided when adding treatment and hospitalisation costs together.

Statins

We assumed that individuals who are prescribed statins receive a daily dose of 40mg of generic Simvastatin. The British National Formulary reports a cost of approximately 5p per day [30]. The individual remains on statins for the rest of their life. ESM Table 56 reports the derived annual costs for statins. The cost of GP attendance was not included in the cost of statins to avoid double counting of GP attendance.

ESM Table 56: Annual treatment costs of statins

**Resource**	**Assumption for costs**	**Source**	**Unit Cost before inflation**	**Unit cost after inflation**	**Inflation**	**Annual utilisation**	**Cost per year**
Statins	Simvastatin 20mg (28 tablets from AAH = 0.45, 1.6p per tablet)	[30]	0.027	0.027	1	730	19.71
						Total annual cost	19.71

Anti-hypertensives

The National institute for Health Care and Excellence reported estimates for the costs of treating hypertension in cost effectiveness analysis report [33]. A range of costs of annual drug and monitoring costs were reported, and the midpoint of this range informed the costs

used with this model. This provided a treatment cost within the first year of treatment, reported in ESM Table 57 and the cost of subsequent years of treatment, reported in 5. These were inflated to 2020 prices [26]. Due to the number of different anti-hypertensive treatments available and possibilities for combination therapies, using the cost from this study of prescriptions was preferred to using costs directly from the BNF.

ESM Table 57: Annual cost of anti-hypertensive treatment expenditure per patient in first year (£)

Resource	Assumption for costs	Source	Unit Cost before inflation	Unit cost after inflation	Inflation	Annual utilisation	Cost per year
Drug Costs	Weight averages of BNF drug costs based on distribution of number and class of drugs.	[33]	18.02	18.575	1.03	1	18.575
Monitoring Costs	Monitoring costs during first year of treatment	[33]	121.5	125.242	1.03	1	125.242
						Total annual cost	143.82

ESM Table 58: Annual cost of anti-hypertensive treatment expenditure per patient in subsequent years (£)

Resource	Assumption for costs	Source	Unit Cost before inflation	Unit cost after inflation	Inflation	Annual utilisation	Cost per year
Drug Costs	Weight averages of BNF drug costs based on distribution of number and class of drugs.	[33]	18.02	18.575	1.03	1	18.575
Monitoring Costs	Monitoring costs during subsequent years of treatment	[33]	75	77.310	1.03	1	77.310
						Total annual cost	95.89

Cardiovascular Events

The cost of an occurrence of a macrovascular event occurring either in the current or in a previous period were obtained from a study combining UKPDS data with HES hospital records [32]. A brief explanation of this study was offered previously. This study also collected information on non-inpatient costs of cardiovascular events through a series of patient questionnaires about resource use (consultations with general practice doctors and nurses, opticians, dietitians, or health visitors either in person, over the phone or at home) over recent months and years. This was used to generate an average annual non-inpatient cost for a representative individual (male, aged 60) for a year in which they faced a cardiovascular event, had previously had a cardiovascular event, or had never faced an event. A summation

of the hospitalisation and non-impatient costs, minus the costs they would face had they not had the event, generates the total annual cost of cardiovascular event outcomes. The cost of an occurrence of an amputation or retinopathic event is also taken from this study while the cost of nephropathic outcomes and the cost of an ulcer were taken from Kerr et al [34] and the CKD guidelines respectively. ESM Table 59 described the costs, resource use assumptions, and sources of the costs of cardiovascular event outcomes that were used in this study. Given the date these costs are reported from, they have been inflated to 2020 prices.

ESM Table 59: Cost of hospitalisation and non-inpatients costs relating to diabetes and its complications

**Event**	**When Cost Incurred**	**Hospitalisation Costs**	**Non-Inpatient Costs**	**Total Cost**	**Source**	**Inflation**	**Total Cost Inflated**	**Cost per year used (removing cost of no event)**
No Event		459	532	991	[32]	1.14	1129.146	
MI Event	Year of Complication	6379	963	7333		1.14	8365.476	£7,236
MI History	Subsequent Years	1154	671	1825		1.14	8996.704	£950
Stroke Event	Year of Complication	6805	1091	7896		1.14	8727.883	£7,868
Stroke History	Subsequent Years	1125	756	1881		1.14	2143.212	£1,014
CHF Event	Year of Complication	3191	979	4170		1.14	4751.299	£3,622
CHF History	Subsequent Years	1473	973	2446		1.14	2786.973	£1,658
IHD Event	Year of Complication	9767	864	10631		1.14	12112.96	£10,984
IHD History	Subsequent Years	1215	654	1869		1.14	2129.539	£1,000
Amputation Event	Year of Complication	9546	2699	12245		1.14	13951.95	£12,823
Amputation History	Subsequent Years	1792	1611	3403		1.14	3877.379	£2,748
Blindness Event	Year of Complication	1355	1790	3145		1.14	3583.413	£2,454
Blindness History	Subsequent Years	453	738	1191		1.14	1357.026	£228
Fatal MI	Year of Complication	1521		1521		1.14	1733.028	£604
Fatal IHD	Year of Complication	3766		3766		1.14	4290.981	£3,162
Fatal Stroke	Year of Complication	3954		3954		1.14	4505.188	£3,376
Renal Failure Event	Year of Complication			£20,897	[2]	1.03	£21,540.63	£21,541
Renal Failure History	Subsequent years			£8,332		1.03	£8,588.63	£8,589
Ulcer				3520	[34]	1.03	£3,628.42	£3,628.42

When estimating the coefficients between the average annual hospitalisation and non-inpatient costs and the current or history status of cardiovascular events, there was a negative

coefficient for having a fatal myocardial infarction. While the average annual spend for a representative individual (male, aged 60) who has a fatal myocardial infarction was still positive, when removing the costs associated events with having no event, the cost becomes small. With this in mind, to avoid negative costs in the probability sensitivity analysis, the cost of an event occurring is set to zero if the cost of no event exceeds the cost of the event.

Osteoarthritis, Cancer, and Depression

The cost of alternative diagnoses beyond cardiovascular outcomes, including osteoarthritis, breast cancer and colorectal cancer, are reported in ESM Table 60.

The cost of breast and colorectal cancer is estimated as a one-off fixed cost at diagnosis in the model. This simplifying assumption means that the cost of cancer treatment is independent of survival. We acknowledge that this assumption will affect the timing of costs because all costs are imposed in the first year and subject to less discounting. However, we anticipate that the impact on overall outcomes will not be substantial. A large proportion of costs will be incurred in the first year of treatment (surgery, chemotherapy, radiotherapy). Costs in subsequent years will be lower for patients who achieve remission and survival will be short in patients who relapse. Therefore, the costs are likely to be skewed to the early years post diagnosis.

ESM Table 60: Annual costs for osteoarthritis, cancer and depression (£)

**Diagnosis**		**Yearly Total Cost**	**Inflation**	**Source**	**Inflated Yearly total cost**	**Resource Use**	**Health Care Cost**
Osteoarthritis		730	1.226	[35]	894.91		894.91
Depression				ESM Table 62			613.94
Cancer Breast	Total 9-year cost for stage 1-2 ages 18-64	22502	1.18	[36]	26622.21	0.49	13078.8
	Total 9-year cost for stage 1-2 ages 65+	35244	1.18		41697.32	0.066	2740.6
	Total 9-year cost for stage 3-4 ages 18-64	19479	1.18		23045.69	0.37	8524.7
	Total 9-year cost for stage 3-4 ages 65+	25698	1.18		30403.41	0.073	2222.3
	Weighted average total 9-year cost	22454.87	1.18				26566.5
Cancer Colorectal	Total 9-year cost for stage 1-2 ages 18-64	12938	1.18		15307.00	0.12	1871.83
	Total 9-year cost for stage 1-2 ages 65+	21128	1.18		24996.62	0.14	3617.36
	Total 9-year cost for stage 3-4 ages 18-64	13843	1.18		16377.71	0.38	6242.53
	Total 9-year cost for stage 3-4 ages 65+	17282	1.18		20446.41	0.35	7193.86
	Weighted average total 9-year cost	15996.55	1.18				18925.58

Depression

Depression is modelled as a chronically recurrent disorder, with patients experiencing further depressive episodes after remission. In the model it is assumed that patients continue to incur costs of depression following an initial diagnosis. These costs reflect ongoing resource use to deal with relapse and prevention of relapse.

A recent trial to prevent secondary depressive episodes collected comprehensive cost data from a sample of individuals with depression [37]. The resource uses identified in the control arm were extracted to estimate the costs of depression. The costs from this data (inflated to 2019 prices) were not implemented directly into the SPHR diabetes prevention model as this would have over-estimated the number of GP visits. The model already accounts for GP attendance due to depression. Therefore, a revised estimate of the cost of depression, excluding GP consultation was estimated using updated unit costs. The resource use estimates, and revised unit cost estimates used to generate a cost of depression excluding GP utilisation are reported in ESM Table 61.

ESM Table 61: Breakdown of depression costs

Resource	Assumption for costs	Source	Unit Cost before inflation	Unit cost after inflation	Inflation	Annual utilisation	Cost per year
Nurse at GP (Advanced Nurse) at surgery	Advanced GP Nurse (with qualifications) - 10 minute appointment	[26]	7.33	7.33	1	1.52	11.15
Nurse at GP (Advanced Nurse) at home visit	Advanced GP Nurse (with qualifications) - 30 minute appointment	[26]	22.00	22.00	1	0.02	0.44
Nurse at GP (Advanced Nurse) telephone	Advanced GP Nurse (with qualifications) - 10 minute appointment	[26]	7.33	7.33	1	0.11	0.81
Health visitor	Health visitor[Advanced GP Nurse (with qualifications)] – 30 minute appointment	[26]	22.00	22.00	1	0.05	1.10
District nurse	Community nurse [Advanced GP Nurse (with qualifications)] - 30 minute appointment	[26]	22.00	22.00	1	0.01	0.22
HCA phlebotomist	Clinical support worker 10 mins	[37]	2.17	2.644	1.219	0.31	0.82
Other primary care	Advanced nurse with qualifications	[37]	14.48	17.645	1.219	0.19	3.35
Out of hours	Inflated of trial costs	[37]	5.13	6.251	1.219	0.23	1.44
NHS direct	Inflated of trial costs	[37]	1.89	2.303	1.219	0.09	0.21
Walk-in centre	Inflated of trial costs	[37]	6.77	8.250	1.219	0.21	1.73
Prescribed medications	Inflated of trial costs	[37]	61.87	75.395	1.219	7.74	583.56
Secondary care	Emergency Medicine, Any Investigation	[37]	23.85	29.064	1.219	0.26	7.56
							613.32

Social Care costs

In this analysis the social care costs refer to the public and private costs incurred with social care as a consequence of a diagnosis with osteoarthritis or stroke. Social care costs associated with the other health outcomes of the model are not included in this estimate. This is likely to under-estimate the overall cost of social care in the population. However, reliable social care costs for other conditions are very hard to obtain because they are less commonly incurred in the prevalent patient population and more likely to be attributed to other factors or ageing more generally.

Osteoarthritis

The annual cost of osteoarthritis were estimated in a report in 2010 [35]. The estimated annual cost of osteoarthritis was £783 in 2008. In the study 93% of the costs were attributable to direct medical costs and 7% to social care. Therefore, cost of social care costs in 2020 prices at £65.

Stroke

The community costs in the first year following stroke were estimated using the mean number of resource use days from South London Stroke Register [38] with updated cost estimates [26, 39]. The average number of days at day centres, nursing homes, residential home, sheltered accommodation and were used to estimate the social care costs (ESM Table 62**Error! Reference source not found.**).

ESM Table 62: Costs of stroke

	Mean number of days	Source	Unit cost per day (Inflated to 2020)	Source	Total cost
Day Centre	3.9	[38]	£66	[26]	257.40
Nursing Home	16.9	[38]	£112.43	[26]	1900.04
Residential Home	8.5	[38]	£132.71	[26]	1128.07
Sheltered Home	8.1	[38]	£76.30	[39]	618.00
Total cost					3903.51

Utilities

Baseline Utility

Summary statistics for baseline utility were generated from the Glow trial data The tariffs for the responses to the 5 level EQ-5D were derived from a UK population study [40]. Utility was assumed to decline due to ageing independent of health status according to patterns observed in the UK population [41]. Utility decrements are associated with age with a quadratic relationship, allowing decrements to accelerate in effects at older ages. Additional utility adjustments were applied based on the BMI status of an individual. BMI above the average for the population at baseline were associated with lower utility, and below BMI at baseline were associated with higher utility. Differences in utility from the population average were multiplied by a -0.011 (95%CI -0.007 to -0.016) based on observed associations from other weight loss trials [42].

Utility Decrements

The utility decrements for long term chronic conditions were applied to the age adjusted EQ-5D score, following a quadratic distribution observed in the general population [41]. Cardiovascular disease, renal failure, amputation, foot ulcers, blindness, cancer, osteoarthritis and depression were all assumed to result in utility decrements. The utility decrements are measured as a factor which is applied to the individual's age and BMI adjusted baseline. BMI adjusted baseline utility is calculated by multiplying the difference in sample populations BMI from the average BMI[43] by the BMI decrement [42] and adding this to the age adjusted utility. If individuals have multiple chronic conditions the utility decrements are multiplied together to give the individual's overall utility decrement from comorbidities and complications, in line with current NICE guidelines for combining comorbidities [44].

Due to the number of health states, it was not practical to conduct a systematic review to identify utility decrements for all health states. A pragmatic approach was taken to search for health states within existing health technology assessments for the relevant disease area or by

considering studies used in previous economic models for diabetes prevention. Discussions with experts in health economic modelling were also used to identify prominent sources of data for health state utilities.

The mean absolute decrement estimated in each study is reported alongside the baseline utility for each study in ESM Table 63. The utility factor was estimated by dividing the implied health utility with the comorbidity by the baseline utility.

ESM Table 63: Utility decrement factors

	**Mean Absolute decrement**	**St. error absolute decrement**	**Baseline Utility**	**Multiplicative Utility Factor**	**Source**
Foot ulcer	-0.17	0.019	1.027	0.834	[45]
Amputation	-0.172	0.045	0.807	0.787	[46]
Blind	-0.033	0.027	0.807	0.959108	[46]
Renal failure	-0.049	0.068	0.83	0.940964	[43]
Myocardial Infarction y1	-0.065	0.03	0.807	0.919455	[46]
Myocardial Infarction y2	-0.008	0.024	0.807	0.909542	[46]
Stroke y1	-0.099	0.035	0.83	0.880723	[43]
Stroke y2	-0.099	0.035	0.83	0.880723	[43]
Breast Cancer	-0.060	0.008	0.791	0.913	[47]
Colorectal Cancer	-0.060	0.008	0.791	0.913	[47]
Osteoarthritis	-0.101	0.069	0.807	0.875465	[48]
Depression	-0.116		0.791	0.852986	[49]
Congestive Heart Failure	-0.045	0.040	0.83	0.945783	[43]
Ischaemic Heart Disease	-0.01	0.029	0.83	0.987952	[43]
MMALB	-0.048	0.022	1.027	0.953262	[45]
PVD	-0.061	0.015	1.027	0.940604	[45]
ATFIB				1	Assumption
	**Utility per Unit**	**St.error**			
BMI	-0.011	0.002			[42]

Intervention Effects

Mixed-Effects Regression

A mixed-effects regression was used to estimate the 12-month difference in risk factor outcomes, HbA_{1c} (%) and BMI, between the intervention group DEW and control DE. A random intercepts linear regression models was performed with the risk factor change from baseline to 6 and baseline to 12 months as the dependent variable. The model included the treatment group, a binary variable indicating whether it is 6-month or 12-month value, an interaction term between treatment group and the time point, the baseline risk factor level, as well as randomisation stratifiers of gender and duration of diabetes, as fixed effects, allowing for random intercept at the individual level. The regression specification for HbA_{1c} is:

$$\begin{aligned} Risk\ Factor\ \Delta \text{ from baseline to time } \mathrm{t}_i \\ &= \beta_0 + \mu_i + \beta_1(treatment\ group_i) + \beta_2(time = 6months_i) \\ &+ \beta_{12}(time = 6\ months_i * treatment\ group_i) \\ &+ \beta_3(Baseline\ Risk\ Factor_i) + randomisation\ stratifiers + \varepsilon_{it} \end{aligned}$$

Where i represents the individual, t represents the time (either 6 or 12 months), μ represents the between-individual error and ε represents the overall residual error. The label "risk factor" here represents either HbA_{1c} (%) or BMI (kg/m^2). A mean difference in HbA_{1c} will be negative if the DEW treatment generated a larger HbA_{1c} reduction, or a smaller HbA_{1c} increase, than the DE treatment.

The 12-month difference in HbA_{1c} between treatment groups is represented by β_1. The regression coefficients for HbA_{1c} change are presented in ESM Table 64.

ESM Table 64: Treatment effect on HbA_{1c} (%) for DEW vs DE at 12 months

**Fixed Effects**	**Mean**	**2.5% CI**	**97.5% CI**	**t Value**
Intercept	2.71	2.14	3.28	9.37
Baseline HbA_{1c} (%)	-0.36	-0.43	-0.28	-9.32
Treatment Group (DEW = 1, DE = 0)	-0.08	-0.27	0.12	-0.77
Time (1= 6 months, 0=12 months)	-0.12	-0.26	0.02	-1.69
Treatment Group * Time	-0.09	-0.29	0.11	-0.88
Male (=1)	-0.15	-0.32	0.02	-1.71
Diabetes Duration less than 1 year (=1)	-0.15	-0.32	0.03	-1.62

Random Effects	Parameter	SD	
Individual id	intercept	0.77	
Residual		0.65	

The mean treatment effect on HbA_{1c} implemented into the model is a -0.08% change for individuals receiving DEW compared to those receiving DE. This difference is applied as a reduction in HbA_{1c} for the DEW treatment group compared to the usual care comparison arm which follows the models HbA_{1c} trajectory (See Section 3). The reduction is applied uniformly across the entire sampled population and assumed to decay linearly over 5 years, until the DEW treatment group return to the usual care trajectory by the 6th year cycle. This treatment will vary through the PSA, with parameter uncertainty reflected using a standard error of 0.099, calculated using the 95% confidence intervals and the mean difference, assuming a normal distribution [50].

The 12-month difference in BMI is estimated using the same methodology and informed by β_1 from the regression outputs. The regression coefficients are reported in ESM Table 65.

ESM Table 65: Treatment effect on BMI for DEW vs DE at 12 months

BMI				
**Fixed Effects**	**Mean**	**2.5% CI**	**97.5% CI**	**t Value**
Intercept	1.38	0.34	2.42	2.60
Baseline BMI	-0.06	-0.09	-0.03	-4.38
Treatment Group (DEW = 1, DE = 2)	-0.50	-0.92	-0.07	-2.30
Time (1= 6 months, 0=12 months)	0.25	-0.02	0.52	1.83
Treatment Group * Time	-0.17	-0.56	0.21	-0.89
Male (=1)	-0.03	-0.39	0.33	-0.18
Diabetes Duration less than 1 year (=1)	-0.01	-0.37	0.35	-0.06
**Random Effects**	**Parameter**	**SD**		
Individual id	intercept	1.59		
Residual		1.17		

Similarly, a mean difference of -0.06kg/m^2 is applied to the BMI across the entire population in the DEW treatment arm and is assumed to linearly decay over 10 years. This treatment will vary through the PSA, with parameter uncertainty reflected using a standard error of 0.214,

calculated using the 95% confidence intervals and the mean difference, assuming a normal distribution [50].

Intervention effect beyond 12 months

Research has indicated that the intervention effect after 12 months decays over time, but with varying information as to how quickly it decays.

Analysis of the WRAP 5-year follow-up identified that differences in weight loss were maintained after 5 years of follow-up but were not statistically significantly different (–0·96 (–2·90 to 0·97) kg for 12-week vs brief intervention). Therefore, in the long-term modelling it was assumed that the benefits of weight loss were diminishing linearly over time, and returned to the natural history projection after 10 years. Differences in HbA_{1c} were not sustained after 5 years of follow-up so the duration of benefit on HbA_{1c} was assumed to diminish linearly over time up to 5 years after the intervention.

A systematic review and meta-analysis report pooled estimates of weight loss and HbA_{1c} reduction for weight loss/maintenance programmes in populations with type-2 diabetes after 12 months of follow-up [51]. They reported pooled estimates of differences in pooled control arms and intervention groups beyond 12 months of follow-up for weight loss. The estimates indicate that differences in the % weight loss between intervention and control are maintained after up to 4 years of follow-up. It should be noted that the uncertainty in these estimates is not reported. Pooled estimates for the difference in HbA_{1c} beyond 12 months were not reported. One study included in this review was an intervention targeted at those with newly diagnosed diabetes [52]. This was a randomised controlled trial of a Mediterranean-style diet (low carbohydrate) based in Italy. Participants in the Mediterranean diet group experienced a significant change in BMI and HbA_{1c} at 12 months compared to the control group (low-fat diet) and these differences remained significant at 4 years.

A systematic review and meta-analysis of behaviour change techniques in diet and physical activity interventions reported difference in weight and HbA_{1c} after 2 years of follow-up. Differences in HbA_{1c} were statistically significant, but differences in weight were potentially clinically important, but not statistically significant [53].

The Look AHEAD trial reported the effect of a weight loss programme in individuals with type-2 diabetes on weight loss after 8 years of follow-up [54]. However, the estimates from this study are considered less relevant to this analysis because the intervention included weight maintenance beyond 12 months, and included a large proportion of participants with diabetes duration more than 3 years.

Based on the available evidence it is clear that the benefits of lifestyle interventions in populations with type-2 diabetes can extend beyond 12 months of follow-up. There is evidence from a small number of studies to support an assumption that statistically significant benefits will be observed after 2 and 4 years of follow-up. It is likely that the initial differences in effectiveness will decline with time. In this modelling study we will assume that the effects of the intervention on weight loss after 12 months will decline over time, and no differences will be observed after 10 years of follow-up. Differences in HbA_{1c} will be assumed to reduce linearly over time up to 5 years post intervention. This assumption has been used in other modelling studies [55].

Intervention Costs

The costs for the Weight Watchers diabetes education programme were supplied by the commercial provider of the course at a cost of £325.20. The cost of the course was not discounted if participants took the course online.

The costs for the DESMOND diabetes education service were estimated in a micro-costing exercise. The costs associated with the service are disaggregated into four components.

1. The cost of the license which is covered by the face to face and online workshop.

2. The CCG wide costs which need to be spread across all participants attending the face to face workshops
3. The per workshop cost which needs to be spread across all participants attending the workshop.
4. The per participant onboarding costs

In the base case scenario we assume that workshops are not filled to capacity based on observations from real world services. We assume on average 6 people attend out of a maximum capacity of 10. We have generated a full capacity scenario in which the service is run to capacity, which decreases the overall cost-per participant. The breakdown of costs used in the micro-costing are reported in ESM Table 66.

ESM Table 66: DE micro-costing

	Base case scenario		Full capacity scenario	
	Total cost	Number of participants	Total cost	Number of participants
DESMOND Licence	£10,000	1000	£10,000	1664
License cost per person	£10		£6.01	
**CCG-wide costs for face to face service**				
Staff training payment to DESMOND	£2800	576	£2800	960
Staff training (Band 4 staff time £37 per hour)	£518	576	£518	960
Staff training Band 7 staff time £66 per hour)	£924	576	£924	960
Uniforms	£300	576	£300	960
CCG cost per person	£7.89		£4.73	
**Workshop costs**				
Staff cost (10 hours Band 4 staff time £37)	£370	6	£370	10

Equipment and materials for 6 participants	£23.94	6	£39.90	10
Venue hire (£300 per session)	£300	6	£300	10
Band 4 Diabetes educator (Band 4, £37 per hour, 7.5 hours)	£277.50	6	£277.50	10
Band 7 Diabetes educator (Band 4, £66 per hour, 7.5 hours)	£495.00	6	£495.00	10
Mileage	£15.53	6	£15.53	10
Workshop cost per person	£247.00		£149.79	
Onboarding costs				
B4 staff	£2.47	1	£2.47	1

Service cost per patient	Real word cost	Capacity cost	Proportion
Face to face cost (Licence + CCG wide costs + Workshop costs)	£264.88	£160.53	57.6%
Online cost (Licence + Online)	£12.47	£8.48	42.4%
Average cost per participant	£157.86	£96.06	

Validation

The model outputs were validated by reviewing the time traces for trajectories and events in the model. We compare the simulated incidence over time against previous recorded incidence rates in other studies where data are available. The validation is challenging because there are few study cohorts or trials that have been conducted in a comparable study population. Nevertheless, the validation exercise was useful in identifying differences and understanding whether the differences are in the right direction given the differences in population characteristics.

ESM Figure 6 illustrates the average GP utilisation over time in the model. Fluctuations in attendance are due to random number generation used in the model. The increasing trend over time is consistent with the aging and development of comorbid conditions.

ESM Figure 6: Average GP utilisation over time

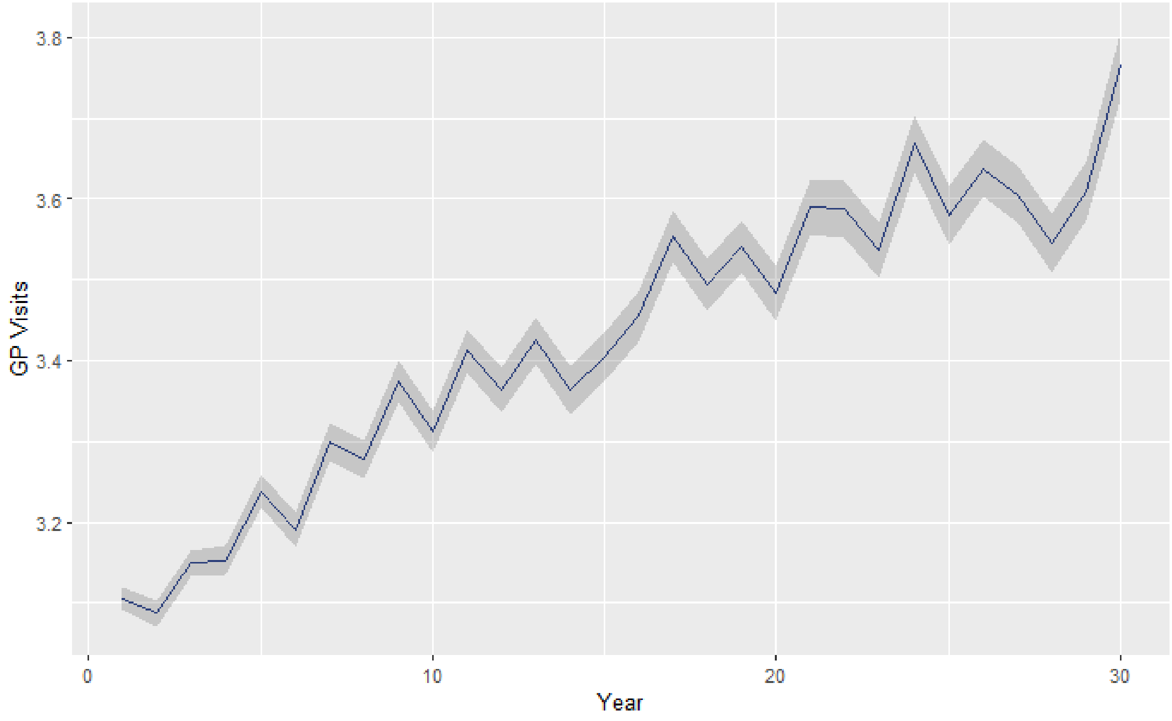

The 10-year simulated event rates reported from the UKPDS trial and the event rates simulated within the Diabetes Treatment model without calibration are in ESM Table 67. Differences are to be expected due to differing population characteristics, notably, the GLOW trial population had a higher average BMI and were older (ESM Table 68).

ESM Table 67**: Proportion of total population having suffered event in first 10-years simulated.**

(%)	UKPDS - OM2	Diabetes Treatment Model Without Calibration	Diabetes Treatment Model With Calibration
1st MI	9.9	10.3	
2nd MI	1	0.76	
1st Stroke	6.2	6.5	
2nd Stroke	0.71	0.94	
CHF	4	7.9	
IHD	7.8	9.4	
Blindness	2.9	6.0	
Ulcer	1.8	6.5	
1st Amputation	1.5	3.4	

2nd Amputation	0.44	0.65	
Renal Failure	4	3.7	
All-Cause Mortality	22.5	26.3	

ESM Table 68: Comparison of UKPDS and GLOW participant characteristics

UKPDS 56 (2001) – Table 1: Characteristics of Patients			GLOW
At diagnosis	Men (n=2643)	Women (n=1897)	All (577)
Gender (Male)	58%		48%
Age	51.5 (8.8)	52.7 (8.7)	**59.8 (12.6)**
White	81 (2151)	85 (1603)	
Afro-Caribbean	7.6 (201)	8.1 (153)	3.3%
Asian Indian	11 (291)	7.4 (141)	3.8%
Smoker	34 (898)	25 (474)	10.4%
BMI	27.7 (4.6)	30.4 (6.3)	**34.6 (6.78)**
After 1 or 2 years (?)			At baseline
HbA_{1c}	6.6 (1.4)	6.9 (1.5)	7.1%
SBP	133 (18)	139 (21)	
Total Chol	5.2 (1.0)	5.7 (1.1)	
HDL-C	1.06 (0.23)	1.18 (0.27)	

The simulated trajectories for risk factors in the model are plotted in ESM Figure 7.

ESM Figure 7: Simulated risk factor trajectories

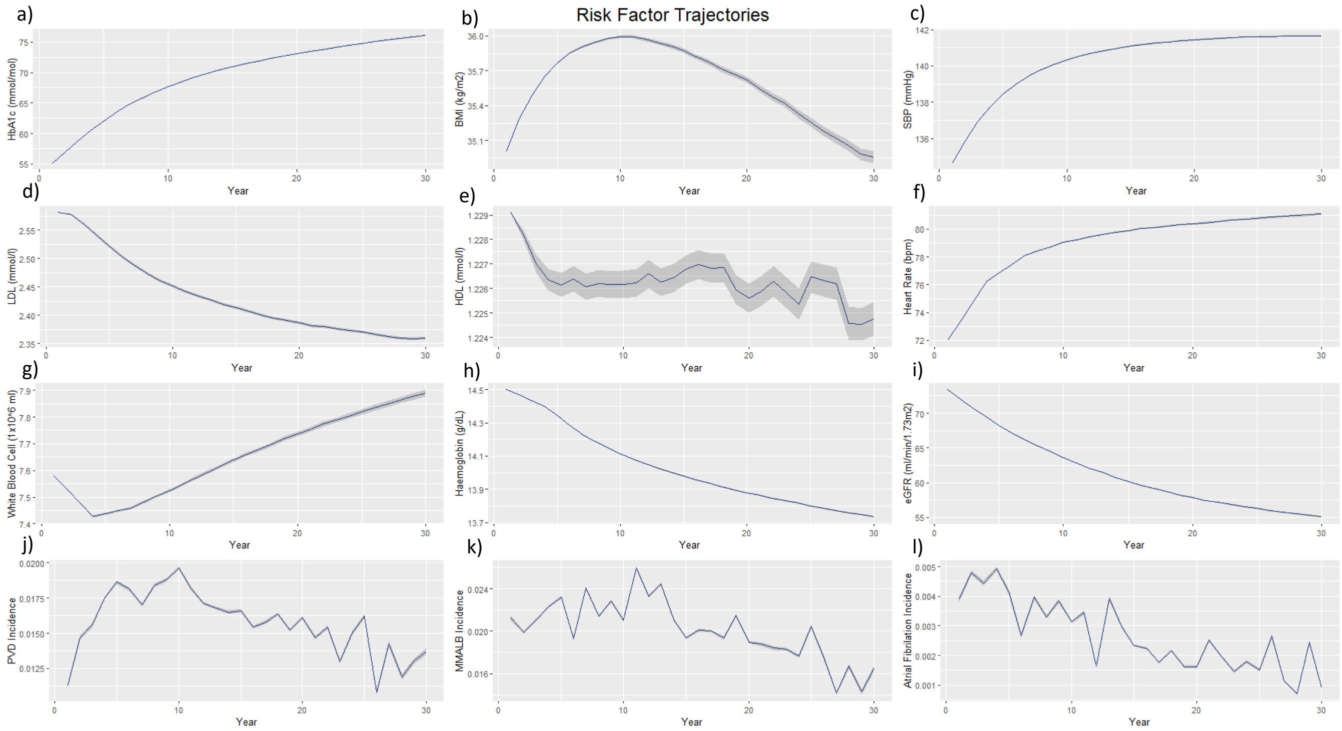

The proportion of smokers alive over time.

ESM Figure 8: Proportion of smokers alive over time

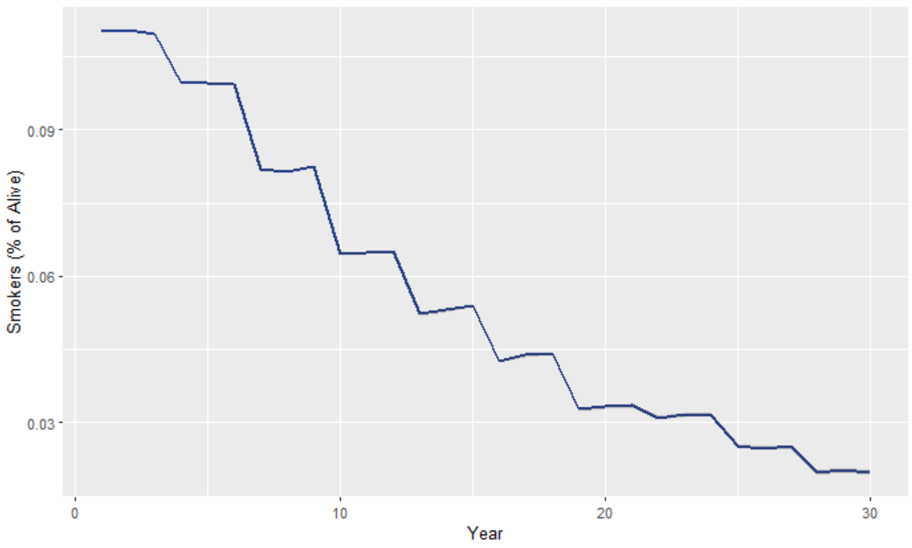

The simulated incidence of major events in the model are illustrated in ESM Figure 9.

ESM Figure 9: Simulated major health events in the Diabetes treatment model

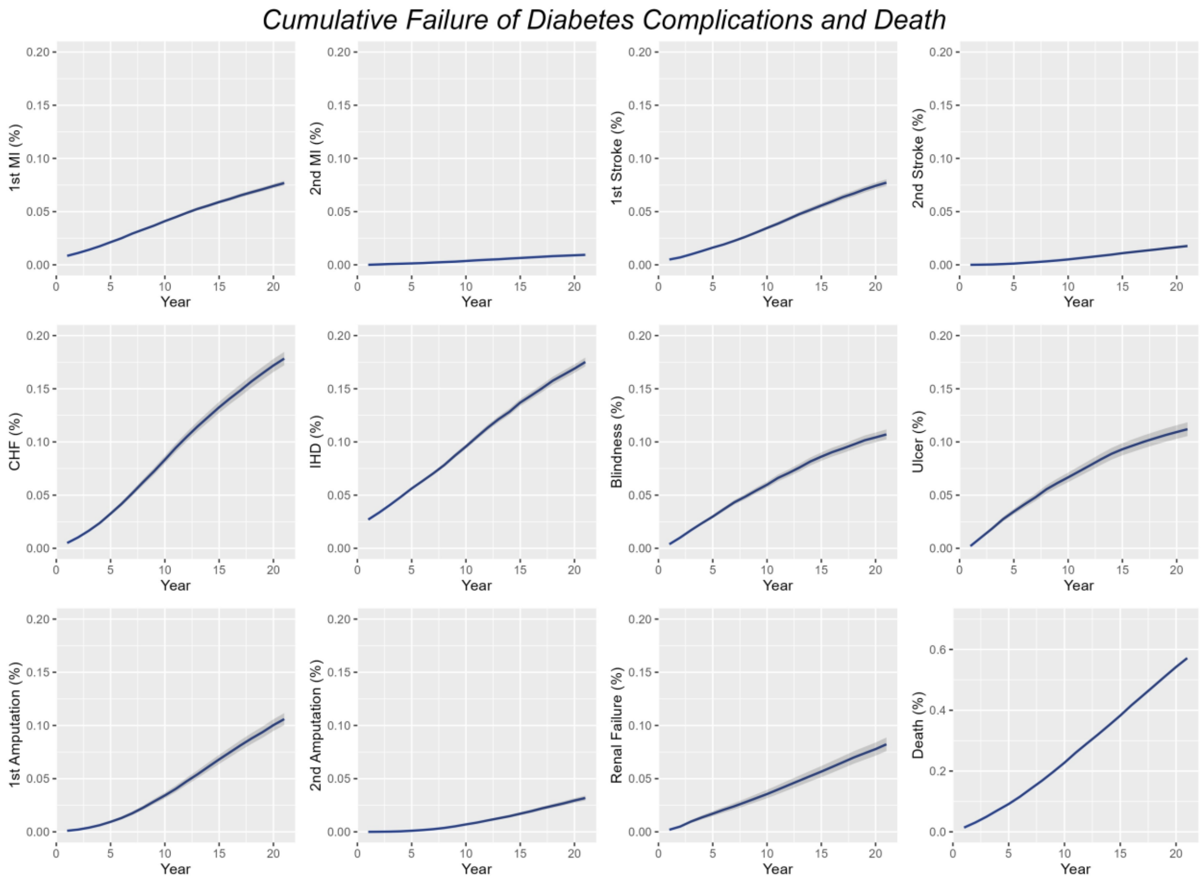

Considering the incidence of foot ulcer and Amputation appears to be high we identified additional data sources to validate this finding, a 4-year snapshot from Hospital Episode Statistics found an incidence rate of 2.51 amputations per 1000 person years in people with diabetes [56]. While not directly comparable due to differences in the populations duration of diabetes, the model simulates an incidence rate of 1.76 amputations per 1000 person-years over the first 4 years of the simulation. The model simulates an incidence rate of 4.63 amputations per 1000 person-years over the by year 10 of the simulation. We believe that the high incidence of amputations is driven by the high BMI for this population. High BMI impacts the incidence of foot ulcers and Peripheral Vascular Disease, which are risk factors for amputation. The strong temporal observation in our analysis also suggests that the peak in the incidence of amputations corresponds to the maximum simulated BMI at 10 years.

A cohort study found 1.00 incidence of end stage renal failure per 1000 person-years over the period between January 2017 and December 2019 in individuals with T2D [57]. This is compared to an incidence of 3.60 per 1000 person-years over the first 5 years of the simulation. The prevalence of kidney disease within this cohort study was 30%. A decision was made not to calibrate amputation and renal failure to lower incidence rates based on the observation that the prevalence of microvascular disease in newly diagnosed populations has been identified as being high, therefore this population at increased risk due to BMI are plausible [58].

Plots for the cumulative incidence of cancer, osteoarthritis and depression are reported in ESM Figure 10.

ESM Figure 10: Simulated cumulative incidence of cancer, depression and osteoarthritis

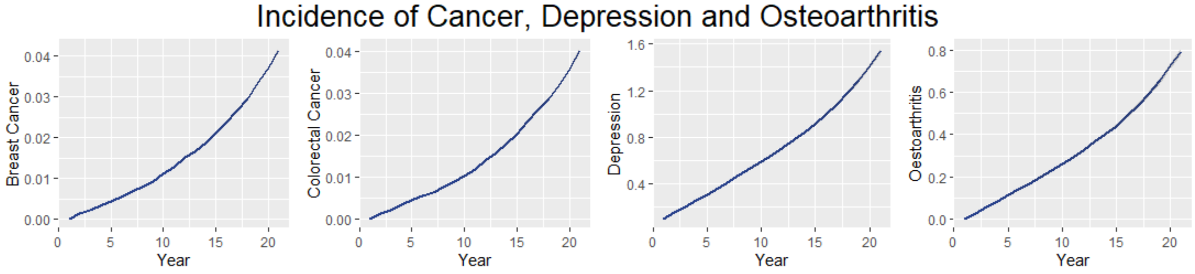

Diabetes Remission

Individuals are assumed to transition from monotherapy to being on no diabetes related drugs, or 'remission', if their HbA_{1c} drops below 6.5% [28]. This allows for a cost saving to be achieved for any individual who is assumed to come off diabetes medication at this point. The HbA_{1c} trajectories assumed in the model set HbA_{1c} to increase with time. Therefore, individuals that are in remission either start with a sampled HbA_{1c} less than 6.5% or face an HbA_{1c} that has decreased below 6.5% because of the intervention effect. Individuals receiving the DEW intervention can transfer to remission in the first year if the simulated treatment effect takes their HbA_{1c} below 6.5. The minimum HbA_{1c} in the sample population is 5%, based on the minimum HbA_{1c} level seen at baseline in the GLOW trial population. Therefore, in both the DE and the DEW arm, the same set of individuals will be in remission

in the first year. In the DEW arm, individuals who are sampled to have an HbA_{1c} higher than 6.5%, but that fell below this threshold due to the intervention make up any additional remission cases in this treatment arm. ESM Table 69 reports the number of people in remission for each arm over time, and the average time spend in remission.

ESM Table 69: Simulated diabetes remission for intervention and control over time

	DE			DEW			Difference	
	Mean HbA_{1c} (%)	Number in Remission	Total Died	Mean HbA_{1c} (%)	Number in Remission	Total Died	Number in Remission	Relative risk
Year 1	7.19	1773	127	7.09	2132	127	359	1.202
Year 2	7.35	741	258	7.25	967	258	226	1.305
Year 3	7.52	115	395	7.44	197	395	82	1.713
Year 4	7.68	0	537	7.62	4	356	4	NA
Year 5	7.83	0	722	7.79	0	721	0	0
Year 6	7.96	0	896	7.94	0	895	0	0
Year 7	8.07	0	1121	8.07	0	1120	0	0
Mean No. years in remission			0.25			0.31	0.06	
Mean No. years in remission of those entering remission in Year 1			1.49			1.54		

The model parameters did not specify the proportion of patients in remission for each treatment, but this emergent outcome can be observed and validated against the trial data. ESM Table 70 reports a validation of the modelled diabetes remission compared with the observations from the GLoW trial. The simulation slightly overpredicts the proportion in remission at 12 months, but underpredicts the difference between treatment arms.

ESM Table 70: Comparison of the simulated % in remission at 12 months compared with the trial data

	DE		DEW		Treatment Arm Difference	
12-month outcome	Model Sim	GLOW Trial	Model Sim	GLOW Trial	Model Sim	GLOW Trial
Remission (%)	17.9%	15.0%	21.3%	20.1%	3.4%	5.3%

Model Stability and Probabilistic Sensitivity Analysis

Probability sensitivity analysis (PSA) was used within the model to account for uncertainty within the model parameters. Random samples of each parameter were taken simultaneously based on a distribution determined by the mean and a standard error to accommodate this uncertainty. Based on the stability of the average expected net benefit depending on the number of random samples of each parameter taken, a total of 2000 variations of parameters were run through the model (ESM Figure 11). The results for each PSA sample were compiled.

To determine an appropriate population size, the expected net benefit estimated through the deterministic analysis was recorded for increasing number of individuals within the sample population. Based on the stability of the model outcomes, a cohort of 100,000 individuals were run through each PSA sample (ESM Figure 12).

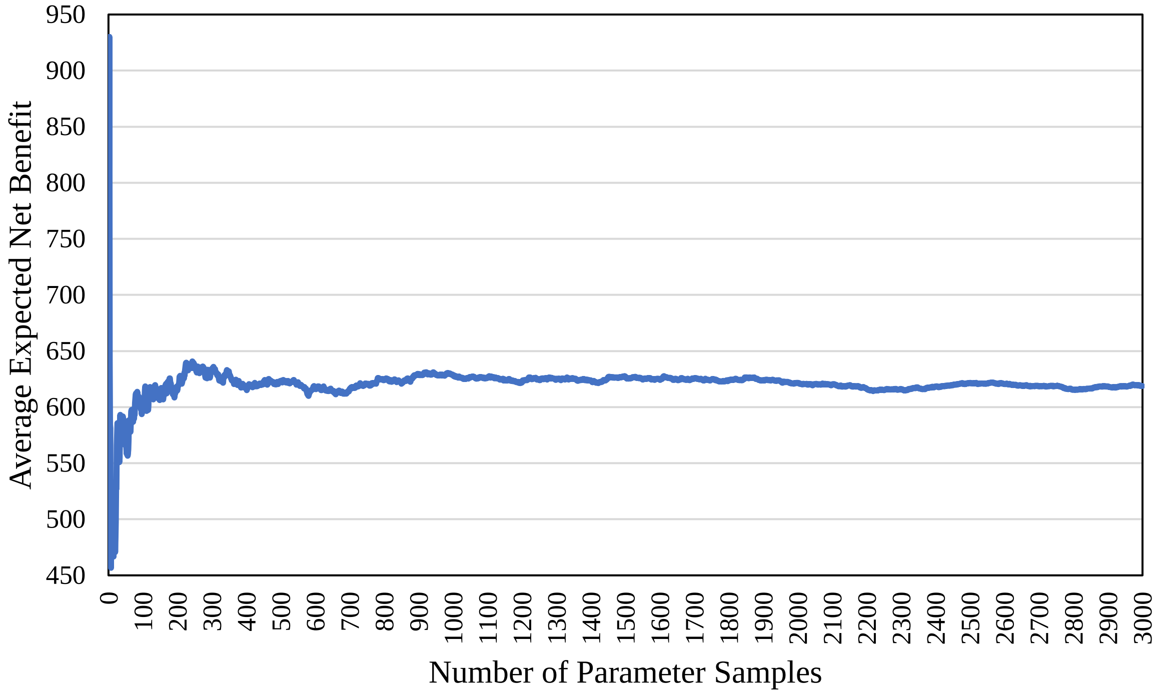

ESM Figure 11: Average expected net benefit by number of PSA runs

ESM Figure 12: Expected net benefit of a deterministic analysis by sample population size

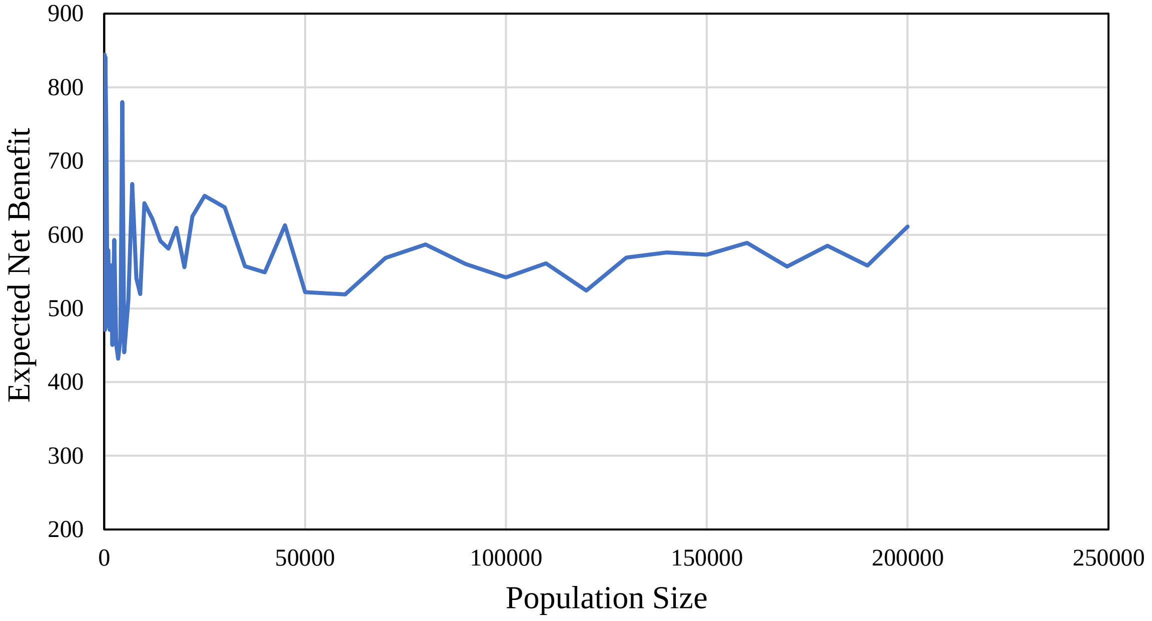

References

1. Ahern AL, W.J., Wells E, Sharp SJ, Islam N, Lawlor ER, Duschinsky R, Hill AJ, Doble B, Wilson E, Morris S., *Clinical and cost-effectiveness of a diabetes education and behavioural weight management programme versus a diabetes education programme in adults with a recent diagnosis of type 2 diabetes: Study protocol for the Glucose Lowering through Weight management (GLoW) randomised controlled trial.* . BMJ open, 2020. **10**(4): p. e035020.
2. National Institute for Health and Care Excellence *NG28 Health Economic Model Report*. 2015.
3. The Health Improvement Network (THIN). *The Health Improvement Network (THIN) (2014)*.
4. Kearns, B., Rafia, R., Leaviss, J., Preston, L., Wong, R., Brazier, J. E., Palmer, S., Ara, R. *Whole pathway modelling of depression in patients. Theme 2: Mental Health. Part 2: Independent economic evaluation: methods and results, discussion and conclusion.* EEPRU Research Report 043. , 2014.
5. National Institute for Health and Care Excellence *NG28 Health Economic Model Report: Appendix F*. 2015.
6. EBMcalc. *Glycemic Assessment: A1C to Average Glucose Conversions*. 18.10.2023]; Available from: https://ebmcalc.com/GlycemicAssessment.htm#:~:text=A1C(%25)%20%3D%20(A1C(mmol,mol)%20%2F%2010.929)%20%2B%202.15.
7. Leal J, A.M., Gregory V, Hayes A, Mihaylova B, Gray AM, Holman RR, Clarke P., *Estimating risk factor progression equations for the UKPDS Outcomes Model 2 (UKPDS 90).* Diabetic Medicine., 2021. **38**(10): p. e14656.
8. Hayes, A.J., et al., *UKPDS outcomes model 2: a new version of a model to simulate lifetime health outcomes of patients with type 2 diabetes mellitus using data from the 30 year United Kingdom Prospective Diabetes Study: UKPDS 82.* Diabetologia, 2013. **56**(9): p. 1925-1933.
9. Yebyo HG, A.H., Kaufmann M, Puhan MA., *Comparative effectiveness and safety of statins as a class and of specific statins for primary prevention of cardiovascular disease: A systematic review, meta-analysis, and network meta-analysis of randomized trials with 94,283 participants.* American heart journal., 2019. **1**(210): p. 18-28.
10. Brunström M, C.B., *Effect of antihypertensive treatment at different blood pressure levels in patients with diabetes mellitus: systematic review and meta-analyses.* BMJ, 2016. **352**.

11. Khaw, K.T., et al., *Glycated haemoglobin, diabetes, and mortality in men in Norfolk cohort of european prospective investigation of cancer and nutrition (EPIC-Norfolk).* BMJ, 2001. **322**(7277): p. 15-18.
12. Lahmann, P.H., et al., *Body size and breast cancer risk: findings from the European Prospective Investigation into Cancer And Nutrition (EPIC).* Int. J. Cancer, 2004. **111**(5): p. 762-771.
13. Pischon, T., et al., *Body size and risk of colon and rectal cancer in the European Prospective Investigation Into Cancer and Nutrition (EPIC).* J. Natl. Cancer Inst, 2006. **98**(13): p. 920-931.
14. Renehan, A.G., et al., *Body-mass index and incidence of cancer: a systematic review and meta-analysis of prospective observational studies.* Lancet, 2008. **371**(9612): p. 569-578.
15. Schett, G., et al., *Diabetes is an independent predictor for severe osteoarthritis: results from a longitudinal cohort study.* Diabetes Care, 2013. **36**(2): p. 403-409.
16. Palmer, A.J., et al., *The CORE Diabetes Model: Projecting long-term clinical outcomes, costs and cost-effectiveness of interventions in diabetes mellitus (types 1 and 2) to support clinical and reimbursement decision-making.* Curr. Med. Res. Opin, 2004. **20**(Suppl. 1): p. S5-S26.
17. Golden, S.H., et al., *Examining a bidirectional association between depressive symptoms and diabetes.* JAMA, 2008. **299**(23): p. 2751-2759.
18. Whyte, E.M., et al., *Depression after stroke: a prospective epidemiological study.* J. Am. Geriatr. Soc, 2004. **52**(5): p. 774-778.
19. Tao L, W.E., Griffin SJ, Simmons RK, ADDITION-Europe Study Team. , *Performance of the UKPDS outcomes model for prediction of myocardial infarction and stroke in the ADDITION-Europe trial cohort.* Value in health. , 2013. **16**(6): p. 1074-80.
20. Keng, M.J., et al., *Performance of the UK prospective diabetes study outcomes model 2 in a contemporary UK type 2 diabetes trial cohort.* Value in Health, 2022. **25**(3): p. 435-442.
21. Laxy, M., et al., *Performance of the UKPDS Outcomes Model 2 for predicting death and cardiovascular events in patients with type 2 diabetes mellitus from a German population-based cohort.* Pharmacoeconomics, 2019. **37**: p. 1485-1494.
22. Pagano, E., et al., *Prediction of mortality and major cardiovascular complications in type 2 diabetes: external validation of UK Prospective Diabetes Study outcomes model version 2 in two European observational cohorts.* Diabetes, Obesity and Metabolism, 2021. **23**(5): p. 1084-1091.
23. de Jong, M., M. Woodward, and S.A. Peters, *Duration of diabetes and the risk of major cardiovascular events in women and men: A prospective cohort study of UK Biobank participants.* Diabetes Research and Clinical Practice, 2022. **188**: p. 109899.
24. Shewmaker P, C.S., Iskandar R, Lake D, Jutkowitz E. , *Microsimulation model calibration with approximate bayesian computation in r: A tutorial. .* Medical Decision Making., 2022. **42**(5): p. 557-70.
25. Griffin SJ, R.G., Khunti K, Witte DR, Lauritzen T, Sharp SJ, Dalsgaard EM, Davies MJ, Irving GJ, Vos RC, Webb DR., *Long-term effects of intensive multifactorial therapy in individuals with screen-detected type 2 diabetes in primary care: 10-year follow-up of the ADDITION-Europe cluster-randomised trial.* The lancet Diabetes & endocrinology. , 2019. **7**(12): p. 925-37.
26. Jones KC, B.A., *Unit costs of health and social care.*, in *PSSRU*, PSSRU, Editor. 2021: PSSRU.
27. *National Cost Collection - National Schedule of NHS Costs 2020/21*, in *NHS Digital*, N. Digital, Editor. 2022.
28. National Institute for Health and Care Excellence *Type 2 Diabetes in adults: management*. 2015.
29. Bennett H, M.P., Bergenheim K, Gordon J., *Assessment of unmet clinical need in type 2 diabetic patients on conventional therapy in the UK.* Diabetes Therapy., 2014. **5**: p. 567-78.
30. *British National Formularly*. https://bnf.nice.org.uk/, 2022.
31. Poole, C., et al., *The prescription cost of managing people with type 1 and type 2 diabetes following initiation of treatment with either insulin glargine or insulin determir in routine*

general practice in the UK: a retrospective database analysis. Current Medical Research and Opinion, 2007. **23**(1): p. S41-S48.
32. Alva, M.L., Gray, A., Mihaylova, B., Leal, J. and Holman, R.R., *The impact of diabetes-related complications on healthcare costs: new results from the UKPDS (UKPDS 84).* Diabetic medicine., 2015. **32**(4): p. 459-66.
33. National Institute for Health and Care Excellence, *Hypertension in adults: diagnosis and management. Cost effectiveness analysis: Treatment initiation threshold for people with stage 1 hypertension.*, in *National Institute for Health and Care Excellence*. 2019.
34. Kerr M, B.E., Chadwick P, Evans T, Kong WM, Rayman G, Sutton-Smith M, Todd G, Young B, Jeffcoate WJ., *The cost of diabetic foot ulcers and amputations to the National Health Service in England.* Diabetic Medicine., 2019. **36**(8): p. 995-1002.
35. *The economic costs of arthritis for the UK economy*. Oxford Economics, 2014.
36. Laudicella M, W.B., Burns E, Smith PC., *Cost of care for cancer patients in England: evidence from population-based patient-level data. .* British journal of cancer. , 2016. **114**(11): p. 1286-1292.
37. Chalder, M., et al., *A pragmatic randomised controlled trial to evaluate the cost-effectiveness of a physical activity intervention as a treatment for depression: the treating depression with physical activity (TREAD) trial.* Health Technol. Assess, 2012. **16**(10): p. 1-iv.
38. Grieve, R., et al., *A comparison of the cost-effectiveness of stroke care provided in London and Copenhagen.* Int. J. Technol. Assess. Health Care, 2000. **16**(2): p. 684-695.
39. Curtis, L., *Unit costs of health and social care*. 2017.
40. Devlin NJ, S.K., Feng Y, Mulhern B, van Hout B., *Valuing health-related quality of life: An EQ-5 D-5 L value set for E ngland. .* Health economics., 2018. **27**(1): p. 7-22.
41. Ara R, B.J., *Populating an economic model with health state utility values: moving toward better practice.* Value in Health, 2010. **13**(5): p. 509-18.
42. Breeze, P., et al., *Estimating the impact of changes in weight and BMI on EQ-5D-3L: a longitudinal analysis of a behavioural group-based weight loss intervention.* Quality of Life Research, 2022. **31**(11): p. 3283-3292.
43. Hayes A, A.H., Woodward M, Chalmers J, Poulter N, Hamet P, Clarke P., *Changes in quality of life associated with complications of diabetes: results from the ADVANCE study.* Value in health., 2016. **19**(1): p. 36-41.
44. Ara, R. and A. Wailoo, *NICE DSU Technical Support Document 12: The use of health state utility values in decision models*. 2011.
45. Bagust A, B., S, *Modelling EuroQol health-related utility values for diabetic complications from CODE-2 data.* Health economics., 2005. **14**(3): p. 217-30.
46. Alva, M., et al., *The Effect of Diabetes Complications on Health-Related Quality of Life: The importance of longitudinal data to address patient heterogeneity.* Health economics., 2014. **23**(4): p. 487-500.
47. Yabroff, K.R., et al., *Burden of illness in cancer survivors: findings from a population-based national sample.* J. Natl. Cancer Inst, 2004. **96**(17): p. 1322-1330.
48. Black, C., et al., *The clinical effectiveness of glucosamine and chondroitin supplements in slowing or arresting progression of osteoarthritis of the knee: a systematic review and economic evaluation.* Health Technol. Assess, 2009. **13**(52): p. 1-148.
49. Benedict, A., et al., *Economic evaluation of duloxetine versus serotonin selective reuptake inhibitors and venlafaxine XR in treating major depressive disorder in Scotland.* J. Affect. Disord, 2010. **120**(1-3): p. 94-104.
50. Higgins JPT, T.J., Chandler J, Cumpston M, Li T, Page MJ, Welch VA (editors). *Cochrane Handbook for Systematic Reviews of Interventions: Chapter 6, Section 6.3.1.* 2023, Cochrane.
51. Franz MJ, B.J., Rutten-Ramos S, VanWormer JJ., *Lifestyle weight-loss intervention outcomes in overweight and obese adults with type 2 diabetes: a systematic review and meta-analysis*

of randomized clinical trials. . Journal of the Academy of Nutrition and Dietetics. , 2015. **115**(9): p. 1447-1463.

52. Esposito K, M.M., Ciotola M, Di Palo C, Scognamiglio P, Gicchino M, Petrizzo M, Saccomanno F, Beneduce F, Ceriello A, Giugliano D., *Effects of a Mediterranean-style diet on the need for antihyperglycemic drug therapy in patients with newly diagnosed type 2 diabetes: a randomized trial.* . Annals of internal medicine. , 2009. **151**(5): p. 306-314.
53. Cradock KA, Ó.G., Finucane FM, Gainforth HL, Quinlan LR, Ginis KA. , *Behaviour change techniques targeting both diet and physical activity in type 2 diabetes: A systematic review and meta-analysis.* . International Journal of Behavioral Nutrition and Physical Activity., 2017. **14**(1): p. 1-7.
54. Look AHEAD Research Group, *Eight-year weight losses with an intensive lifestyle intervention: the look AHEAD study.* Obesity., 2014. **22**(1): p. 5-13.
55. Ahern, A.L., et al., *Effectiveness and cost-effectiveness of referral to a commercial open group behavioural weight management programme in adults with overweight and obesity: 5-year follow-up of the WRAP randomised controlled trial.* The Lancet Public Health, 2022. **7**(10): p. e866-e875.
56. Holman, N., R. Young, and W. Jeffcoate, *Variation in the recorded incidence of amputation of the lower limb in England.* Diabetologia, 2012. **55**: p. 1919-1925.
57. Cook, S., et al., *Characterising the burden of chronic kidney disease among people with type 2 diabetes in England: a cohort study using the Clinical Practice Research Datalink.* BMJ open, 2023. **13**(3): p. e065927.
58. Palladino, R., et al., *Association between pre-diabetes and microvascular and macrovascular disease in newly diagnosed type 2 diabetes.* BMJ Open Diabetes Research and Care, 2020. **8**(1): p. e001061.
